# Evaluation of Cytotoxicity and α-Glucosidase Inhibitory Activity of Amide and Polyamino-Derivatives of Lupane Triterpenoids

**DOI:** 10.3390/molecules25204833

**Published:** 2020-10-20

**Authors:** Oxana B. Kazakova, Gul’nara V. Giniyatullina, Akhat G. Mustafin, Denis A. Babkov, Elena V. Sokolova, Alexander A. Spasov

**Affiliations:** 1 Ufa Institute of Chemistry of the Ufa Federal Research Centre of the Russian Academy of Sciences, 71 pr. Oktyabrya, 450054 Ufa, Russia; gulnaravlg@gmail.com (G.V.G.); agmustafin@gmail.com (A.G.M.); 2 Laboratory of Metabotropic Drugs, Scientific Center for Innovative Drugs, Volgograd State Medical University, Novorossiyskaya st. 39, 400087 Volgograd, Russia; dababkov@volgmed.ru (D.A.B.); sokolova210795@gmail.com (E.V.S.)

**Keywords:** lupane triterpenoids, betulinic acid, betulonic acid, polyamine, squalamine, trodusquemine, spermidine, cytotoxicity, NCI-60 cancer cell line panel, α-glucosidase, antimicrobial, CellMinner, Gene Ontology

## Abstract

A series of two new and twenty earlier synthesized branched extra-amino-triterpenoids obtained by the direct coupling of betulinic/betulonic acids with polymethylenpolyamines, or by the cyanoethylation of lupane type alcohols, oximes, amines, and amides with the following reduction were evaluated for cytotoxicity toward the NCI-60 cancer cell line panel, α-glucosidase inhibitory, and antimicrobial activities. Lupane carboxamides, conjugates with diaminopropane, triethylenetetramine, and branched C3-cyanoethylated polyamine methyl betulonate showed high cytotoxic activity against most of the tested cancer cell lines with GI_50_ that ranged from 1.09 to 54.40 µM. Betulonic acid C28-conjugate with triethylenetetramine and C3,C28-bis-aminopropoxy-betulin were found to be potent micromolar inhibitors of yeast α-glucosidase and to simultaneously inhibit the endosomal reticulum α-glucosidase, rendering them as potentially capable to suppress tumor invasiveness and neovascularization, in addition to the direct cytotoxicity. Plausible mechanisms of cytotoxic action and underlying disrupted molecular pathways were elucidated with CellMinner pattern analysis and Gene Ontology enrichment analysis, according to which the lead compounds exert multi-target antiproliferative activity associated with oxidative stress induction and chromatin structure alteration. The betulonic acid diethylentriamine conjugate showed partial activity against methicillin-resistant *S. aureus* and the fungi *C. neoformans*. These results show that triterpenic polyamines, being analogs of steroidal squalamine and trodusquemine, are important substances for the search of new drugs with anticancer, antidiabetic, and antimicrobial activities.

## 1. Introduction

Pentacyclic triterpenoids are natural products which are widespread in the plant kingdom and are useful substrates for the synthesis of various compounds with important bioactivities [1,2,3]. The introduction of nitrogen-containing substituents into a triterpenoid scaffold resulted in a series of anticancer, antimicrobial, and antiviral agents [4,5,6,7,8,9,10,11]. Conjugates of steroids and polyamines have been actively investigated in recent years as potential new cationic steroidal antibiotics [12]. The most known compound is squalamine, which has been shown to inhibit mitogen-induced proliferation and the migration of endothelial cells in vitro and caused significant in vivo inhibition of angiogenesis [13]. This steroidal broad-spectrum antibiotic exhibits activity against Gram-positive and Gram-negative bacteria, fungi, and viruses [14]. Trodusquemine (spermine analog of squalamine) has tremendous potential both as an antiobesity and an antidiabetic pharmacotherapeutic [15]. The high biological activity of squalamine stimulated numerous studies on the design and synthesis of its analogs, including monoterpene [16] and triterpene-based derivatives [17,18,19,20,21]. Some triterpene–spermine conjugates form dynamic supramolecular networks in solutions which influence their cytotoxicity [22].

The main chemical modifications of triterpenic acids with polyamines are based on direct coupling with commercially available di- and tri-amines [4,8,11], as well as 1,4,7,10-tetraazacyclododecane-1,4,7,10-tetraacetic acid (DOTA) oligo-methylene amines [8]. On the other hand, we have successfully introduced a polyamine chain into the triterpene core by the reactions of cyanoethylation followed by a reduction of the nitrile group based on triterpenic alcohols, oximes, amines, and amides, and this approach allowed us to obtain a broad library of branched extra-amino-triterpenoids [6,9,17,18,20,23,24]. In this paper, we focused on the evaluation of their cytotoxicity toward the NCI-60 cancer cell line panel, α-glucosidase inhibitory, and antimicrobial activities, as well as synthesis of two new lupane carboxamides. We were interested if triterpenic polyamine derivatives can become an alternative group of polyvalent compounds, as was established for squalamine and trodusquemine, and to open a new area of their detailed study.

## 2. Results and Discussion

### 2.1. Chemistry

**Scheme 1:**
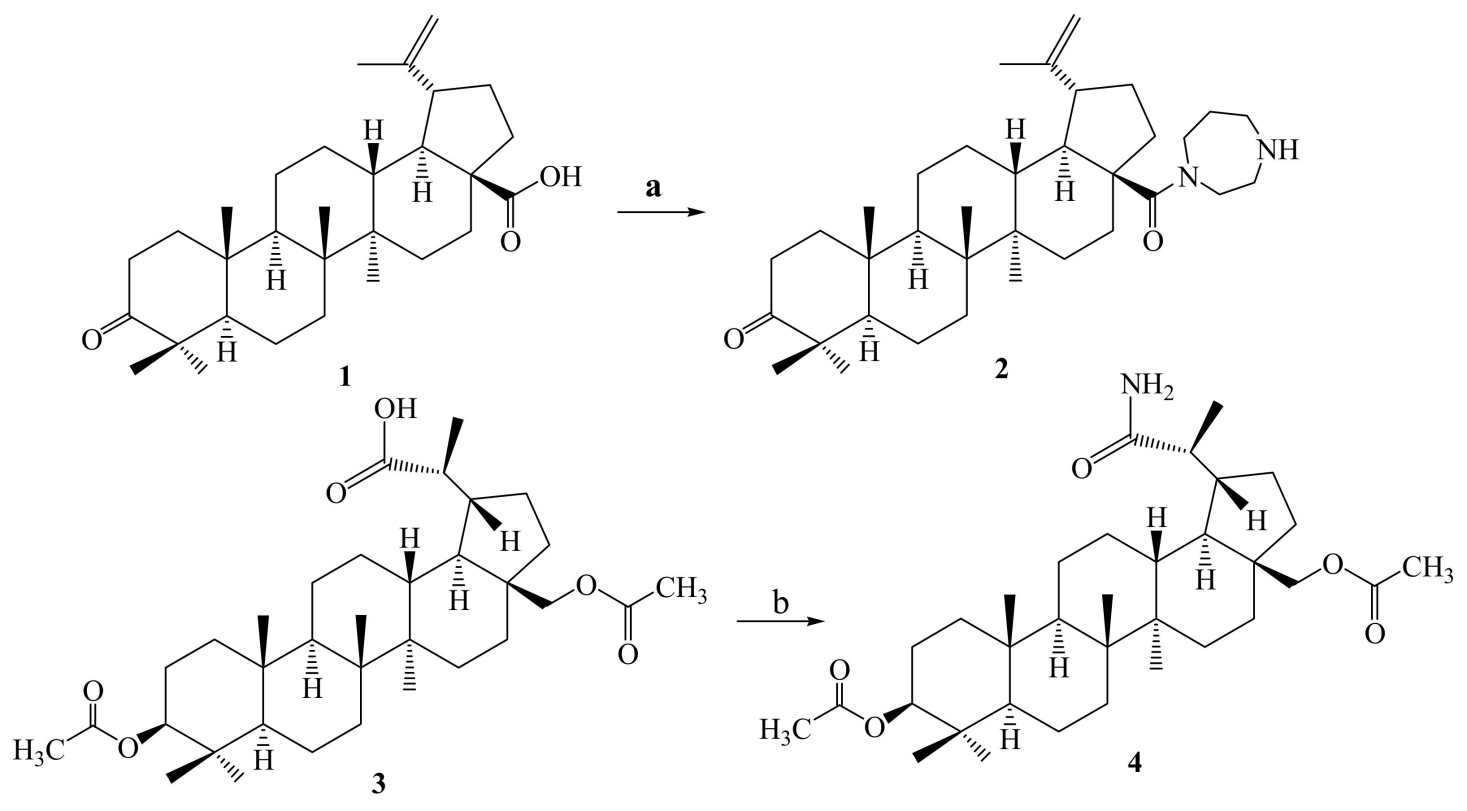
Synthesis of compounds **2** and **4**. Reagents and conditions: (a) 1. (COCl)_2_, CH_2_Cl_2_, 25 °С, 1 h; 2. homopiperazine, CHCl_3_, 22 °С, 2 h; (b) 1. (COCl)_2_, CH_2_Cl_2_, 25 °С, 1 h; 2. NH_3_, CHCl_3_, 22 °С, 6 h. Compound **3 [25]**.

**Figure 1:**
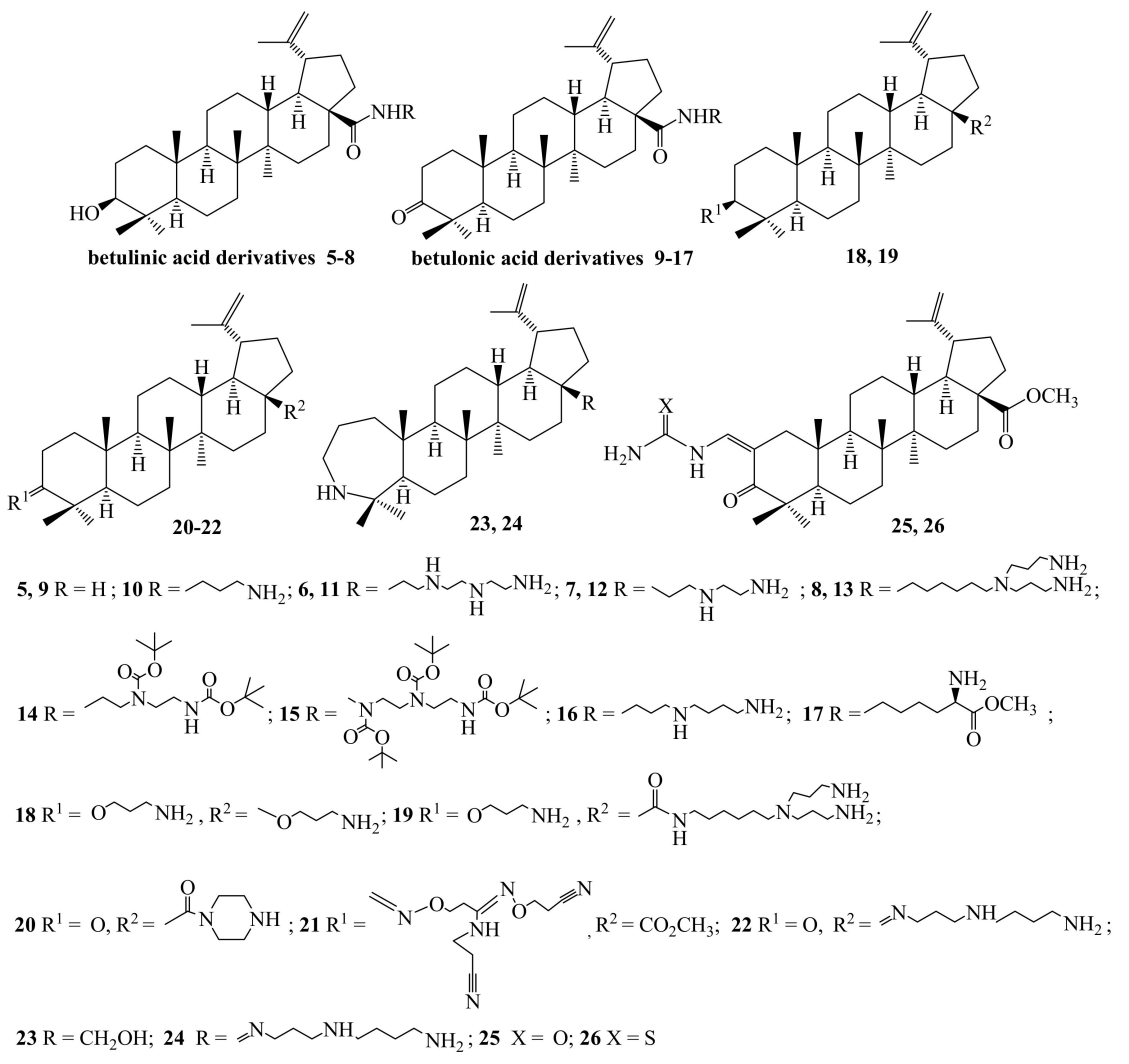
Compound **5 [26]**; compounds **6**, **7**, **11**, **12**, **14**, **15**, **16**, **19 [23]**; compounds **8**, **9**, **13 [18]**; **10 [27]**; **17 [28]**; **18, 25**, **26 [6]**; **20**, **21 [20]**; **22, 24 [24]**; **23 [9]**.

Two new lupane type amides **2** and **4** were synthesized by the acid chloride method from betulonic **1** and 3β,28-diacetoxy-(20*R*)-lupane-29-oic **3 [25]** acids through the interaction with homopiperazine or liquid ammonia (Scheme 1). The target derivatives were purified by column chromatography in good yields and their structure was confirmed by the NMR spectra, in which the signals of amide bond were found at δ 4.00 and 5.55 ppm in ^1^Н NMR spectra, and δ 174.51 and 177.56 ppm in ^13^С NMR spectra, respectively. As mentioned above, a series of branched extra-amino-triterpenoids **5–26** were synthesized according to already described methods [6,9,18,20,23,24,26,27,28] (Figure 1). These compounds are presented by C2, C3, or C28-derivatives of betulin, betulinic, and betulonic acids, methyl betulonate, or azepanobetulin. The lupane triterpene core is coupled with di- and polymethylenepolyamines, L-lysine, or piperazine. For the compounds **8**, **13**, **18**, **19**, and **21**, the polyamine chain was introduced by the cyanoethylation with the following reduction of the nitrile group. A total of twenty-two lupane triterpenoids with amide/alkane polyamino-fragments were evaluated for cytotoxicity toward the NCI-60 cancer cell line panel, antimicrobial activity, and as inhibitors of α-glucosidase. 

### 2.2. Biological Evaluation 

#### 2.2.1. Evaluation of In Vitro Antiproliferative Activity by NCI

In vitro one-dose (10^−5^ M) anticancer assays (http://www.dtp.nci.nih.gov) of compounds **2**, **4–22**, and **24–26** were performed using a full panel of about 60 human tumor cell lines representing nine different types of human cancers: leukemia, melanoma, lung, colon, central nervous system (CNS), ovarian, renal, prostate and breast cancers, in accordance with the protocol of the Drug Evaluation Branch, National Cancer Institute (NCI), Bethesda, and described elsewhere [29,30,31]. Results for each tested compound were reported as the percentage of growth of the treated cells when compared to the untreated control cells (negative numbers indicate cell death) (see Supplementary Materials and Table 1).

According to the NCI criteria (reduction of the growth of any one of the cancer cell lines to ca. 32% or less), compounds **7**, **12**, **13**, **15–17**, **20**, **22,** and **26** did not show cytotoxic activity against the studied cell lines. Compounds **2**, **9**, **24,** and **25** displayed mild sensitivity against few cell lines (leukemia SR and HL-60(TB)). Amide **8** inhibited the cell growth of the colon cancer cell line HT29 and breast cancer MDA-MB-231/ATCC. Betulonic acid *Boc*-diethylentriamine conjugate **14** showed activity against leukemia cell lines CCRF-CEM, HL-60(TB), MOLT-4, RPMI-8226, non-small cell lung cancer HOP-92, NCI-460, colon cancer HCT-116, HT29, SW-620, CNS cancer SF-295, SNB-75, U251, melanoma SK-MEL-5, renal cancer ACHN, CAKI-1, UO-31, prostate cancer PC-3, and breast cancer MCF7, MDA-MB-231/ATCC, and T-47D, whereas C3-aminopropoxy-betulinic acid polyamine conjugate **19** was active towards leukemia CCRF-CEM, K-562, MOLT-4, RPMI-8226, and SR, non-small cell lung cancer NCI-460, colon cancer COLO 205, HCT-116, HT29, KM12, SW-620, CNS cancer U-251, melanoma LOX IMVI, ovarian cancer IGROV1, OVCAR-8, and breast cancer MCF7 (Table 1).

Lupane carboxamides **4**, **5**, and betulinic acid diethylentriamine conjugate **6** showed the greatest antiproliferative activity towards all 59 cell lines, resulting in 57 cases of cancer cell lethality from −8.82% to −97.54% for compound **4**, in 49 cases of cancer cell lethality from −1.70% to −100% for compound **5** and 57 cases of cancer cell lethality from −4.72% to −100% for compound **6**. Betulonic acid conjugates with 1,3-diaminopropane **10** and triethylenetetramine **11**, as well as cyanoethylated amidoxime **21** were somewhat less active and exhibited the cell growth of 58 (48 cases of cancer cell lethality from −2.14% to −100%), 52 (50 cases of cancer cell lethality from −4.36% to −96.35%) and 50 (11 cases of cancer cell lethality from −0.38% to −84.68%) cell lines, respectively (Table 2).

Compounds **4–6**, **10**, **11**, and **21,** which possessed considerable anti-proliferative activity (Table 2), were selected for an advanced assay against a full panel (approximately 60 cell lines) at five concentrations at 10-fold dilution (100, 10, 1, 0.1, and 0.001 µM). A 48-h continuous drug exposure protocol was used and a sulforhodamine B (SRB) [31] protein assay was used to estimate cell growth. The result of tested compound is given by three response parameters (GI_50_, TGI and LC_50_) for each cell line from log concentration versus % growth inhibition curves on nine cancer disease (see Supplementary Materials). The GI_50_ value (growth inhibitory activity) corresponds to the concentration of the compound causing a 50% decrease in net cell growth (Table 3).

Among the tested compounds, the highest cytotoxic activity in five-dose testing mode screening was observed for the betulinic acid amide **5** with GI_50_ ranges from 1.09 μM to 13.20 μM against all panels of NCI60. Thus, its GI_50_ value was 1.97 μM against colon cancer cell line HCT-116; 1.78 μM against CNS cancer cell line SF-539; 1.77 μM and 1.09 μM against melanoma cancer cell lines LOX IMVI and SK-MEL-28, respectively; 1.98 μM against renal cancer cell line CAKI-1; and a GI_50_ of 1.97 μM was observed against breast cancer cell line MDA-MB-468. Compound **4** exhibited activities with GI_50_ ranges from 1.10 μM to 54.40 μM against all panels of NCI60 and the highest cytotoxic activity was against non-small cell lung cancer cell line HOP-62. For compound **6** (GI_50_ ranges from 2.95 μM to 20.10 μM) the best value was against prostate cancer cell line PC-3, for compound **10** (GI_50_ ranges from 6.95 μM to 38.90 μM)—against colon cancer cell line HT29, for compound **11** (GI_50_ ranges from 5.60 μM to 50.0 μM)—renal cancer RXF 393 and compound **21** exhibited remarkable activity against breast cancer cell line MDA-MB-468 (GI_50_ ranges from 2.02 μM to 50.0 μM) (see the Supplementary Materials). 

In general, lupane amides, conjugates with di- and polymethylenepolyamines (compounds **4–6**, **10**, **11**) and cyanoethylated derivative (compound **21**) showed high cytotoxic activity against the most of the tested cancer cell lines. The pronounced activity was observed for amides **4** and **5**, while derivatives with polyamine chain **10** and **21** showed less inhibition. Comparison of cytotoxicity of betulinic and betulonic acid conjugates with triethylenetetramine **6** and **11** showed that 3-oxo-group is more important, except some cell lines (leukemia CCRF-CEM, HL-60 (TB), K-562, RPMI-8226, colon cancer HCT-116, KM12, melanoma SK-MEL-2, renal cancer A498, ACHN, and prostate cancer PC-3) (Table 3).

Furthermore, a mean graph midpoint (MG-MID) is calculated giving an averaged activity parameter over all cell lines. The compounds **4–6**, **10**, **11**, and **21** showed GI_50_-MID values of 7.87, 3.67, 12.15, 20.46, 9.17, and 10.25 µM respectively (Table 4). The criterion for the selectivity of these compounds depends upon the ratio obtained by dividing the full panel MID (the average sensitivity of all cell lines towards the test agent) by their individual subpanel MID (the average sensitivity of all cell lines of a particular subpanel towards test agent). Ratio of 3–6 refer to moderate selectivity, ratios greater than six indicate high selectivity towards the corresponding cell line, while compounds not meeting either of these criteria are rated as nonselective [32]. In this context, the tested compounds were found to be nonselective with broad spectrum antitumor activity against the nine tumor subpanels tested with selectivity ratios ranges of 0.49–2.22.

**Figure 2:**
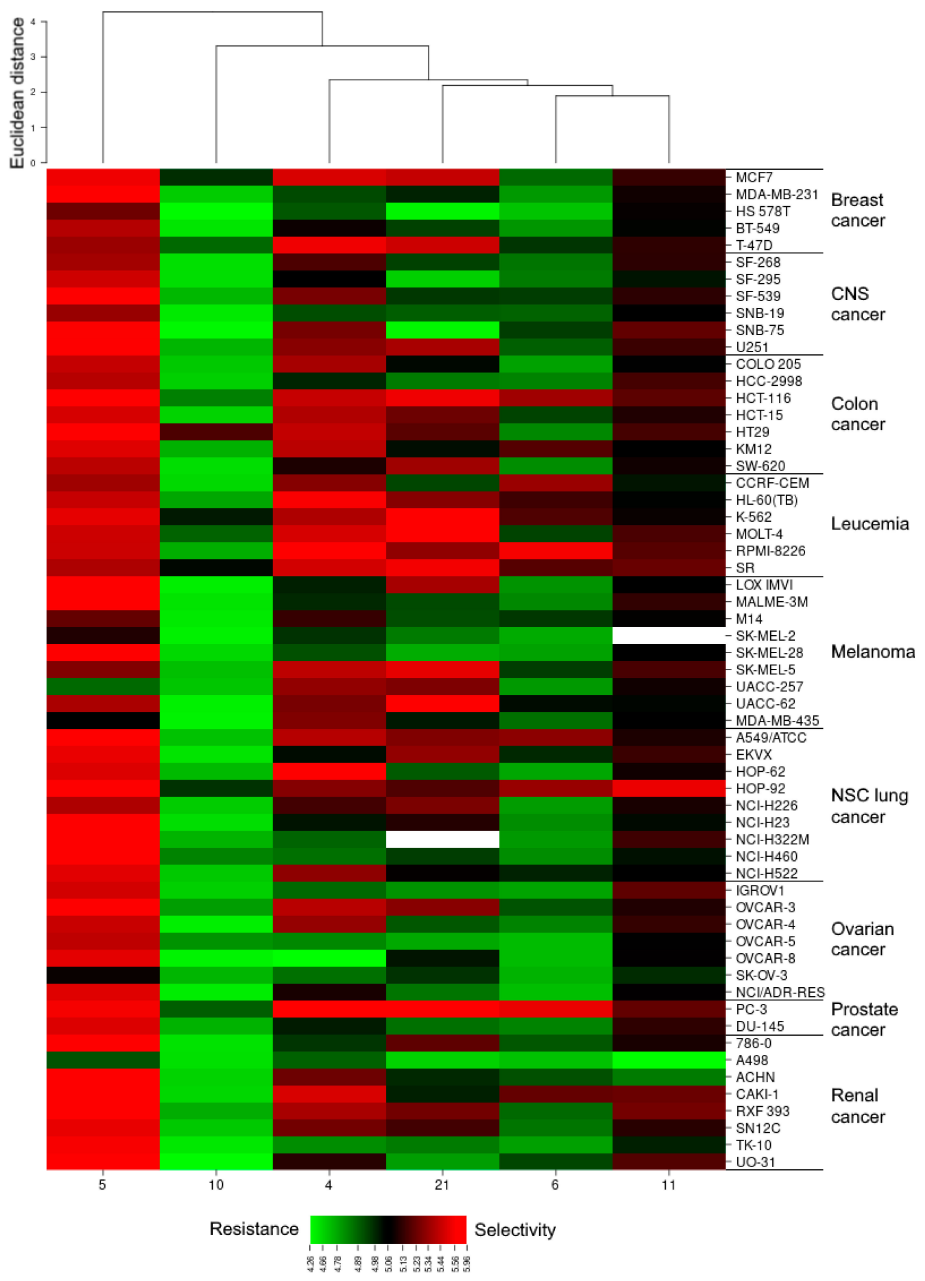
Heatmap for pGI_50_ values of compounds **4**–**6**, **10**, **11** and **21** obtained in the NCI-60 screen. The color gradient ranges from green (low activity) to red (high activity). White cells indicate the absence of data for the corresponding cell line. The hierarchical clustering of pGI_50_ activity patterns was done using the Euclidian distance method and the average linkage cluster algorithm.

To compare the patterns of cancer cell line sensitivity towards the most potent compounds **4–6**, **10**, **11** and **21** a heatmap for pGI50 values was generated (Figure 2). Euclidean distance analysis revealed that patterns of cancer cell growth inhibition significantly differ between all the hit compounds, suggesting that they exploit different mechanisms of antiproliferative activity. For example, the most potent compound **5** shares virtually no similarity with activity patterns of other agents. Compounds **4** and **21** are both relatively selective against five out of six leukemia and, to a lesser extent, colon cancer cell lines, as well as MCF7 and T47D breast cancer, and PC-7 prostate cancer cells. Overall, HCT-116, RPMI-8226, A549, and PC-3 cell lines are the most sensitive to all the compounds analyzed.

A raw comparison of the activities of compounds **4–6**, **10**, **11** and **21** with respect to the activity reported for the standard drugs doxorubicin and 5-fluorouracil, used by NCI as control [33], reflects that the activity displayed for these compounds was lower than for the doxorubicin except colon cancer HCT-15 (compounds **4**, **5,** and **21**) and ovarian cancer NCI/ADR-RES (compound **5**). Comparison of the compounds **4–6**, **10**, **11,** and **21** activities with 5-fluorouracil showed that the studied compounds were more active against cell lines of leukemia CCRF-CEM with the exception compound **10**, NSCL cancer HOP-92, and NCI-H226, CNS cancer SNB-75, melanoma SK-MEL-5 with exception compound **11**, ovarian cancer SK-OV-3, and breast cancer T-47D with the exception compounds **6** and **10**. Furthermore, compound **4** also showed the best inhibition of NSCL cancer NCI-H522 cell line; compound **5**—leukemia K-562, NSCL NCI-H522, ovarian cancer OVCAR-3 and OVCAR-4, breast cancer MDA-MB-31/ATCC and HS578T; compound **11**—ovarian cancer OVCAR-4 and breast cancer HS-578T; compound **21**—leukemia K-562 and prostate cancer PC-3 (Table 3). These results suggest that the compounds **4–6**, **10**, **11**, and **21** are promising structures for our future drug development antitumor studies.

#### 2.2.2. CellMiner^TM^ and Gene Enrichment Analysis

Given the promising activity of lead compounds, we performed a preliminary evaluation of their mechanism of action using the CellMiner [34] pattern comparison tool. The premise of this approach is in the assumption that drugs with similar cytotoxic activity profile share molecular target or mechanism of action. Hence, pGI_50_ values obtained for NCI60 cell lines for compounds **4**–**6**, **10**, **11**, and **21** were used as seeds to identify significant (*p* < 0.05) correlations with compounds that were previously tested at NCI. Results were filtered to exclude weak correlations (Pearson’s coefficient *r* < 0.5) and substances with unknown mechanisms of action (Table 5). We also identified correlations between the 60-cell line gene expression patterns and cancer cell lines sensitivity profiles using CellMiner and Gene Ontology (GO) term enrichment analysis to further elucidate plausible molecular effectors and targets of compounds’ action (Supplementary Material Table S1). By analyzing the NCI-60 cell lines for a correlation between their transcriptome and their sensitivity to the cytotoxic effects, we found genes that were significantly correlated (*p* < 0.05) with their in vitro antiproliferative activity. Given the different activity profiles of lead compounds, it is reasonable to discuss them separately.

No analogs have been found for compound **4**, which renders its selectivity towards cancer cell lines as unseen among drugs with known mechanisms of action. Moreover, there was no structural similarity between **4** and the top ten agents with the most similar activity patterns (0.56 < *r* < 0.64, data not shown). Accordingly, no significant correlations in gene expression patterns were identified (Supplementary Material Table S1).

Interestingly, for compound **5** the second best-correlated agent in CellMiner turned out to be CDDO-Im, a well-known anticancer triterpenoid that selectively induces ROS-mediated apoptosis in cancer cells by targeting several key regulatory proteins, especially by activating Keap1-Nrf2-ARE signaling [35]. This finding is supported by gene enrichment analysis (Supplementary Material Table S2 and Figure S49). It was shown that the activity of compound **5** was significantly associated with genes involved in oxidative stress response, namely PRDX1 that encodes peroxiredoxin-1, thiol-specific peroxidase, which catalyzes the reduction of hydrogen peroxide and organic hydroperoxides to protect cancer cell from ROS toxicity. CDDO-Im is known to act via rapid depletion of mitochondrial thiol glutathione that results in the accumulation of ROS [36]. It appears that a similar cellular response mediates cytotoxicity of compound **5**, although precise molecular mechanism is likely to be different since **5** lacks electrophilic enone moieties essential for covalent binding to C151 of Keap1 [37]. Constitutive activation of Keap1-NRF2 enables adaptation to high endogenous ROS levels in the majority of cancer cells, which might explain nearly even GI_50_ values of compound **5** in various cell lines.

The activity of compound **6** correlates the most with calusterone and dromostanolone propionate, anabolic-androgenic steroids used to treat breast cancer. Another strong similarity was found to benzamide histone deacetylase (HDAC) inhibitors and neuroleptic drug fluphenazine. The latter inhibits lymphocyte and myeloma cell proliferation due to anti-serotonergic properties [38]. Calusterone reduces the specific estradiol-receptor interaction [39], and HDAC inhibitors also repress estrogen receptor-dependent signaling [40]. Gene expression pattern of compound **6** strongly suggests the involvement of the immune component (Supplementary Material Table S3 and Figure S50). Many upregulated genes in cell lines sensitive to **6** encode chemokine and interleukin receptors (CX3CR1, IL10RA, IL21R, IL2RG, CCR2, CCR3). TNFRSF18 encodes a receptor for TNFSF18 which is also known as glucocorticoid-induced TNFR-related protein and is considered as a co-stimulatory immune checkpoint molecule. It is involved in the regulation of T cell receptor-mediated cell death [41] and NF-kappa-B activation to regulate blood-cell production [42]. IL-21 is also noted to have anti-tumor effects through continued and increased CD8+ cell response [43]. TNFRSF17 is implicated in leukemia, lymphomas, and multiple myeloma [44,45]. Collectively, these correlations may serve as an explanation of experimentally observed selectivity of compound **6** towards leukemia cells and highlight its potential for the treatment of hematological malignancies.

Compound **10** shares similar patterns of growth inhibition with anaplastic lymphoma kinase inhibitor LDK-378 and dromostanolone propionate. Gene ontology enrichment analysis revealed three main functional categories that were significantly enriched: chromatin organization and assembly, DNA binding and steroid hormone signaling pathways (Supplementary Material Table S3 and Figure S51). The upregulation of PPA2, PPA1, and PRUNE pyrophosphatase genes indicates that **10** might also act through the disruption of mitochondrial membrane potential [46].

Compound **11** shows a moderate correlation with the activity spectrum of PI3K inhibitor wortmannin. It is also associated with the overexpression of genes involved in chromatin remodeling (CHD8, PHF8) and apoptosis (PKN1; see Supplementary Material Table S5 and Figure S52). Possible mechanistic link is in the fact that wortmannin is also implicated in DNA replication, chromatin remodeling, apoptosis [47,48] and acts as autophagy inhibitor [49]. Whether compound **11** inhibits phosphatidylinositol 3-kinase pathway remains to be elucidated.

Two agents have similar activity patterns as compound **21**—dromostanolone propionate and dichloroallyl lawsone. Dromostanolone propionate modulates steroid hormone receptor pathways. It is known that estrogen and growth factor receptor synergy drive malignant progression [50]. Inhibitor of dihydroorotate dehydrogenase dichloroallyl lawsone hampers pyrimidine biosynthesis and depletes the pool of uridine nucleotides necessary for DNA replication [51]. In turn, cytotoxicity of **21** is associated with a wide range of genes involved in nucleic acid metabolism (DDX5, RPUSD4, PUS3), gene transcription (ZNRD1, CTDP1, EIF4A3, CREBZF, HELB), apoptosis (PDCD7, WNT10B), and mitochondrial function (SUPV3L1, ATP5B, NDUFA5, NDUFB10, ATP5A1; see Supplementary Material Table S6 and Figure S53). It appears that compound **21** acts via steroid hormone response elements inducing multiple changes in gene expression profile that ultimately result in cell cycle arrest.

Thus, CellMiner and GO terms gene expression pattern analysis for the most potent cytotoxic compounds suggests that their mechanism of action is associated with induction of oxidative stress and chromatin structure alteration, possibly via interaction with steroid hormone response elements. Noteworthy, several works reported that polyamine analogs disrupt the association of estrogen receptors with co-activators and down-regulation of estrogen response element activity, which results in cancer cell growth inhibition [52,53,54]. Therefore, the development of triterpenic polyamine conjugates opens an interesting possibility to modulate several tumor progression related pathways.

#### 2.2.3. α-Glucosidase Inhibition

Pentacyclic triterpenoids, including lupane type, are a well-known source of potent α-glucosidase inhibitors [55,56,57,58]. Moreover, certain triterpenoids were described to combine cytotoxicity towards cancer cells and α-glucosidase inhibitory properties [59,60]. Glycosylation and cleavage of glycosidic bonds are essential metabolic processes that mediate multiple cellular processes. In particular, neutral glucosidases I and II (NAG) of endoplasmic reticulum are responsible for glycoprotein procession and maturation, and targeting these glucosidases is now being recognized as a possible approach to tackle cancer [61]. Only several types of NAG inhibitors have been identified to date. Iminosugars like N-methyldeoxynojirimycin and catanospermine are among the most studied [62]. It was shown that NAG inhibitors reduce tumor-associated angiogenesis, cell migration, and overall tumor growth [63]. Hence, α-glucosidase inhibition could be considered as an activity adjacent and complementary to direct cytotoxicity towards cancer cells.

Interestingly, squalamine, while not being cytotoxic per se [64], prevents tumor growth and progression due to the inhibition of angiogenesis [13], although the underlying mechanism is believed to be different from glucosidase inhibition [65]. Betulinic acid inhibits yeast α-glucosidase activity and interferes with N-linked glycan modifications to cell-surface intercellular adhesion molecule-1 (ICAM-1) in human lung carcinoma A549 cells in a similar manner to castanospermine, an inhibitor of endoplasmic reticulum α-glucosidases I and II [66]. A previous study showed that adhesion molecules, such as ICAM-1, are associated with metastatic progression [67]. Taken together, these considerations prompted us to evaluate a series of lupanes with amide function or polyamine chain **11**, **12**, **16**, **18**, and **22–26** for α-glucosidase inhibition. Initially, they were screened against α-glucosidase of *S. cerevisiae* as an accessible and high-throughput model. Confirmatory experiments were performed on isolated rough endoplasmic reticulum vesicles from rat liver, which contain neutral α-glucosidases I and II in a membrane-bound form. The results are shown in Table 6.

Among tested compounds, three yeast α-glucosidase inhibitors were identified. Betulonic acid C28-conjugate with triethylenetetramine **11** and C3,C28-bis-aminopropoxy-betulin **18** were found to be the most potent. C2-urea methyl betulonate **25** but not its thiourea analog **26** was found to be active, as well. Other tested compounds proved to be inactive up to 100 µM concentration. Compounds **11** and **18** were also found to inhibit the α-glucosidase activity of rat liver endoplasmic reticulum. Lupanes **22** and **24** that comprise spermidine moiety at C28 via imine bond were even more active. Compound **12** showed only marginal inhibitory properties. Compounds **11** and **18** [19] have not only notable cytotoxic activity, but also inhibit endoplasmic reticulum α-glucosidase. Further studies should be performed to establish structural requirements for NAG inhibition and, moreover, to evaluate its contribution to antitumor activity in animal models that allow taking into account tumor invasiveness and vascularization. We believe that a combination of cytotoxic and α-glucosidase inhibitory properties is an attractive approach for the development of novel anticancer agents that warrants further research.

#### 2.2.4. Antibacterial and Fungicidal Activities

As it was mentioned above, steroidal polyamine conjugate squalamine could be considered as a polyvalent agent with the antiangiogenic and antimicrobial activities [68]. We were interested to know if lupane polyamine derivatives possess antibacterial properties. For this purpose, compounds **2**, **12**, **25,** and **26** were evaluated at the University of Queensland (Australia) using five bacterial strains, including Gram-negative *Escherichia coli, Klebsiella pneumonia, Acinetobacter baumannii,* and *Pseudomonas aeruginosa,* and Gram-positive methicillin-resistant *Staphylococcus aureus* (MRSA). The antifungal activity was determined against Candida albicans and Cryptococcus neoformans. The primary screening of the antimicrobial activity of compounds **2**, **12**, **25,** and **26** were carried out in one concentration of 32 mg/mL in tests of the inhibition of cell reproduction. Samples with inhibition value above 80% were classed as actives. Samples with inhibition values between 50 and 80% were classed as partial actives. It was found that tested compounds **2**, **25,** and **26** did not inhibit the growth of pathogenic microorganisms in the studied concentration, whereas betulonic acid diethylentriamine conjugate **12** showed partial activity against methicillin-resistant *S. aureus* and the fungi *C. neoformans* (Table 7).

## 3. Materials and Methods

### 3.1. Chemistry

#### 3.1.1. General

The spectra were recorded at the Center for the Collective Use “Chemistry” of the Ufa Institute of Chemistry of the UFRC RAS and RCCU “Agidel” of the UFRC RAS. ^1^H and ^13^C-NMR spectra were recorded on a “Bruker AM-500” (Bruker, Billerica, MA, USA, 500 and 125.5 MHz respectively, δ, ppm, Hz) in CDCl_3_, internal standard tetramethylsilane. Mass spectra were obtained on a liquid chromatograph–mass spectrometer LCMS-2010 EV (Shimadzu, Kyoto, Japan). Melting points were detected on a micro table “Rapido PHMK05“ (Nagema, Dresden, Germany). Optical rotations were measured on a polarimeter “Perkin-Elmer 241 MC” (Perkin Elmer, Waltham, MA, USA) in a tube length of 1 dm. Elemental analysis was performed on a Euro EA-3000 CHNS analyzer (Eurovector, Milan, Italy); the main standard is acetanilide. Thin-layer chromatography analyses were performed on Sorbfil plates (Sorbpolimer, Krasnodar, Russia) using the solvent system chloroform–ethyl acetate, 40:1. Substances were detected by 10% H_2_SO_4_ with subsequent heating to 100–120 °C for 2–3 min. Betulonic **1** and 3β,28-diacetoxy-(20*R*)-lupane-29-oic **3** acids were synthesized accordingly [25,69].

#### 3.1.2. Synthesis of 28-(1,4-Diazepan-1-yl)-28-oxolup-20(29)-en-3-one **2**

A solution of betulonic acid **1** (1 mmol, 0.46 g) in anhydrous CHCl_3_ (20 mL) and (COCl)_2_ (3 mmol, 0.26 mL) was stirred at room temperature for 2 h, then concentrated to dryness under reduced pressure. A resulting acid chloride was dissolved in anhydrous CHCl_3_ (30 mL) and treated with homopiperazine (1.5 mmol, 0.15 g) and three drops of Et_3_N. The mixture was stirred at room temperature for 3 h, washed with 5% HCl solution (2 × 100 mL) and H_2_O (100 mL), dried over CaCl_2_, the solvent was removed under reduced pressure. The product was purified by column chromatography with Al_2_O_3_ using CHCl_3_ and a mixture of CHCl_3_–EtOH (100:1) as eluents. Yield 0.41 g (76%); m.p. 145 °С; [α]*_D_*^20^ -6° (с 0.05, CHCl_3_); δ_H_ (500.13 MHz, CDCl_3_) 0.89, 0.94, 0.95, 0.98, 1.03 (5s, 15H, 5CH_3_), 1.13–1.53 (m, 25H, CH and CH_2_), 1.65 (s, 3H, H-30), 1.80–2.5 (m, 6H, CH_2_), 2.80–3.12 (m, 4H, CH_2_), 4.00 (br. s., 1H, NH), 4.55 and 4.69 (both d, ^2^*J* = 2.0 Hz, 2H, H-29); δ_C_ (125.76 MHz, CDCl_3_) 14.64, 15.87, 15.98, 19.64, 19.75, 21.00, 21.68, 25.67, 26.60, 29.33, 29.67, 29.89, 31.38, 32.23, 33.68, 34.14, 36.11, 36.94, 36.99, 39.66, 40.64, 42.03, 45.62, 45.73, 46.76, 47.32, 50.22, 52.62, 52.85, 54.96, 55.08, 109.25 (C-29), 151.13 (C-20), 174.51 (C-28), 218.14 (C-3); Anal. Calcd for C_35_H_56_N_2_O_2_: C, 78.31; H, 10.51; N, 5.22. Found: C, 79.71; H, 10.87; N, 5.32. 

#### 3.1.3. Synthesis of 3β,28-Diacetyloxy-(20*R*)-29-amino-29-oxolupane **4**

A solution of compound **3** (1 mmol, 0.56 g) in anhydrous CHCl_3_ (20 mL) and (COCl)_2_ (3 mmol, 0.26 mL) was stirred at room temperature for 2 h and then concentrated to dryness under reduced pressure. A resulting acid chloride was dissolved in anhydrous CHCl_3_ (30 mL) and treated with liquid NH_3_, then stirred at room temperature for 2 h, washed with 5% HCl solution (2 × 100 mL) and H_2_O (100 mL), dried over CaCl_2_, the solvent was removed under reduced pressure. The product was purified by column chromatography with Al_2_O_3_ using CHCl_3_ as eluent. Yield 0.46 g (83%); m.p. 56 °С; [α]*_D_*^20^ -175° (с 0.05, CHCl_3_); δ_H_ (500.13 MHz, CDCl_3_) δ_H_ (500.13 MHz, CDCl_3_) 0.83, 0.84, 0.85, 0.94, 1.03 (5s, 15H, 5CH_3_), 1.12–1.83 (m, 29H, CH and CH_2_), 2.20 (c, 3H, OCOCH_3_), 2.50 (br. s., 1H, H-15), 3.59 (s, 3H, COOCH_3_), 4.20–4.51 (m, 2H, H-28), 5.55 (br. s., 2H, NH_2_); δ_C_ (125.76 MHz, CDCl_3_) 14.71, 15.96, 16.05, 16.12, 16.50, 17.96, 18.12, 20.89, 21.03, 21.31, 23.06, 23.64, 26.89, 27.74, 27.93, 29.62, 33.85, 34.15, 37.02, 37.77, 38.37, 40.94, 42.61, 42.95, 43.67, 46.44, 49.09, 49.92, 55.30, 62.45 (C-28), 80.86 (C-20), 171.02 (O-C=O), 171.65 (O-C=O), 177.56 (CONH_2_); Anal. Calcd for C_34_H_55_NO_5_: C, 73.21; H, 9.94; N, 2.51. Found: C, 73.40; H, 10.00; N, 2.62. 

### 3.2. Pharmacological Studies

#### 3.2.1. In Vitro Cancer Screen in NCI, USA

The screening is a two-stage process, beginning with the evaluation of all compounds against the 60 cell lines at a single dose of 10^−5^ M. Compounds which exhibit significant growth inhibition are evaluated against the 60-cell panel at five concentration levels. The human tumor cell lines of the cancer-screening panel are grown in RPMI 1640 medium containing 5% fetal bovine serum and 2 mM *l*-glutamine. For a typical screening experiment, cells are inoculated into 96 well micro titer plates in 100 mL at plating densities ranging from 5000 to 40,000 cells/well depending on the doubling time of individual cell lines. After cell inoculation, the micro titer plates are incubated at 37 °C, 5% CO_2_, 95% air and 100% relative humidity for 24 h prior to addition of experimental drugs. After 24 h, two plates of each cell line are fixed in situ with TCA, to represent a measurement of the cell population for each cell line at the time of drug addition (Tz). Experimental drugs are solubilized in dimethylsulfoxide at 400-fold the desired final maximum test concentration and stored frozen prior to use. At the time of drug addition, an aliquot of frozen concentrate is dissolved and diluted to twice the desired final maximum test concentration with complete medium containing 50 mg/mL gentamicin. An additional four, 10-fold, or ½ log serial dilutions are made to provide a total of five drug concentrations plus control. Aliquots of 100 mL of these different drug dilutions are added to the appropriate micro titer wells already containing 100 mL of medium, resulting in the required final drug concentrations. Following drug addition, the plates are incubated for an additional 48 h at 37 °C, 5% CO_2_, 95% air, and 100% relative humidity. For adherent cells, the assay is terminated by the addition of cold TCA. Cells are fixed in situ by the gentle addition of 50 mL of cold 50% TCA (final concentration, 10% TCA) and incubated for 60 min at 4 °C. The supernatant is discarded, and the plates are washed five times with tap water and air dried. Sulforhodamine B (SRB) solution (100 mL) at 0.4% in 1% acetic acid is added to each well, and plates are incubated for 10 min at room temperature. After staining, unbound dye is removed by washing five times with 1% acetic acid and the plates are air dried. Bound stain is subsequently solubilized with 10 mM Trizma base, and the absorbance is read on an automated plate reader at a wavelength of 515 nM. For suspension cells, the methodology is the same except that the assay is terminated by fixing settled cells at the bottom of the wells by gently adding 50 mL of 80% TCA (final concentration, 16% TCA). Using the seven absorbance measurements (time zero (Tz), control growth (C), and test growth in the presence of drug at the five concentration levels (Ti)), the percentage growth is calculated at each of the drug concentrations levels. Percentage growth inhibition is calculated as:

[(Ti_Tz)/(C_Tz)]_100 for concentrations for which Ti_Tz

[(Ti_Tz)/Tz]_100 for concentrations for which Ti < Tz

Three dose response parameters are calculated for each experimental agent. Growth inhibition of 50% (GI_50_) is calculated from [(Ti_Tz)/(C_Tz)]_100 ¼ 50, which is the drug concentration resulting in a 50% reduction in the net protein increase (as measured by SRB staining) in control cells during the drug incubation. The drug concentration resulting in total growth inhibition (TGI) is calculated from (Ti ¼ Tz). The LC_50_ (concentration of drug resulting in a 50% reduction in the measured protein at the end of the drug treatment as compared to that at the beginning) indicating a net loss of cells following treatment is calculated from

[(Ti_Tz)/Tz]_100¼_50

Values are calculated for each of these three parameters if the level of activity is reached; however, if the effect is not reached or is exceeded, the value for that parameter is expressed as greater or less than the maximum or minimum concentration tested [29,30,31].

#### 3.2.2. CellMiner and Gene Ontology Enrichment Analysis

Analysis of the GI_50_ data from the NCI60 cell line screening for the compounds **4–6, 10, 11**, and **21** was performed using pattern comparison functionality of CellMiner (http://discover.nci.nih.gov/cellminer/) [34]. GO term enrichment analysis and functional profiling of genes with expression profiles that were significantly correlated (*p* < 0.5) with the test compounds’ cytotoxicity profile across the NCI-60 panel of cancer cell lines were performed with a Cytoscape 3.8.0 (Institute for Systems Biology, Seattle, WA, USA) and BiNGO 3.0.3 plugin (Ghent University, Ghent, Belgium) [70]. Significantly enriched biological processes and cellular components were evaluated using a hypergeometric test corrected for multiple hypothesis testing (*p* < 0.05) using a Benjamini–Hochberg false discovery rate (FDR) correction.

#### 3.2.3. Yeast α-Glucosidase Activity Assay

In a 96-well flat transparent plate, the 0.12 U/mL enzyme solution (EC 3.2.1.20, expressed in *S. cerevisiae*, Sigma #G0660, St. Louis, MO, USA) was incubated with test compounds in 67 mM PBS (pH 6.8) at 37 °C for 5 min. Then, *p*-nitrophenyl-α-*d*-glucopyranoside (Sigma #N1377, St. Louis, MO, USA) was added to 1 mM final concentration and an increase in absorbance was recorded for 15 min at 405 nm wavelength with Infinite M200 PRO microplate reader (Tecan, Austria). Test compounds were dissolved in DMSO and diluted to the desired concentration in 67 mM PBS (pH 6.8) supplemented with 0.01% Tween 80 to avoid aggregation. Final DMSO concentration was <0.25% in sample wells, and it was also introduced in control and blank wells. Acarbose was used as a positive control. Due to the absence of normal distribution of data, a Mann–Whitney U-test was used to determine statistical significance versus enzyme control using GraphPad Prism 8.0.1.

#### 3.2.4. Endoplasmic Reticulum α-Glucosidase Activity Assay

Endoplasmic reticulum was isolated from hepatocytes of decapitated male Sprague-Dawley rats, which were starved overnight and sacrificed the next morning. All experiments were approved by Volgograd regional ethic committee. The whole procedure was performed at 4 °C. In brief, a fresh tissue sample from an animal was washed twice with 10 mL of PBS to remove residual blood. The tissue was placed on a paper towel in order to absorb excess liquid and blood clots, if present. It was cut further into small pieces (1.5–2 cm) and washed once more. After blotting, the tissue was cut into smaller slices (0.3–0.5 cm) and homogenized in isotonic extraction buffer (50 mM HEPES, pH 7.8, with 1.25 M sucrose, 5 mM EGTA, and 125 mM NaCl supplemented with 1% *v/v* protease inhibitor cocktail for mammalian cells, Sigma #P8340, St. Louis, MO, USA) with UltraTurrax T10 (IKA, Germany). Homogenate was centrifuged at 1000× *g* for 10 min at 4 °C. After removal of the floating lipid layer by aspiration, supernatant was separated and centrifuged at 12,000× *g* for 15 min. at 4 °C. The supernatant fraction, which is the post mitochondrial fraction (PMF), was used for isolation of rough endoplasmic reticulum (RER) enriched microsomes by precipitation with 8 mM CaCl_2_ added dropwise. The final concentration of CaCl_2_ was 7 mM. Resulting sample was centrifuged at 8000 g for 10 min. at 4 °C. The supernatant was discarded, and the pellet was suspended in isotonic extraction buffer (0.3 mL of buffer for each g of original tissue). The suspension was homogenized completely to afford RER enriched microsomes. In a 96-well flat transparent plate 25 µL of the isolated RER microsomes were incubated with 50 µL of test compounds in 67 mM PBS (pH 6.8) at 37 °C for 10 min. Then, *p*-nitrophenyl-α-*d*-glucopyranoside (Sigma #N1377, St. Louis, MO, USA) was added to 1 mM final concentration and increase in absorbance was recorded for 42 min at 405 nm wavelength with Infinite M200 PRO microplate reader (Tecan, Austria). Concentration of RER microsomes was adjusted to achieve an increase in optical density of ~0.2 in control samples after 30 min. of incubation. Test compounds were prepared as described above for the yeast α-glucosidase assay.

#### 3.2.5. Antibacterial and Antifungal Assays

Samples were prepared in DMSO and water to a final testing concentration of 32 µg/mL, in 384-well, non-binding surface plate (NBS) for each bacterial/fungal strain, and in duplicate (*n* = 2) and keeping the final DMSO concentration to a maximum of 1% DMSO. All the sample-preparation was done using liquid handling robots. Compounds that showed solubility issues during stock solution preparation are detailed in the datasheet.

##### Antibacterial Assay

All bacteria were cultured in cation-adjusted Mueller Hinton broth (CAMHB) at 37 ^°^C overnight. A sample of each culture was then diluted 40-fold in fresh broth and incubated at 37 °C for 1.5–3 h. The resultant mid-log phase cultures were diluted (CFU/ ml measured by OD_600_), then added to each well of the compound containing plates, giving a cell density of 5 × 10^5^ CFU/mL and a total volume of 50 µl. All the plates were covered and incubated at 37 °C for 18 h without shaking.

Inhibition of bacterial growth was determined measuring absorbance at 600 nm (OD_600_), using a Tecan M1000 Pro monochromator plate reader. The percentage of growth inhibition was calculated for each well, using negative control (media only) and positive control (bacteria without inhibitors) on the same plate as references. The significance of the inhibition values was determined by modified Z-scores, calculated using the median and MAD of the samples (no controls) on the same plate. Samples with inhibition value above 80% and Z-score above 2.5 for either replicate were classed as actives. Samples with inhibition values between 50% and 80% and Z-score above 2.5 for either replicate were classed as partial actives.

The percentage of growth inhibition was calculated for each well, using negative control (media only) and positive control (bacteria without inhibitors) on the same plate. The MIC was determined as the lowest concentration at which the growth was fully inhibited, defined by inhibition of ≥ 80%. The maximal percentage of growth inhibition is reported as D_Max_, indicating any compounds with partial activity. Hits were classified by MIC ≤ 16 μg/mL in either replicate.

##### Antifungal Assay

Fungi strains were cultured for 3 days on Yeast Extract-Peptone Dextrose (YPD) agar at 30 °C. A yeast suspension of 1 × 10^6^ to 5 × 10^6^ CFU/mL (as determined by OD_530_) was prepared from five colonies. The suspension was subsequently diluted and added to each well of the compound-containing plates giving a final cell density of fungi suspension of 2.5 × 10^3^ CFU/mL and a total volume of 50 µl. All plates were covered and incubated at 35 ^°^C for 24 h without shaking.

Growth inhibition of *C. albicans* was determined measuring absorbance at 530 nm (OD_530_), while the growth inhibition of *C. neoformans* was determined measuring the difference in absorbance between 600 and 570 nm (OD_600–570_), after the addition of resazurin (0.001% final concentration) and incubation at 35 °C for an additional 2 h. The absorbance was measured using a Biotek Synergy HTX plate reader. The percentage of growth inhibition was calculated for each well, using negative control (media only) and positive control (fungi without inhibitors) on the same plate. The significance of the inhibition values was determined by modified Z-scores, calculated using the median and MAD of the samples (no controls) on the same plate. Samples with an inhibition value above 80% and Z-score above 2.5 for either replicate (*n* = 2 on different plates) were classed as actives. Samples with inhibition values between 50% and 80% and a Z-score above 2.5 for either replicate were classed as partial actives.

In both cases, the percentage of growth inhibition was calculated for each well, using negative control (media only) and positive control (fungi without inhibitors) on the same plate. The MIC was determined as the lowest concentration at which the growth was fully inhibited, defined by inhibition of ≥80% for *C. albicans* and an inhibition ≥70% for *C. neoformans.* Due to a higher variance in growth and inhibition, a lower threshold was applied to the data for *C. neoformans*. The maximal percentage of growth inhibition is reported as D_Max_, indicating any compounds with marginal activity. Hits were classified by MIC ≤ 16 μg/mL in either replicate.

## 4. Conclusions

In general, these results show that triterpenic polyamines being analogs of steroidal squalamine and trodusquemine are important substances for the search of new drugs with anticancer, antidiabetic and antimicrobial activities. We have found that lupane type C28 and C29-carboxamides, C28-conjugates with triethylenetetramine, and branched C3-cyanoethylated polyamine possess high cytotoxic activity against NCI-60 cancer cell line panel with GI_50_ range from 1.09 to 54.40 μM along with good water solubility up to 100 µM. CellMinner pattern comparison analysis suggests that lead compounds exert multi-target antiproliferative activity associated with oxidative stress induction and chromatin structure alteration. Betulonic acid C28-conjugate with triethylenetetramine and C3,C28-bis-aminopropoxy-betulin were also potent α-glucosidase inhibitors. The betulonic acid diethylentriamine conjugate showed partial activity against methicillin-resistant *S. aureus* and the fungi *C. neoformans*. The study of possible mechanisms of action for these compounds will be the task for our further investigations.

## Figures and Tables

**Table 1:** In vitro anticancer activity in 60 human tumor cell lines for compounds **2**, **7–9**, **12–20**, **22** and **24–26** at 10 µM.

Compounds	Mean Growth, %	Range of Growth, %	Sensitive Cell Lines, %
**2**	89.47	8.04 to 126.62	Leukemia: SR (8.04)
**7**	99.22	53.80 to 146.79	not active
**8**	80.54	20.45 to 135.21	Colon Cancer: HT29 (24.32); Breast Cancer: MDA-MB-231/ATCC (20.45)
**9**	72.02	25.88 to 115.11	Leukemia: HL-60(TB) (25.88)
**12**	98.94	59.08 to 140.73	not active
**13**	104.76	85.83 to 118.03	not active
**14**	36.65	‒33.78 to 73.79	Leukemia: CCRF-CEM (14.95), HL-60(TB) (−14.34), MOLT-4 (11.67), RPMI-8226 (22.20); Non-Small Cell Lung Cancer: HOP-92 (8.07), NCI-460 (20.60); Colon Cancer: HCT-116 (17.53), HT29 (23.68), SW-620 (28.83); CNS Cancer: SF-295 (31.97), SNB-75 (23.86), U251 (27.93); Melanoma: SK-MEL-5 (−8.44); Renal Cancer: ACHN (23.51), CAKI-1 (18.57), UO-31 (17.41); Prostate Cancer: PC-3 (19.12); Breast Cancer: MCF7 (22.21), MDA-MB-231/ATCC (−33.78), T-47D (28.02)
**15**	90.11	56.79 to 114.03	not active
**16**	100.79	59.32 to 140.95	not active
**17**	98.74	79.25 to 111.36	not active
**19**	63.02	−39.45 to 140.99	Leukemia: CCRF-CEM (11.76), K-562 (1.52), MOLT-4 (10.06), RPMI-8226 (29.30), SR (18.41); Non-Small Cell Lung Cancer: NCI-460 (−16.23); Colon Cancer: COLO 205 (18.88), HCT-116 (12.60), HT29 (4.21), KM12 (31.48), SW-620 (7.33); CNS Cancer: U251 (−39.45); Melanoma: LOX IMVI (5.71); Ovarian cancer: IGROV1 (27.33), OVCAR-8 (−28.27); Breast Cancer: MCF7 (14.49)
**20**	100.53	87.76 to 111.05	not active
**22**	101.76	80.54 to 123.09	not active
**24**	93.62	21.69 to 131.79	Leukemia: SR (21.69)
**25**	91.74	21.22 to 112.08	Leukemia: SR (21.22)
**26**	90.51	60.92 to 125.91	not active

**Table 2:** Percentage cell growth of sixty human tumor cell line anticancer screening data of the tested compounds **4–6**, **10**, **11**, **21** at single dose assay (10 µM concentration)^a^.

Subpanel Tumor Cell Lines	Percentage Cell Growth for Compounds					
	4	5	6	10	11	21
**Leukemia**						
CCRF-CEM	−12.36	3.11	5.18	1.39	−11.29	7.42
HL-60(TB)	−23.06	1.17	−23.98	1.25	−35.58	0.90
K-562	3.10	4.57	−15.78	1.10	−16.99	5.42
MOLT-4	−29.06	1.86	1.18	2.76	−16.31	−26.81
RPMI-8226	−18.26	−19.77	−8.15	−6.66	−30.74	3.28
SR	10.81	15.50	−4.72	23.66	4.96	1.86
**NSC Lung Cancer**						
A549/ATCC	9.89	−69.33	−64.63	−4.70	−76.50	15.82
EKVX	−25.21	−10.31	−11.36	−15.68	11.37	53.13
HOP-62	−72.98	−72.18	−69.12	−26.80	43.09	32.54
HOP−92	−83.15	−62.83	−65.35	−55.49	−65.19	−14.95
NCI-H226	−58.51	−50.47	−23.56	36.64	74.30	2.42
NCI-H23	−40.35	−27.08	−51.70	−8.47	−35.23	21.79
NCI-H322M	−78.19	−58.91	−83.84	−68.83	−81.08	46.12
NCI-H460	−61.81	−37.45	−100.00	−43.19	−90.93	4.23
NCI-H522	−61.13	−53.76	−57.83	−16.98	−69.70	13.36
**Colon Cancer**						
COLO 205	−52.98	−66.21	−19.47	7.82	−47.48	17.60
HCC-2998	−68.10	−39.02	−90.36	−23.40	−90.04	26.92
HCT-116	−84.18	−24.80	−26.53	1.97	−80.70	4.16
HCT-15	−44.10	6.36	−24.81	−22.06	−36.56	34.28
HT29	−77.68	−49.47	−39.56	3.90	−77.42	3.88
KM12	−62.05	−63.59	−52.23	−25.31	−81.93	10.89
SW-620	−55.13	−4.49	−86.66	−36.18	−76.71	21.96
**CNS Cancer**						
SF-268	−61.04	4.41	−30.99	−16.88	−38.68	29.14
SF-295	−50.75	−57.70	−84.72	−26.39	−54.47	36.56
SF-539	−83.91	−70.46	−100.00	−36.20	−92.71	−2.29
SNB-19	−39.95	1.30	−74.52	−29.49	−78.54	28.93
SNB-75	−47.83	−39.09	−79.32	−51.56	−41.55	18.44
U251	−51.40	−69.32	−71.65	−13.77	−81.82	−39.66
**Melanoma**						
LOX IMVI	−85.52	−72.80	−41.03	1.37	−68.23	14.56
MALME-3M	−88.97	−57.05	−100.00	−18.76	−72.82	25.27
M14	−44.25	8.80	−30.98	5.07	−62.82	26.57
MDA-MB-435	−63.78	−4.44	−89.21	−32.41	−49.89	14.55
SK-MEL-2	−77.63	−31.18	−65.39	−24.21	56.36	36.23
SK-MEL-28	−73.19	0.38	−98.68	−31.42	−73.91	29.25
SK-MEL-5	−71.94	−39.59	−90.10	−23.93	−86.18	−84.68
UACC-257	−46.24	−6.08	−39.58	−46.67	−10.66	8.82
UACC-62	−82.97	−23.23	−100.00	−22.62	−77.94	−25.76
**Ovarian Cancer**						
IGROV1	−71.25	−58.08	−81.43	−6.94	−84.81	18.78
OVCAR−3	−76.19	−46.59	−66.02	−45.89	−72.48	−34.55
OVCAR-5	−77.37	−21.57	−83.37	−47.68	−44.11	50.59
OVCAR-8	−31.70	−64.82	−12.90	−3.81	−75.03	13.17
NCI/ADR-RES	−57.29	−8.91	−13.05	−9.52	−73.33	17.06
SK-OV-3	−11.89	−45.72	−14.17	−27.22	70.49	34.60
**Renal Cancer**						
786-0	−73.99	−72.16	−83.51	−22.61	−84.46	−11.47
A498	−81.70	−28.33	−32.64	−44.82	80.81	−0.38
ACHN	−75.68	−80.29	−100.00	−49.24	−83.29	13.94
CAKI-1	−45.80	−64.30	−93.59	−54.52	−8.79	18.52
RXF 393	−64.94	−49.35	−82.88	−42.28	−71.88	−43.14
SN12C	−91.61	−45.76	−53.68	−34.74	−88.48	−71.91
TK-10	−35.40	−69.84	−67.30	−32.23	−77.68	37.88
UO-31	−97.54	−100.00	−100.00	−34.32	−96.35	19.03
**Prostate Cancer**						
PC-3	−47.42	−24.47	1.07	−53.78	−24.64	4.49
DU-145	−69.87	−32.96	−8.34	−100.00	−56.67	27.53
**Breast Cancer**						
MCF7	−45.58	−12.52	−60.54	−8.46	−57.97	12.44
MDA-MB-231/ATCC	−88.58	−28.40	−56.94	−47.78	−92.38	5.09
HS 578T	−32.99	−1.70	−23.26	−4.98	47.76	24.26
BT-549	−55.13	−21.60	−51.94	−2.14	58.31	4.40
T-47D	−8.82	−24.75	−32.56	−12.76	−4.36	15.11
MDA-MB-468	−40.40	−56.03	−48.60	−19.87	−49.06	4.41

^a^ Survival of cells cultivated in the presence of 10 µM of compound under examination (in percent) compared with control cells (without the addition of compound to the culture medium) is given. Negative values correspond to cell death.

**Table 3:** In vitro cytotoxic effects of compounds **4–6**, **10**, **11** and **21** and standard drugs doxorubicin (DRB) and 5-fluorouracil (5-FU) against NCI’s human tumor cell line screen.

Panel/Cell Line	4	5	6	10	11	21	DRB	5-FU
	GI_50_^a^ (µM)	GI_50_ (µM)	GI_50_ (µM)	GI_50_ (µM)	GI_50_ (µM)	GI_50_ (µM)	GI_50_ (µM)	GI_50_ (µM)
**Leukemia**								
CCRF-CEM	5.19	4.49	4.55	22.1	9.65	11.7	0.08	9.97
HL-60(TB)	2.76	3.59	7.33	16.5	8.88	5.18	0.19	2.30
K-562	4.09	3.04	6.90	9.84	8.20	2.23	-	3.58
MOLT-4	3.29	3.43	11.5	12.9	6.96	2.52	0.03	0.35
RPMI-8226	2.63	3.53	2.81	17.2	6.78	4.91	0.08	0.04
SR	3.32	4.14	6.76	9.05	5.91	2.87	0.03	-
**Non-Small Cell Lung Cancer**								
A549/ATCC	3.97	2.11	4.97	19.6	7.69	5.27	0.06	0.18
EKVX	9.28	2.96	10.5	23.3	7.34	4.77	0.41	-
HOP-62	1.10	3.21	16.5	18.3	7.91	12.5	0.07	0.39
HOP-92	5.21	2.69	4.61	10.8	2.93	6.90	0.10	77.9
NCI-H226	7.28	4.11	15.5	21.2	7.74	5.43	0.05	54.7
NCI-H23	9.42	2.26	14.7	22.6	9.14	7.59	0.15	0.33
NCI-H322M	13.0	2.59	15.1	17.6	7.32	>50.0	-	-
NCI-H460	13.4	2.12	14.7	14.0	9.40	11.1	0.02	0.05
NCI-H522	4.94	3.13	10.3	21.0	8.72	8.34	0.03	7.27
**Colon Cancer**								
COLO 205	4.31	3.63	15.9	21.0	8.74	9.02	0.18	0.15
HCC-2998	10.4	3.96	14.1	21.4	7.16	13.8	0.26	0.05
HCT-116	3.57	1.97	4.40	13.9	6.65	2.89	0.08	0.22
HCT-15	4.04	3.28	11.5	21.7	7.65	5.77	6.46	0.11
HT29	3.73	2.56	14.6	6.95	7.17	6.70	0.12	0.17
KM12	3.86	3.20	6.81	17.3	8.47	9.35	0.27	0.21
SW-620	7.70	3.88	14.7	22.5	7.91	4.45	0.09	0.92
**CNS Cancer**								
SF-268	6.95	4.34	13.5	23.0	7.53	11.4	0.10	1.62
SF-295	8.57	3.39	13.8	22.6	9.42	21.4	0.10	-
SF-539	5.52	1.78	11.1	17.9	7.53	11.0	0.12	0.06
SNB-19	11.9	4.57	12.9	24.7	8.50	12.6	0.04	3.81
SNB-75	5.59	2.74	11.1	32.1	6.26	31.2	0.07	78.7
U251	5.10	2.54	12.7	17.7	7.34	4.30	0.04	0.92
**Melanoma**								
LOX IMVI	10.2	1.77	14.9	25.5	8.72	4.32	0.07	0.24
MALME-3M	10.5	2.39	14.6	23.3	7.40	11.8	0.12	0.05
M14	7.38	6.05	11.0	24.7	8.38	11.9	0.18	0.98
MDA-MB-435	5.23	8.65	13.4	28.7	8.70	9.70	0.25	0.07
SK-MEL-2	10.8	7.63	16.8	27.3	>50.0	13.8	0.17	56.7
SK-MEL-28	12.0	1.09	16.0	22.1	8.49	17.1	0.21	1.03
SK-MEL-5	3.80	5.22	11.1	19.4	6.98	3.02	0.08	0.46
UACC-257	4.83	13.2	15.1	20.6	7.91	5.35	0.14	3.55
UACC-62	5.46	4.20	9.29	27.4	8.96	2.32	0.12	0.52
**Ovarian Cancer**								
IGROV1	13.2	3.32	16.1	21.5	6.42	14.9	0.17	1.22
OVCAR-3	3.95	2.07	12.2	15.7	7.65	5.17	0.39	0.01
OVCAR-4	4.72	3.54	14.1	25.6	7.39	12.3	0.37	4.43
OVCAR-5	14.4	3.78	18.6	14.8	8.78	16.7	0.41	10.9
OVCAR-8	54.4	3.12	18.8	27.7	8.41	9.66	0.10	1.74
NCI/ADR-RES	7.74	3.17	18.9	24.8	8.41	13.5	7.16	0.31
SK-OV-3	13.4	8.18	17.5	17.7	10.6	10.8	0.22	21.8
**Renal Cancer**								
786-0	10.9	2.03	12.4	23.2	7.80	6.42	0.13	0.72
A498	12.8	12.2	19.6	22.9	42.6	21.7	0.10	0.35
ACHN	5.78	2.44	12.0	21.7	13.8	10.5	0.08	0.27
CAKI-1	3.24	1.98	5.92	21.9	5.87	10.2	0.95	0.07
RXF 393	4.28	2.10	13.2	17.1	5.60	5.64	0.10	2.61
SN12C	5.65	3.00	13.5	20.8	7.56	7.28	0.07	0.49
TK-10	14.7	2.79	15.8	23.9	10.1	13.8	-	1.12
UO-31	7.57	1.72	11.7	38.9	6.85	15.6	0.49	1.42
**Prostate Cancer**								
PC-3	2.67	2.83	2.95	12.6	6.26	2.26	0.32	2.36
DU-145	9.94	3.21	14.1	17.5	7.47	13.4	0.11	0.36
**Breast Cancer**								
MCF7	3.27	2.91	13.2	10.7	7.38	3.67	0.03	0.07
MDA-MB-31/ATCC	11.8	1.97	15.1	21.1	8.03	10.3	0.51	6.60
HS 578T	12.4	5.74	20.1	40.5	8.30	30.2	0.33	9.77
BT-549	8.17	3.99	15.0	23.8	8.91	11.3	0.23	10.6
T-47D	2.91	4.55	10.9	13.1	7.52	3.47	0.06	8.12
MDA-MB-468	3.96	3.20	8.88	12.6	8.09	2.02	0.05	-

^a^ GI_50_ was the drug concentration resulting in a 50% reduction in the net protein increase (as measured by SRB staining) in control cells during the drug incubation, determined at five concentration levels (100, 10, 1.0, 0.1 and 0.01 µM). LC_50_ is a parameter of cytotoxicity and reflects the molar concentration needed to kill 50% of the cells. The symbol “-” designates the absence of data.

**Table 4:** Selectivity ratio of the compounds **4–6**, **10**, **11** and **21** towards NCI nine tumor subpanels.

Panel	Compound 4			Compound 5			Compound 6		
	MID^a^	MID^b^	Selectivity^c^	MID^a^	MID^b^	Selectivity^c^	MID^a^	MID^b^	Selectivity^c^
Leukemia	7.87	3.55	2.22	3.67	3.20	1.15	12.15	6.64	1.83
NSCL cancer		7.50	1.05		2.79	1.32		11.88	1.02
Colon Cancer		5.37	1.47		3.21	1.14		11.72	1.04
CNS cancer		7.27	1.08		3.23	1.14		12.52	0.97
Melanoma		7.80	1.01		5.58	0.66		13.58	0.89
Ovarian Cancer		15.97	0.49		3.88	0.95		14.10	0.86
Renal Cancer		8.12	0.97		3.53	1.04		13.02	0.93
Prostate cancer		6.31	1.25		3.02	1.22		8.53	1.42
Breast cancer		7.09	1.11		3.73	0.98		13.86	0.88
**Panel**	**Compound 10**			**Compound 11**			**Compound 21**		
	**MID^a^**	**MID^b^**	**Selectivity^c^**	**MID^a^**	**MID^b^**	**Selectivity^c^**	**MID^a^**	**MID^b^**	**Selectivity^c^**
Leukemia	20.46	14.59	1.40	9.17	7.73	1.19	10.25	4.90	2.09
NSCL cancer		18.71	1.09		7.58	1.21		12.43	0.83
Colon Cancer		17.82	1.15		7.68	1.19		7.42	1.38
CNS cancer		23.00	0.89		7.76	1.18		15.32	0.67
Melanoma		24.33	0.84		12.84	0.71		8.81	1.16
Ovarian Cancer		24.63	0.83		8.24	1.11		11.86	0.86
Renal Cancer		23.80	0.86		12.52	0.73		11.39	0.89
Prostate cancer		15.05	1.36		6.87	1.33		7.83	1.31
Breast cancer		20.30	1.01		8.04	1.14		10.16	1.00

MID^a^—Average sensitivity of all cell line in μM; MID^b^—Average sensitivity of all cell line of particular subpanel in μM; Selectivity^c^—ratio MID^a^: MID^b^.

**Table 5:** Possible mechanism of action for the lead compounds according to CellMiner^a^.

Compounds	Pearson’s Correlation ^b^	P Value	NSC ^c^	Name	Mechanism of Action	FDA Status
**4**	-	-	-	-	-	-
**5**	0.545	0.000024	727038	CDDO-Im	Nrf2	-
**6**	0.672	0	88536	Calusterone	Hormone	FDA approved
	0.65	0	12198	Dromostanolone propionate	Hormone	FDA approved
	0.62	0	734945	*N*-(4-Aminophenyl)-4-(3-(3,4-dihydroisoquinolin-2(1*H*)-yl)prop-1-en-2-yl)benzamide	HDAC	-
	0.537	0.000012	736101	4-(3-(10*H*-Phenothiazin-10-yl)prop-1-en-2-yl)-*N*-(2-aminophenyl)benzamide	HDAC	-
	0.565	0.000024	730001	*N*-(2-Aminophenyl)-4-(3-(3,4-dihydro-1*H*-pyrido[3,4-b]indol-2(9*H*)-yl)prop-1-en-2-yl)benzamide	HDAC	-
	0.507	0.000041	92339	Fluphenazine	Antipsychotic	FDA approved
	0.517	0.000063	734949	4-[3-(3,4-Dihydro-1*H*-isoquinolin-2-yl)prop-1-en-2-yl]-*N*-pyrazin-2-ylbenzamide	HDAC	-
**10**	0.563	0.000004	776422	LDK-378	ALK inhibitor	FDA approved
	0.537	0.000014	777193	LDK-378	ALK inhibitor	FDA approved
	0.511	0.000035	12198	Dromostanolone propionate	Hormone	FDA approved
**11**	0.507	0.000379	221019	Wortmannin	PI3K inhibitor	Clinical trial
**21**	0.578	0.000002	12198	Dromostanolone propionate	Hormone	FDA approved
	0.537	0.000014	126771	Dichloroallyl lawsone	DNA/RNA synthesis inhibitor	-

^a^ The drug activity levels used were expressed as pGI_50_ and obtained from the Developmental Therapeutics Program (DTP) at http://dtp.cancer.gov/index.html. ^b^ Pearson’s correlations between the compound and NCI synthetic library, only correlations with *r* > 0.5 were considered. ^c^ National Service Center Number assigned by the Developmental Therapeutics Program (NCI) for compounds tested in the NCI-60 assay. The symbol “-” designates the absence of data.

**Table 6:** Inhibition of α-glucosidases by the compounds **11**, **12**, **16**, **18** and **22–26.**

Compounds	Yeast α-Glucosidase, IC_50_ ± SE (μM)	Rat Liver ER Neutral α-Glucosidase Inhibition, m ± SD (%)	
		100 μM	10 μM
**11**	4.84 ± 1.02	43.60 ± 7.98 *	15.03 ± 8.98
**12**	>100	23.59 ± 1.34 *	10.21 ± 3.49
**16**	>100	14.94 ± 6.31	9.05 ± 6.68
**18**	5.70 ± 1.09	36.51 ± 14.90 *	56.64 ± 4.24 *
**22**	>100	58.52 ± 8.21 *	31.42 ± 7.96 *
**23**	>100	5.85 ± 8.03	−1.34 ± 2.22
**24**	>100	54.62 ± 15.38 *	26.96 ± 2.33 *
**25**	53.3 ± 5.7	9.62 ± 6.94	8.76 ± 9.63
**26**	>100	26.60 ± 13.03	11.63 ± 9.09
**Acarbose**	436.7 ± 10.2	74.55 ± 3.76 * (1 mM) 24.09 ± 6.48 * (100 μM)	5.68 ± 4.68

* *p* < 0.05 vs. enzyme control (Mann-Whitney U-test, *n* = 3).

**Table 7:** % Growth inhibition of compound **2**, **12**, **25** and **26** at concentration 32 µg/mL.

Compounds	Gram-Positive Bacteria		Gram-Negative Bacteria			Fungi	
	*S. aureus*	*E. coli*	*K. pneumonia*	*P. aeruginosa*	*A. baumannii*	*C. albicans*	*C. neoformans*
	Strain ATCC 43300	Strain ATCC 25922	Strain ATCC 700603	Strain 19606	Strain ATCC 27853	Strain ATCC 90028	Strain H99, ATCC 208821
**2**	40.30	1.96	0.98	8.34	33.07	4.14	−5.90
**12**	71.80	−9.57	9.20	7.72	19.49	11.74	62.56
**25**	19.78	−5.34	2.44	−8.92	40.86	13.97	−7.93
**26**	12.66	−7.03	−3.89	−4.64	28.24	5.83	−13.04

## Supplementary Materials

The Supplementary Materials are available online. Figures S1–S2: ^1^H and ^13^C spectra for compounds **2** and **4**, Figures S3–S30: Anticancer screening data of compounds **4–6**, **10**, **11** and **21**, Table S1–S6: Gene ontology (GO) term enrichment analysis for compounds **4–6**, **10**, **11** and **21**, Figures S31–S35: Network visualization of GO for compounds **5**, **6**, **10**, **11** and **21**.
