# Supplementary material for: Evaluation of Cytotoxicity and α-Glucosidase Inhibitory Activity of Amide and Polyamino-Derivatives of Lupane Triterpenoids
Source: Molecules. 2020 Oct 20;25(20):4833. doi: 10.3390/molecules25204833 (PMC7587962; doi:10.3390/molecules25204833)

molecules

Supplementary Materials: Evaluation of cytotoxicity and α-glucosidase inhibitory activity of amide and polyamino-derivatives of lupane triterpenoids

Oxana B. Kazakova[1]*, Gul'nara V. Giniyatullina[1], Akhat G. Mustafin[1], Denis A. Babkov[2], Elena V. Sokolova[2], Alexander A. Spasov[2]*

[1]*Ufa Institute of Chemistry of the Ufa Federal Research Centre of the Russian Academy of Sciences, 71, pr. Oktyabrya, 450054 Ufa, Russian Federation*
[2]*Scientific Center for Innovative Drugs, Volgograd State Medical University, Novorossiyskaya st. 39, Volgograd 400087, Russian Federation*

Correspondence
Prof. Dr. Oxana B. Kazakova
Ufa Institute of Chemistry of the
Ufa Federal Research Centre of the Russian Academy of Sciences
71 Prospect Oktyabrya
Ufa, 450054 Russian Federation
E-mail: obf@anrb.ru

Prof. Dr. Alexander A. Spasov
Scientific Center for Innovative Drugs of the
Volgograd State Medical University
39 Novorossiyskaya st.
Volgograd, 400087 Russian Federation
E-mail: aspasov@mail.ru

Figure S1. ^{1}H and ^{13}C of compound **2**.

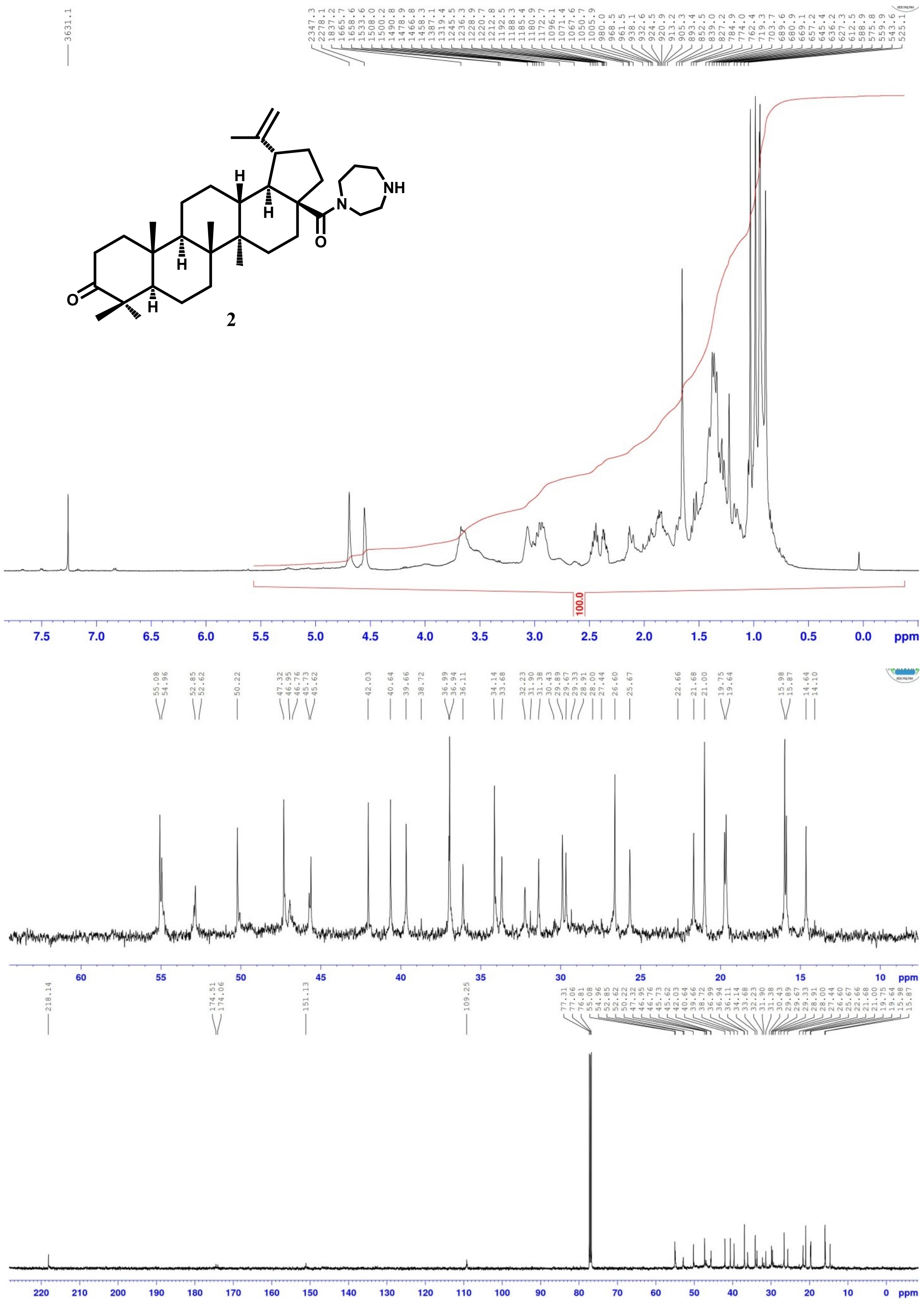

Figure S2. ^{1}H and ^{13}C of compound **4**.

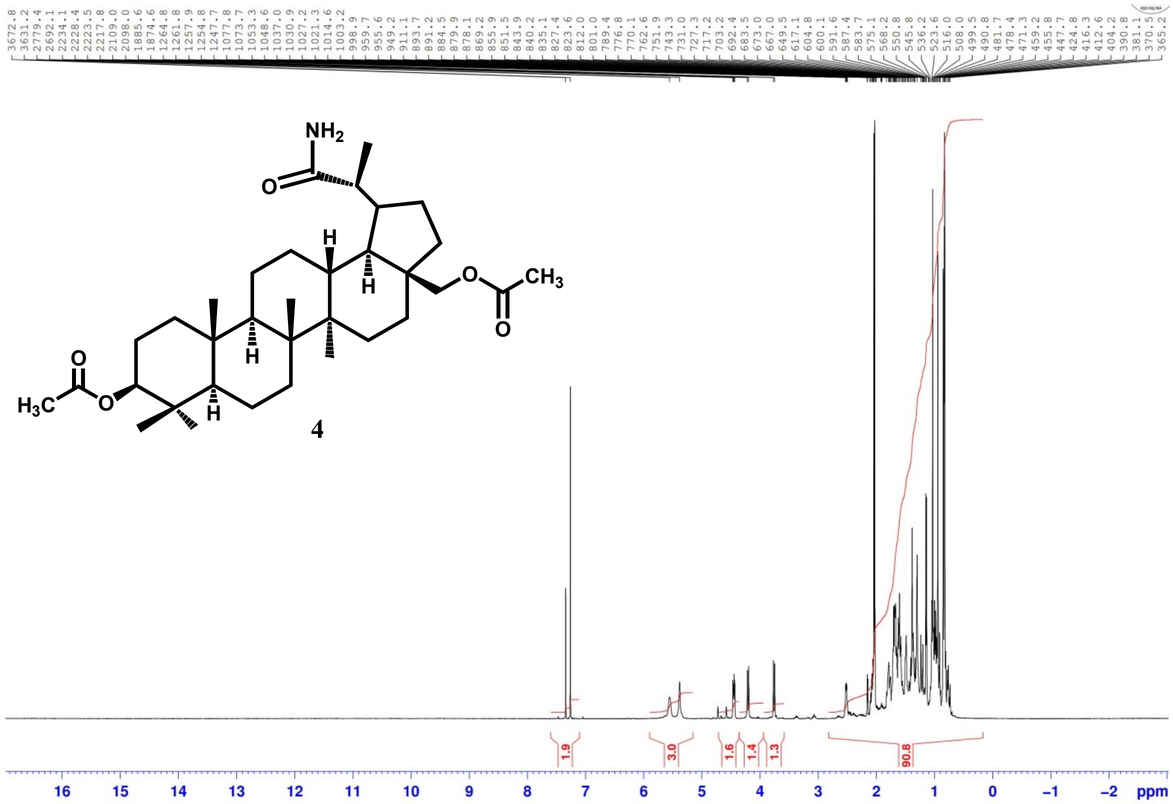

4

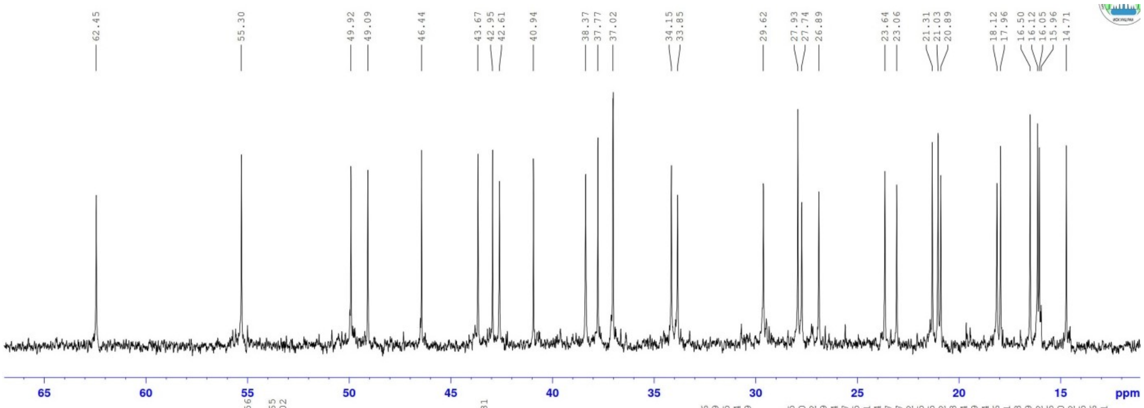

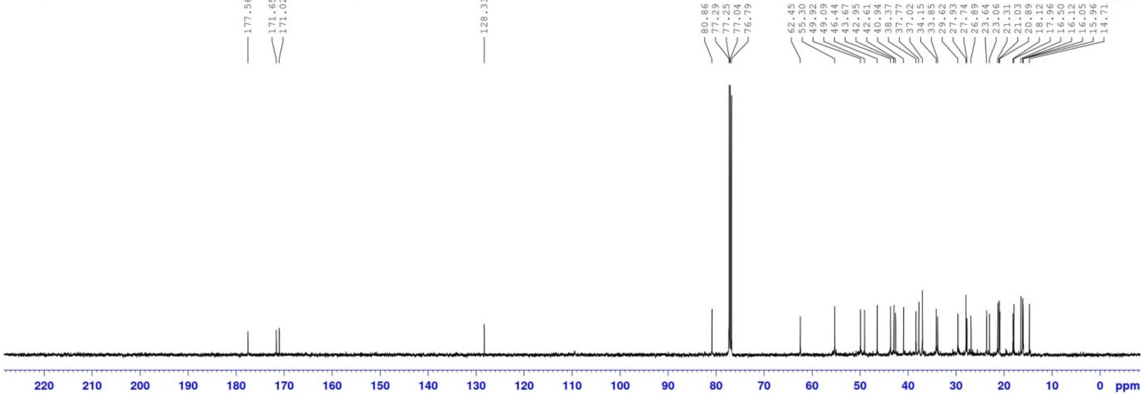

Figure S3. Anticancer screening data of compound **2** at single dose assay

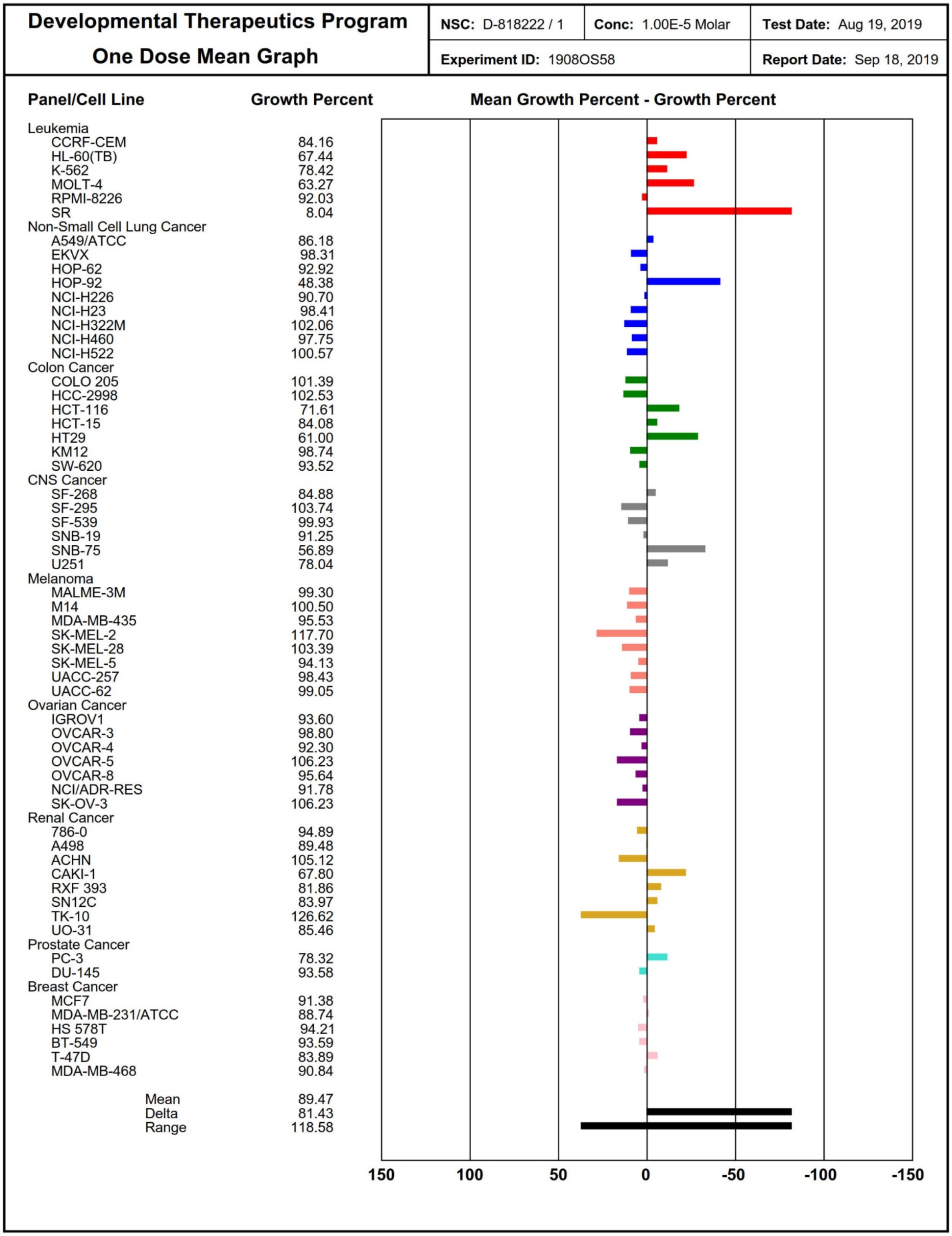

Figure S4. Anticancer screening data of compound **7** at single dose assay

Developmental Therapeutics Program	NSC: D-818177 / 1	Conc: 1.00E-5 Molar	Test Date: Aug 19, 2019
One Dose Mean Graph	Experiment ID: 1908OS58		Report Date: Sep 18, 2019

Panel/Cell Line	Growth Percent
Leukemia	
CCRF-CEM	92.78
HL-60(TB)	113.54
K-562	86.24
MOLT-4	94.00
RPMI-8226	86.30
SR	81.29
Non-Small Cell Lung Cancer	
A549/ATCC	97.63
EKVX	100.74
HOP-62	98.64
HOP-92	81.76
NCI-H226	97.54
NCI-H23	101.99
NCI-H322M	98.98
NCI-H460	99.22
NCI-H522	96.64
Colon Cancer	
COLO 205	120.63
HCC-2998	106.73
HCT-116	94.52
HCT-15	104.23
HT29	101.58
KM12	99.20
SW-620	106.32
CNS Cancer	
SF-268	91.51
SF-295	102.14
SF-539	95.57
SNB-19	88.84
SNB-75	102.06
U251	98.81
Melanoma	
MALME-3M	98.97
M14	97.13
MDA-MB-435	101.40
SK-MEL-2	118.13
SK-MEL-28	108.30
SK-MEL-5	99.84
UACC-257	106.03
UACC-62	89.74
Ovarian Cancer	
IGROV1	92.52
OVCAR-3	110.02
OVCAR-4	110.01
OVCAR-5	114.78
OVCAR-8	101.17
NCI/ADR-RES	100.51
SK-OV-3	103.26
Renal Cancer	
786-0	101.14
A498	100.81
ACHN	99.91
CAKI-1	53.80
RXF 393	101.71
SN12C	92.17
TK-10	146.79
UO-31	80.29
Prostate Cancer	
PC-3	80.80
DU-145	105.55
Breast Cancer	
MCF7	99.37
MDA-MB-231/ATCC	88.41
HS 578T	99.42
BT-549	96.70
T-47D	105.03
MDA-MB-468	110.83
Mean	99.22
Delta	45.42
Range	92.99

Mean Growth Percent - Growth Percent

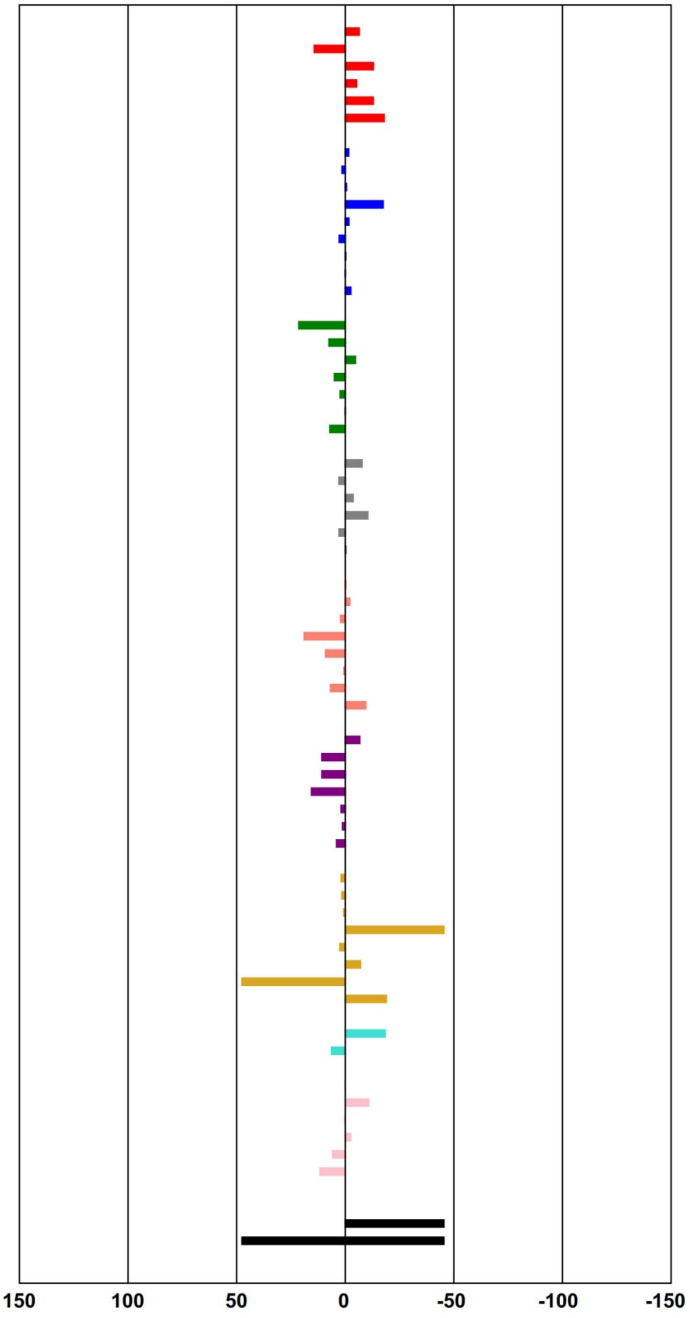

Figure S5. Anticancer screening data of compound **8** at single dose assay

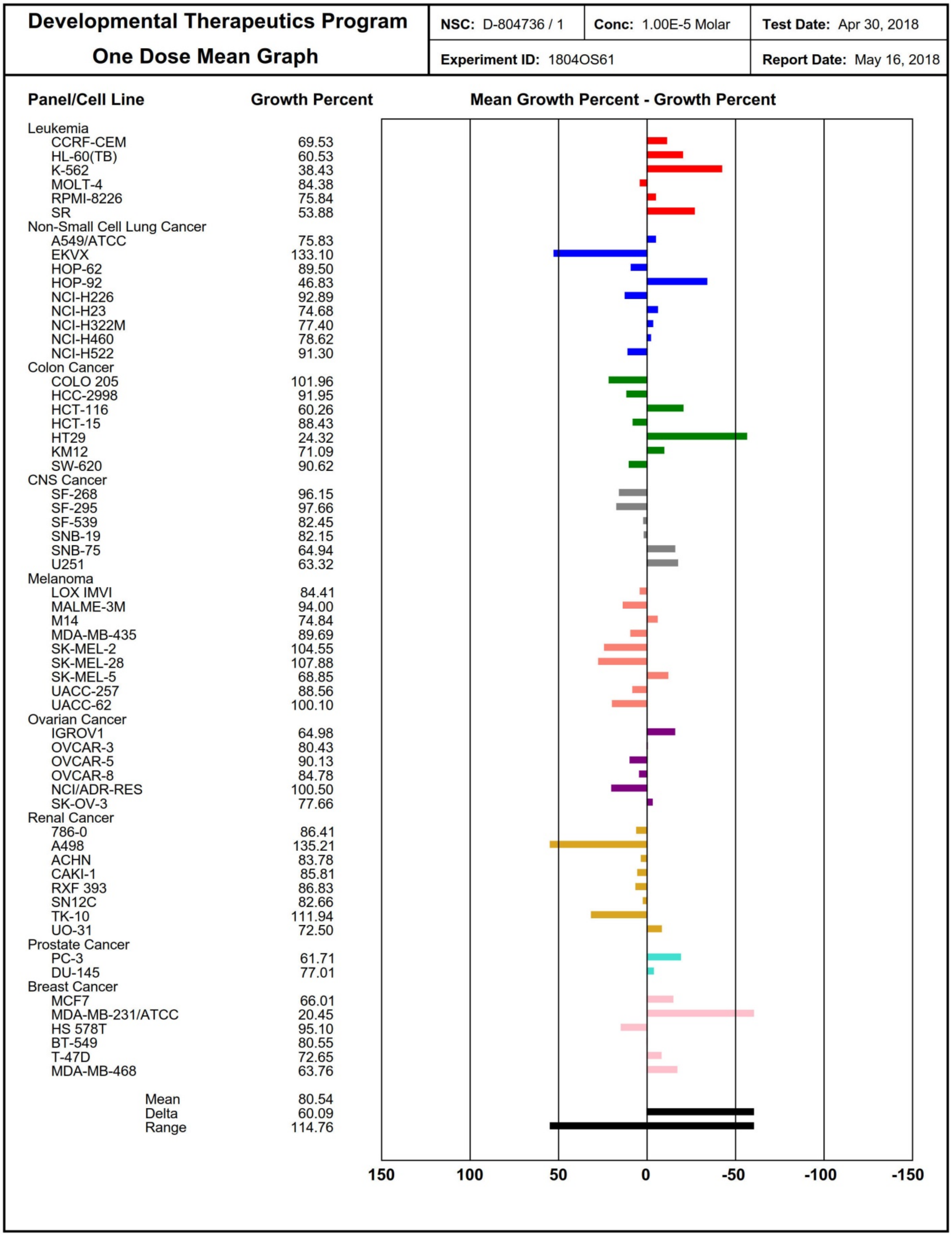

Figure S6. Anticancer screening data of compound **9** at single dose assay

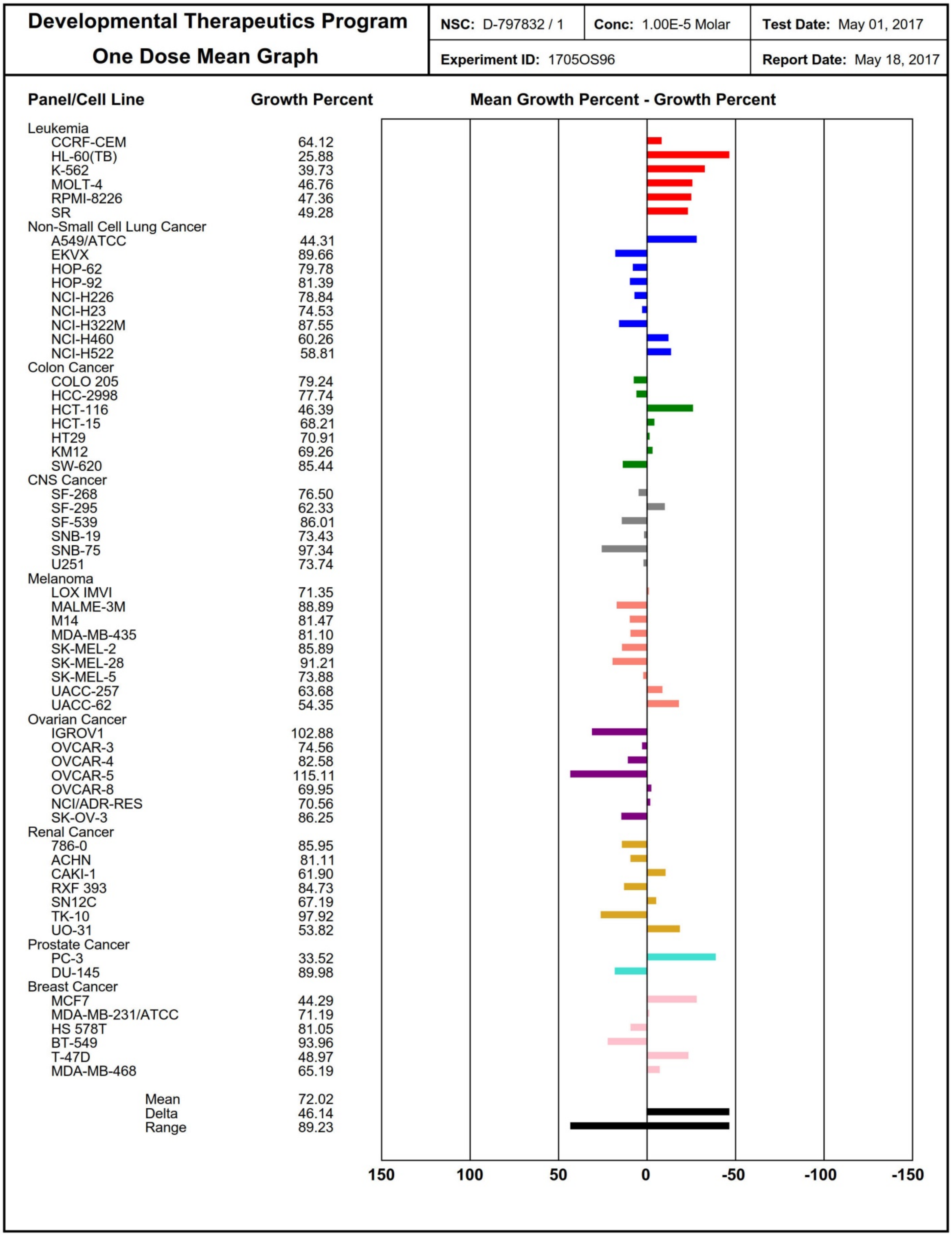

Figure S7. Anticancer screening data of compound **12** at single dose assay

Developmental Therapeutics Program One Dose Mean Graph	NSC: D-818184 / 1	Conc: 1.00E-5 Molar	Test Date: Aug 19, 2019
	Experiment ID: 1908OS58		Report Date: Sep 18, 2019

Panel/Cell Line	Growth Percent
**Leukemia**	
CCRF-CEM	91.28
HL-60(TB)	101.24
K-562	86.50
MOLT-4	83.63
RPMI-8226	77.24
SR	71.81
**Non-Small Cell Lung Cancer**	
A549/ATCC	99.06
EKVX	100.92
HOP-62	95.59
HOP-92	80.83
NCI-H226	105.19
NCI-H23	100.46
NCI-H322M	101.71
NCI-H460	99.74
NCI-H522	105.31
**Colon Cancer**	
COLO 205	115.14
HCC-2998	103.43
HCT-116	87.20
HCT-15	105.36
HT29	109.90
KM12	98.98
SW-620	103.38
**CNS Cancer**	
SF-268	89.68
SF-295	102.60
SF-539	99.72
SNB-19	98.02
SNB-75	79.89
U251	92.77
**Melanoma**	
MALME-3M	106.71
M14	97.09
MDA-MB-435	100.10
SK-MEL-2	115.74
SK-MEL-28	105.67
SK-MEL-5	102.97
UACC-257	103.41
UACC-62	93.50
**Ovarian Cancer**	
IGROV1	100.38
OVCAR-3	101.43
OVCAR-4	105.72
OVCAR-5	108.77
OVCAR-8	99.00
NCI/ADR-RES	101.81
SK-OV-3	104.00
**Renal Cancer**	
786-0	103.93
A498	95.86
ACHN	101.41
CAKI-1	59.08
RXF 393	108.47
SN12C	99.06
TK-10	140.73
UO-31	104.59
**Prostate Cancer**	
PC-3	78.17
DU-145	105.31
**Breast Cancer**	
MCF7	97.60
MDA-MB-231/ATCC	98.99
HS 578T	103.62
BT-549	95.56
T-47D	103.58
MDA-MB-468	108.60
Mean	98.94
Delta	39.86
Range	81.65

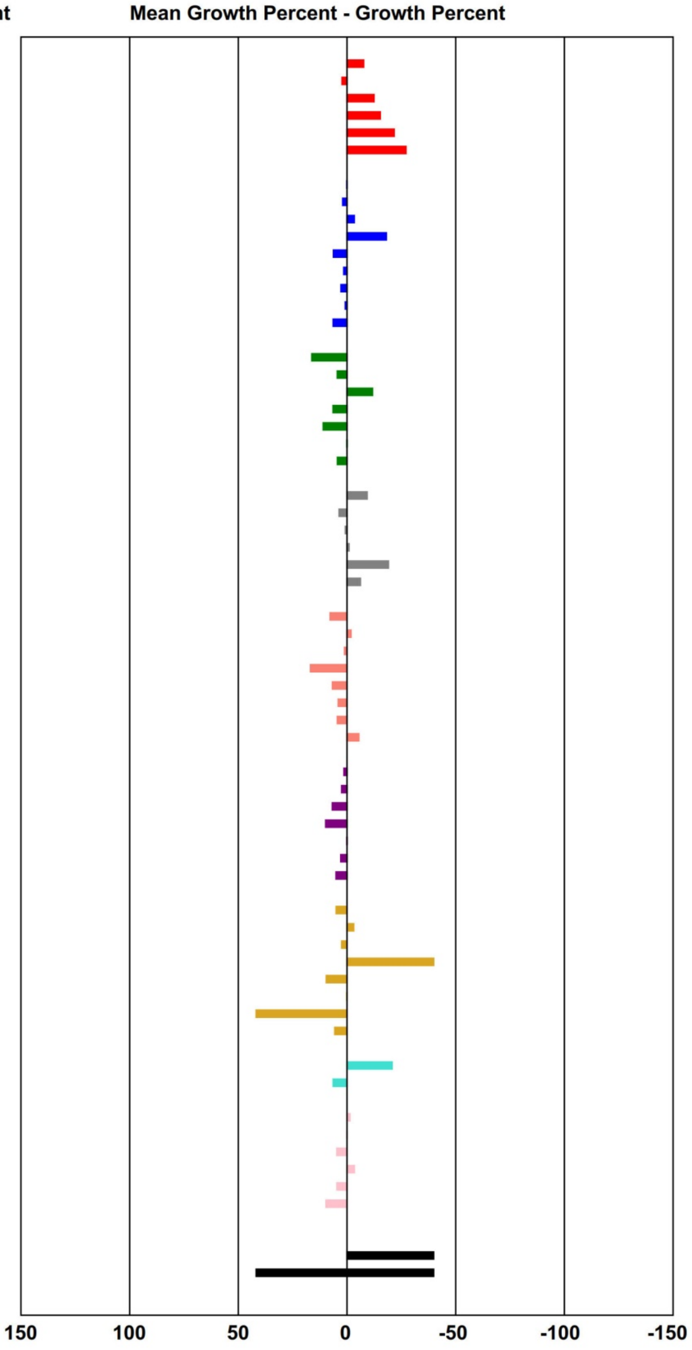

Figure S8. Anticancer screening data of compound **13** at single dose assay

Developmental Therapeutics Program<br>One Dose Mean Graph	**NSC:** D-812001 / 1	**Conc:** 1.00E-5 Molar	**Test Date:** Jan 14, 2019
	**Experiment ID:** 1901OS55		**Report Date:** Feb 04, 2019

Panel/Cell Line	Growth Percent
**Leukemia**	
CCRF-CEM	107.65
HL-60(TB)	99.09
K-562	99.38
MOLT-4	96.23
RPMI-8226	102.32
SR	91.58
**Non-Small Cell Lung Cancer**	
A549/ATCC	110.76
EKVX	104.17
HOP-62	108.39
HOP-92	101.65
NCI-H226	103.05
NCI-H23	101.90
NCI-H322M	103.75
NCI-H460	107.41
NCI-H522	93.77
**Colon Cancer**	
COLO 205	120.27
HCC-2998	108.77
HCT-116	97.62
HCT-15	109.83
HT29	114.16
KM12	111.01
SW-620	108.17
**CNS Cancer**	
SF-268	102.92
SF-295	110.43
SF-539	103.21
SNB-19	96.48
SNB-75	104.03
U251	112.70
**Melanoma**	
LOX IMVI	103.38
MALME-3M	105.49
M14	108.53
MDA-MB-435	106.29
SK-MEL-2	112.35
SK-MEL-28	108.50
SK-MEL-5	99.56
UACC-257	118.03
UACC-62	95.86
**Ovarian Cancer**	
IGROV1	106.28
OVCAR-3	114.78
OVCAR-4	110.27
OVCAR-5	108.20
OVCAR-8	107.62
NCI/ADR-RES	107.69
SK-OV-3	112.66
**Renal Cancer**	
786-0	103.32
A498	92.60
ACHN	109.67
CAKI-1	90.68
RXF 393	112.28
SN12C	99.50
TK-10	113.08
UO-31	85.83
**Prostate Cancer**	
PC-3	90.06
DU-145	104.31
**Breast Cancer**	
MCF7	103.75
MDA-MB-231/ATCC	95.50
HS 578T	104.02
BT-549	111.33
T-47D	101.10
MDA-MB-468	112.65
Mean	104.76
Delta	18.93
Range	34.44

Mean Growth Percent - Growth Percent

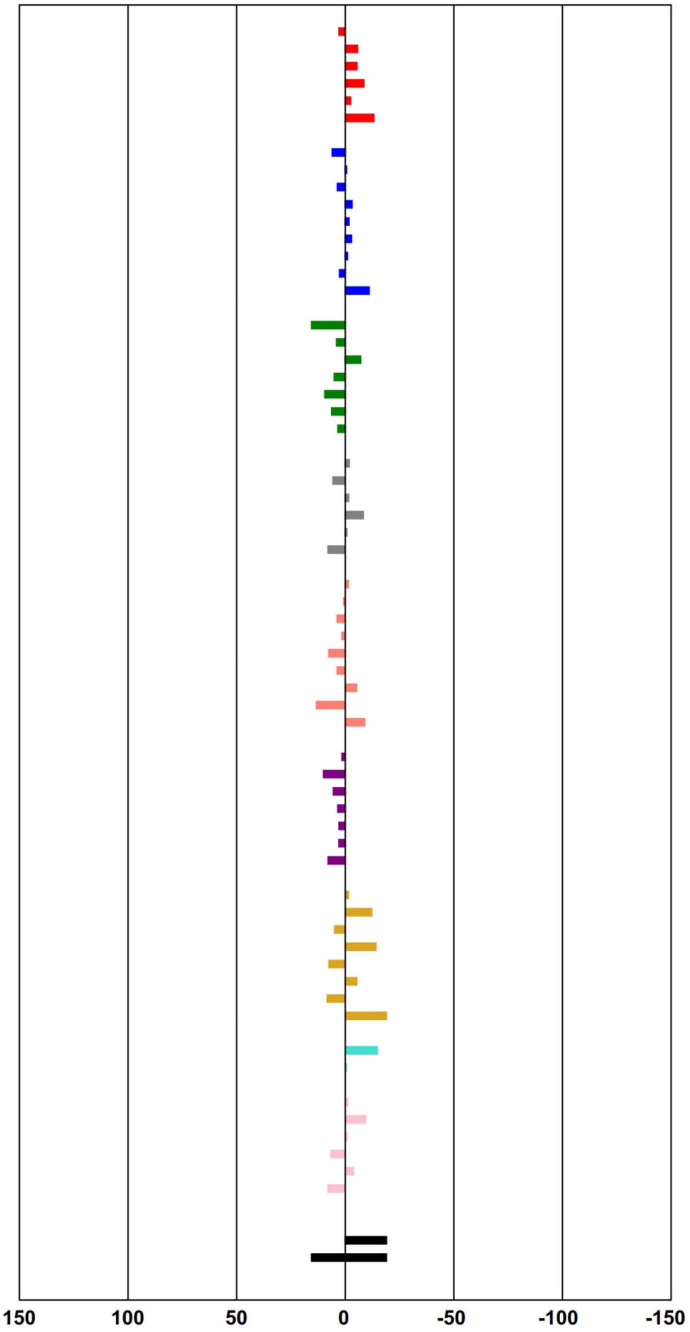

Figure S9. Anticancer screening data of compound **14** at single dose assay

Developmental Therapeutics Program	NSC: D-804732 / 1	Conc: 1.00E-5 Molar	Test Date: Apr 30, 2018
One Dose Mean Graph	Experiment ID: 1804OS61		Report Date: May 16, 2018

Panel/Cell Line	Growth Percent
Leukemia	
CCRF-CEM	14.95
HL-60(TB)	-14.34
K-562	38.12
MOLT-4	11.67
RPMI-8226	22.20
SR	52.40
Non-Small Cell Lung Cancer	
A549/ATCC	32.05
EKVX	53.47
HOP-62	45.43
HOP-92	8.07
NCI-H226	44.25
NCI-H23	37.13
NCI-H322M	73.79
NCI-H460	20.60
NCI-H522	39.93
Colon Cancer	
COLO 205	57.66
HCC-2998	60.47
HCT-116	17.53
HCT-15	50.35
HT29	23.68
KM12	34.26
SW-620	28.83
CNS Cancer	
SF-268	49.82
SF-295	31.97
SF-539	50.14
SNB-19	47.82
SNB-75	23.86
U251	27.93
Melanoma	
LOX IMVI	39.57
MALME-3M	64.30
M14	54.10
MDA-MB-435	36.75
SK-MEL-2	41.70
SK-MEL-28	56.29
SK-MEL-5	-8.44
UACC-257	36.95
UACC-62	48.24
Ovarian Cancer	
IGROV1	34.67
OVCAR-3	32.27
OVCAR-5	60.41
OVCAR-8	44.44
NCI/ADR-RES	52.22
SK-OV-3	49.85
Renal Cancer	
786-0	52.62
A498	58.70
ACHN	23.51
CAKI-1	18.57
RXF 393	35.88
SN12C	39.89
TK-10	54.02
UO-31	17.41
Prostate Cancer	
PC-3	19.12
DU-145	53.46
Breast Cancer	
MCF7	22.21
MDA-MB-231/ATCC	-33.78
HS 578T	37.82
BT-549	54.16
T-47D	28.02
MDA-MB-468	53.53
Mean	36.65
Delta	70.43
Range	107.57

Mean Growth Percent - Growth Percent

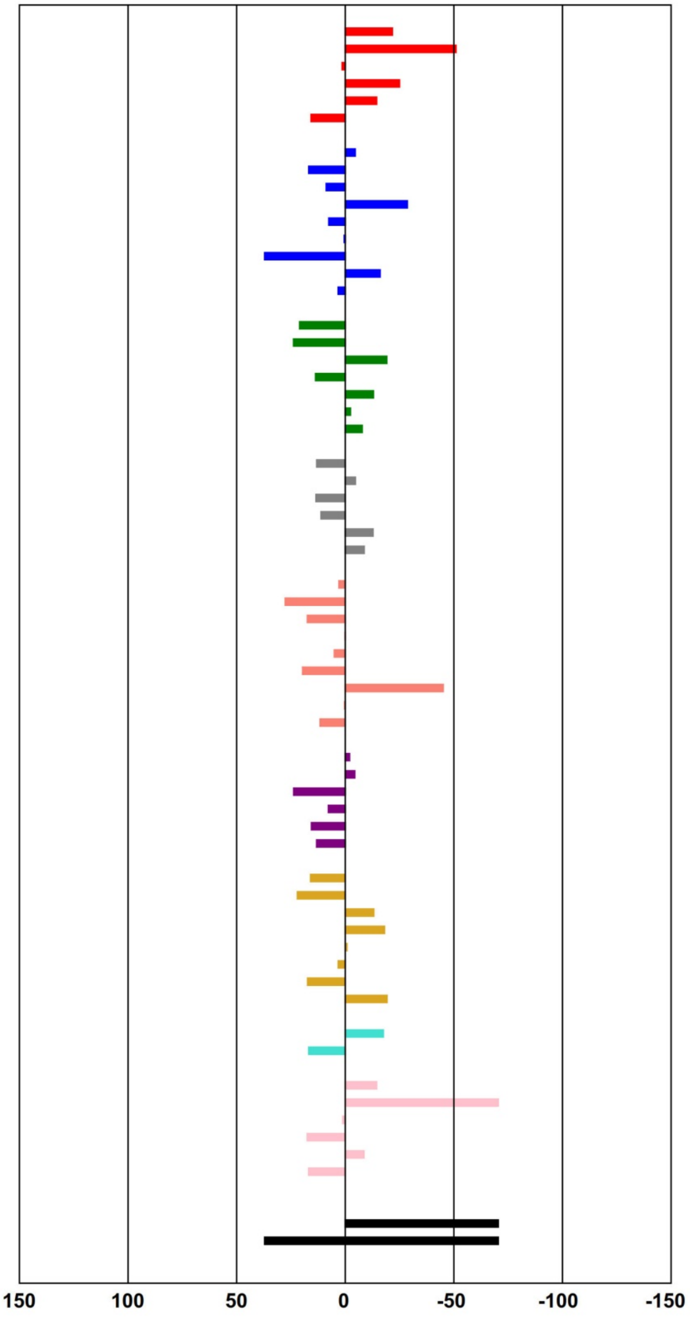

Figure S10. Anticancer screening data of compound **15** at single dose assay

Developmental Therapeutics Program	NSC: D-804729 / 1	Conc: 1.00E-5 Molar	Test Date: Apr 30, 2018
One Dose Mean Graph	Experiment ID: 1804OS61		Report Date: May 16, 2018

Panel/Cell Line	Growth Percent
Leukemia	
CCRF-CEM	95.59
HL-60(TB)	86.70
K-562	84.16
MOLT-4	76.69
RPMI-8226	79.64
SR	79.77
Non-Small Cell Lung Cancer	
A549/ATCC	86.44
EKVX	107.09
HOP-62	75.77
HOP-92	59.06
NCI-H226	93.37
NCI-H23	95.67
NCI-H322M	102.03
NCI-H460	94.21
NCI-H522	80.89
Colon Cancer	
COLO 205	99.85
HCC-2998	95.04
HCT-116	89.90
HCT-15	114.03
HT29	96.44
KM12	100.82
SW-620	102.97
CNS Cancer	
SF-268	83.82
SF-295	91.85
SF-539	79.54
SNB-19	80.21
SNB-75	56.79
U251	85.51
Melanoma	
LOX IMVI	105.79
MALME-3M	92.66
M14	105.18
MDA-MB-435	92.15
SK-MEL-2	87.64
SK-MEL-28	105.60
SK-MEL-5	88.75
UACC-257	89.32
UACC-62	69.15
Ovarian Cancer	
IGROV1	89.47
OVCAR-3	107.16
OVCAR-5	112.68
OVCAR-8	88.82
NCI/ADR-RES	100.39
SK-OV-3	80.15
Renal Cancer	
786-0	87.21
A498	100.75
ACHN	95.91
CAKI-1	70.59
RXF 393	101.18
SN12C	85.33
TK-10	101.72
UO-31	83.65
Prostate Cancer	
PC-3	69.25
DU-145	104.35
Breast Cancer	
MCF7	94.79
MDA-MB-231/ATCC	79.84
HS 578T	73.54
BT-549	93.90
T-47D	82.86
MDA-MB-468	102.71
Mean	90.11
Delta	33.32
Range	57.24

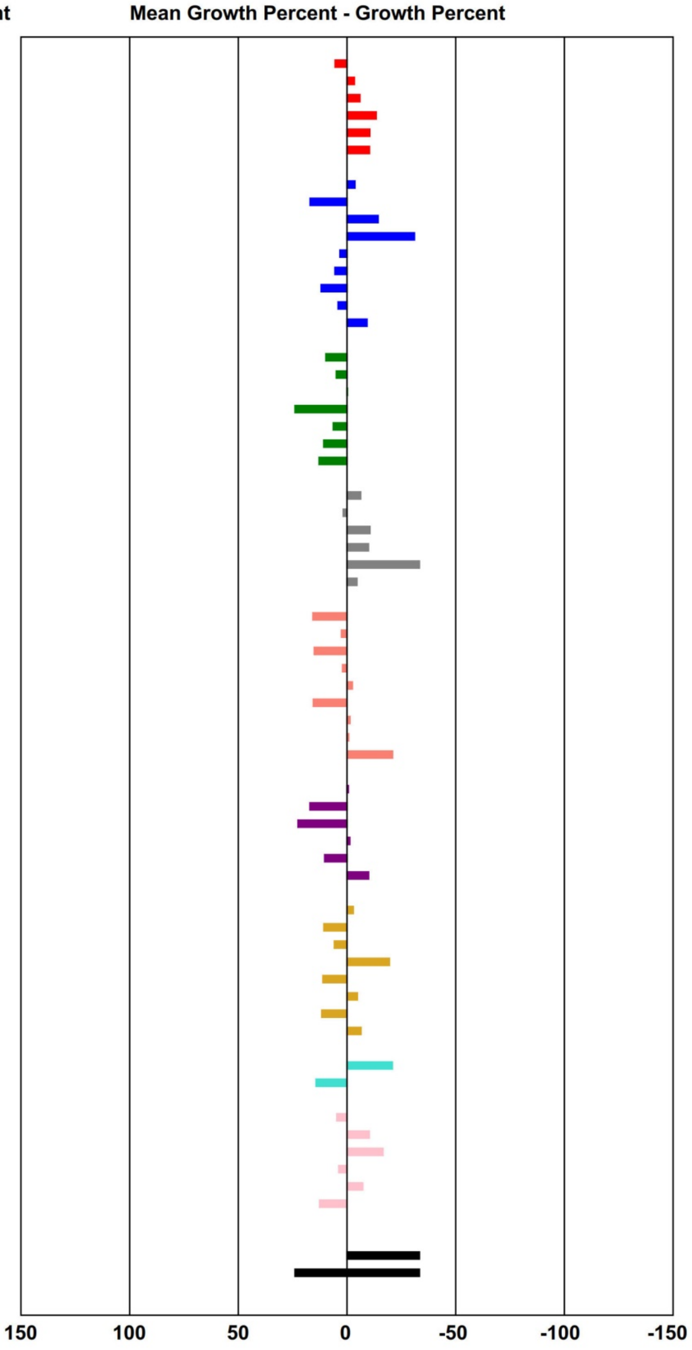

Figure S11. Anticancer screening data of compound **16** at single dose assay

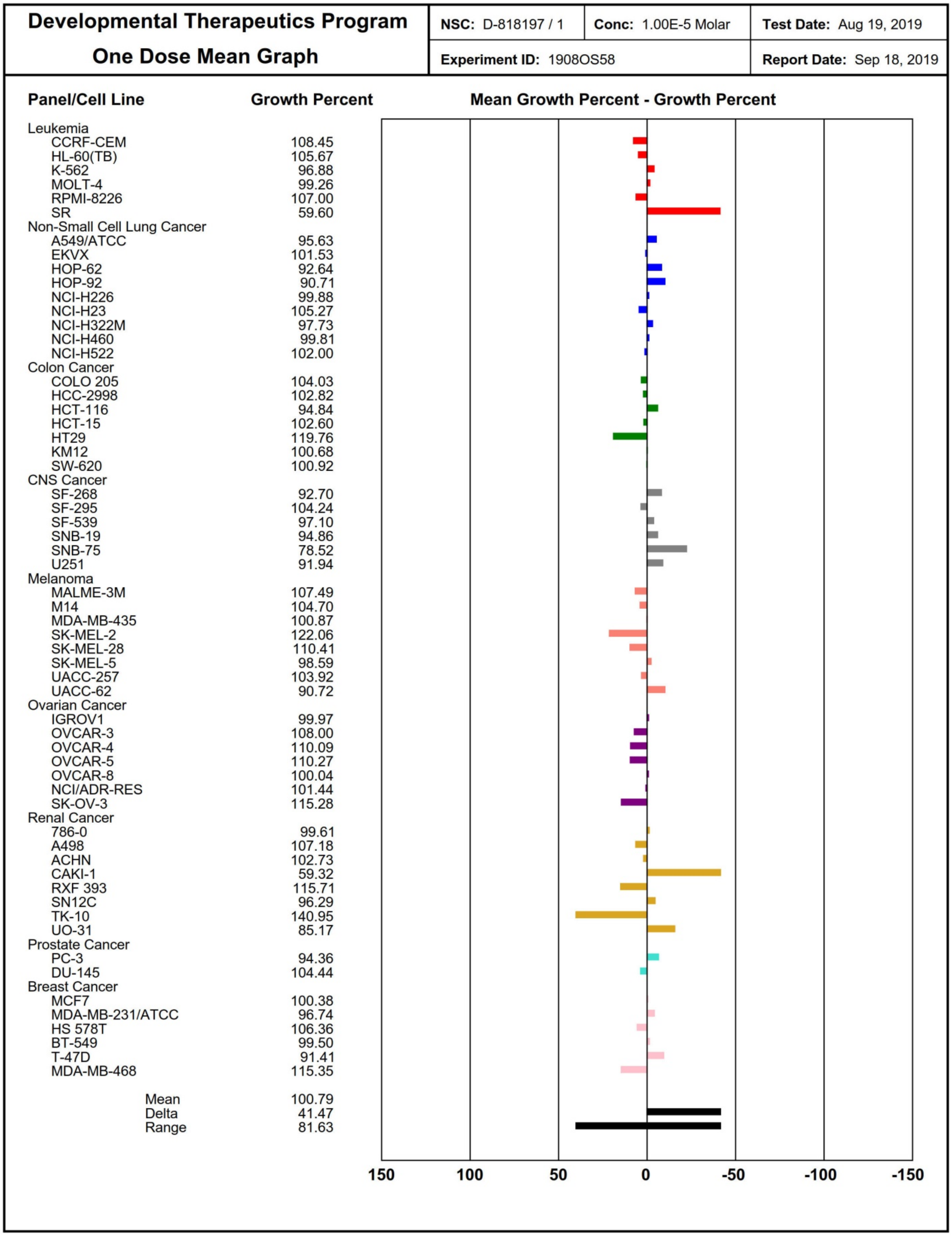

Figure S12. Anticancer screening data of compound **17** at single dose assay

Developmental Therapeutics Program	NSC: D-818169 / 1	Conc: 1.00E-5 Molar	Test Date: Aug 19, 2019
One Dose Mean Graph	Experiment ID: 1908OS58		Report Date: Sep 18, 2019

Panel/Cell Line	Growth Percent
Leukemia	
CCRF-CEM	102.24
HL-60(TB)	103.94
K-562	91.78
MOLT-4	96.91
RPMI-8226	98.93
SR	81.53
Non-Small Cell Lung Cancer	
A549/ATCC	98.05
EKVX	99.35
HOP-62	101.18
HOP-92	81.72
NCI-H226	95.79
NCI-H23	101.85
NCI-H322M	97.10
NCI-H460	100.02
NCI-H522	94.93
Colon Cancer	
COLO 205	111.20
HCC-2998	97.65
HCT-116	97.70
HCT-15	105.70
HT29	107.99
KM12	101.01
SW-620	107.31
CNS Cancer	
SF-268	94.86
SF-295	99.95
SF-539	98.48
SNB-19	93.06
SNB-75	86.25
U251	95.88
Melanoma	
MALME-3M	97.09
M14	97.91
MDA-MB-435	102.41
SK-MEL-2	106.24
SK-MEL-28	104.62
SK-MEL-5	99.78
UACC-257	103.27
UACC-62	88.27
Ovarian Cancer	
IGROV1	106.13
OVCAR-3	111.14
OVCAR-4	104.50
OVCAR-5	103.30
OVCAR-8	101.70
NCI/ADR-RES	103.41
SK-OV-3	110.99
Renal Cancer	
786-0	94.34
A498	102.36
ACHN	103.02
CAKI-1	79.25
RXF 393	111.36
SN12C	99.25
TK-10	91.95
UO-31	95.97
Prostate Cancer	
PC-3	92.58
DU-145	107.09
Breast Cancer	
MCF7	94.03
MDA-MB-231/ATCC	99.50
HS 578T	98.53
BT-549	97.35
T-47D	72.48
MDA-MB-468	103.55
Mean	98.74
Delta	26.26
Range	38.88

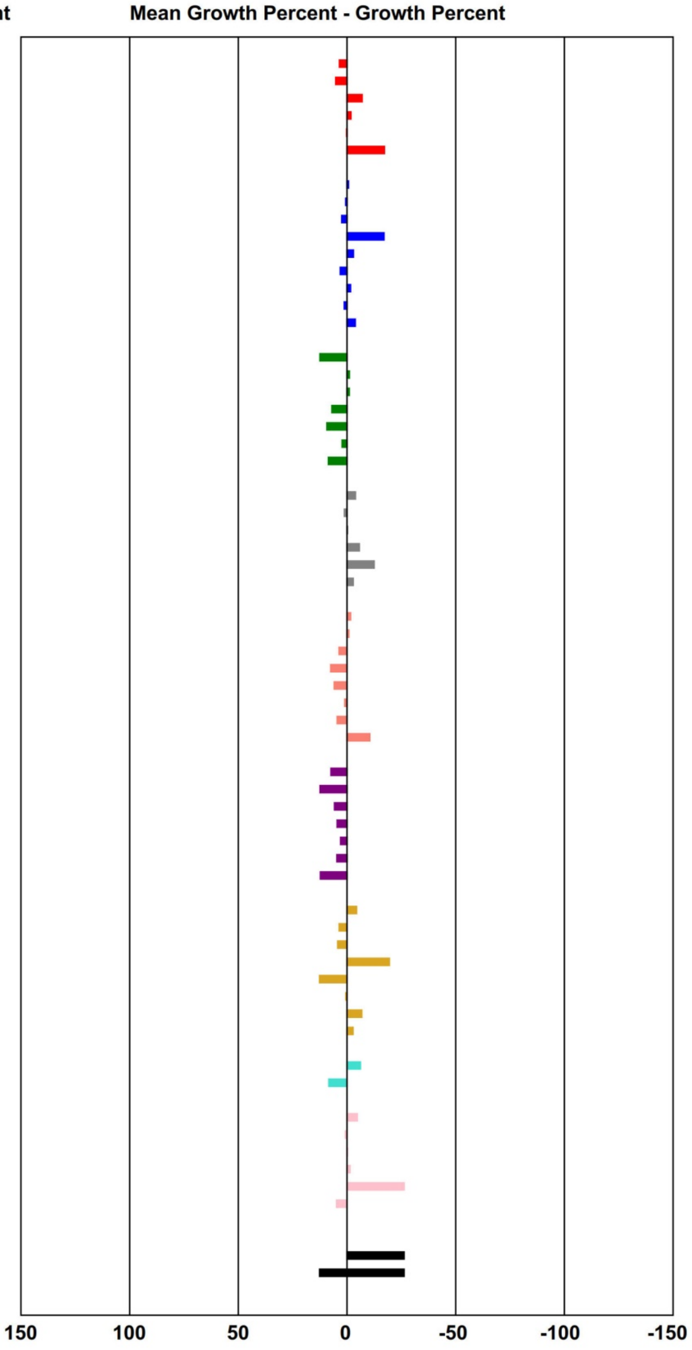

Figure S13. Anticancer screening data of compound **19** at single dose assay

Developmental Therapeutics Program One Dose Mean Graph	NSC: D-804740 / 1	Conc: 1.00E-5 Molar	Test Date: Apr 30, 2018
	Experiment ID: 1804OS61		Report Date: May 16, 2018

Panel/Cell Line	Growth Percent
Leukemia	
CCRF-CEM	11.76
HL-60(TB)	36.98
K-562	1.52
MOLT-4	10.06
RPMI-8226	29.30
SR	18.41
Non-Small Cell Lung Cancer	
A549/ATCC	46.22
EKVX	140.99
HOP-62	93.23
HOP-92	76.12
NCI-H226	111.19
NCI-H23	77.41
NCI-H322M	80.66
NCI-H460	-16.23
NCI-H522	88.83
Colon Cancer	
COLO 205	18.88
HCC-2998	77.15
HCT-116	12.60
HCT-15	81.84
HT29	4.21
KM12	31.48
SW-620	7.33
CNS Cancer	
SF-268	99.10
SF-295	92.19
SF-539	79.88
SNB-19	80.84
SNB-75	84.53
U251	-39.45
Melanoma	
LOX IMVI	5.71
MALME-3M	43.73
M14	106.13
MDA-MB-435	89.65
SK-MEL-2	121.60
SK-MEL-28	101.30
SK-MEL-5	85.91
UACC-257	88.02
UACC-62	120.26
Ovarian Cancer	
IGROV1	27.33
OVCAR-3	71.78
OVCAR-5	90.51
OVCAR-8	-28.27
NCI/ADR-RES	86.28
SK-OV-3	97.90
Renal Cancer	
786-0	61.92
A498	130.23
ACHN	75.62
CAKI-1	94.49
RXF 393	69.57
SN12C	81.86
TK-10	119.40
UO-31	53.41
Prostate Cancer	
PC-3	60.88
DU-145	54.99
Breast Cancer	
MCF7	14.49
MDA-MB-231/ATCC	37.29
HS 578T	94.90
BT-549	118.02
T-47D	85.31
MDA-MB-468	21.21
Mean	63.02
Delta	102.47
Range	180.44

Mean Growth Percent - Growth Percent

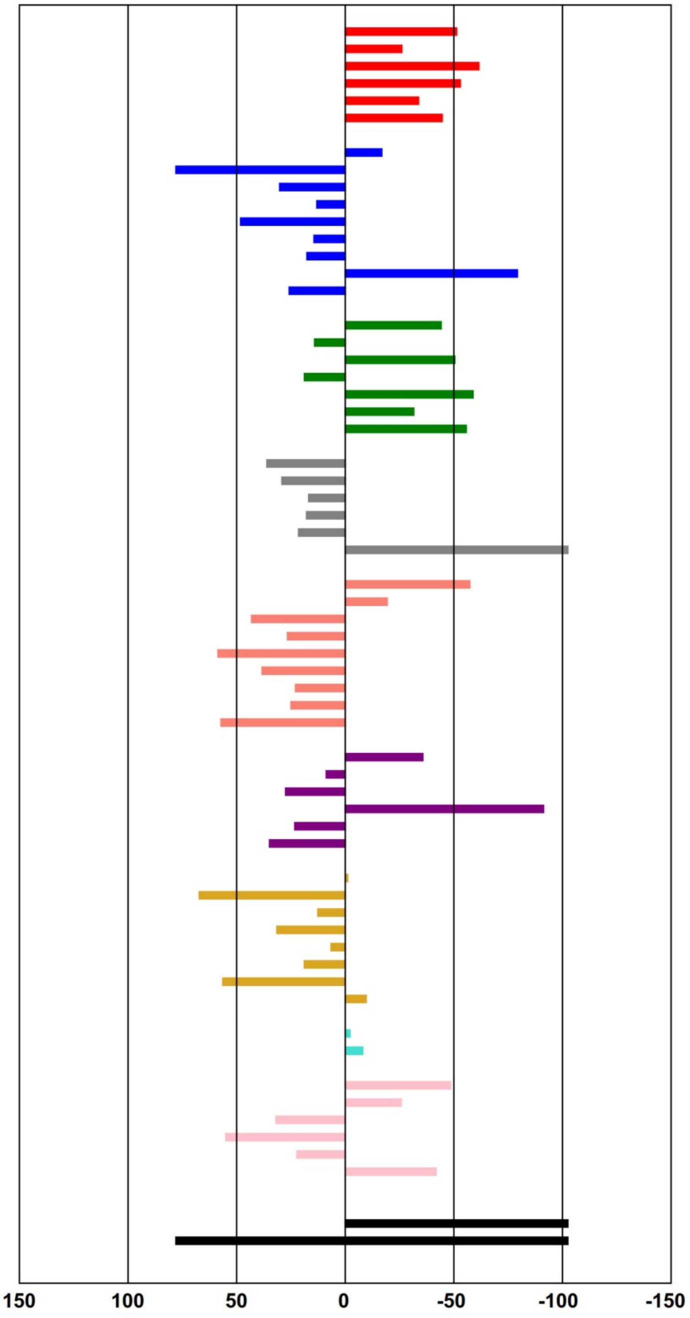

Figure S14. Anticancer screening data of compound **20** at single dose assay

Developmental Therapeutics Program	NSC: D-811966 / 1	Conc: 1.00E-5 Molar	Test Date: Jan 14, 2019
One Dose Mean Graph	Experiment ID: 1901OS55		Report Date: Feb 04, 2019

Panel/Cell Line	Growth Percent
**Leukemia**	
CCRF-CEM	101.95
HL-60(TB)	102.44
K-562	101.22
MOLT-4	98.60
RPMI-8226	99.47
SR	92.72
**Non-Small Cell Lung Cancer**	
A549/ATCC	101.50
EKVX	95.32
HOP-62	90.56
HOP-92	99.30
NCI-H226	102.37
NCI-H23	104.13
NCI-H322M	100.70
NCI-H460	103.73
NCI-H522	93.66
**Colon Cancer**	
COLO 205	109.57
HCC-2998	102.26
HCT-116	99.51
HCT-15	106.93
HT29	104.75
KM12	105.71
SW-620	101.83
**CNS Cancer**	
SF-268	98.43
SF-295	100.95
SF-539	104.28
SNB-19	95.32
SNB-75	90.74
U251	100.16
**Melanoma**	
LOX IMVI	100.98
MALME-3M	106.36
M14	92.88
MDA-MB-435	102.75
SK-MEL-2	105.79
SK-MEL-28	106.45
SK-MEL-5	101.35
UACC-257	103.04
UACC-62	97.49
**Ovarian Cancer**	
IGROV1	103.57
OVCAR-3	105.51
OVCAR-4	105.31
OVCAR-5	100.06
OVCAR-8	102.51
NCI/ADR-RES	107.32
SK-OV-3	93.83
**Renal Cancer**	
786-0	98.03
A498	101.96
ACHN	103.15
CAKI-1	87.76
RXF 393	108.12
SN12C	98.70
TK-10	101.74
UO-31	90.62
**Prostate Cancer**	
PC-3	95.33
DU-145	104.82
**Breast Cancer**	
MCF7	94.11
MDA-MB-231/ATCC	99.39
HS 578T	101.60
BT-549	111.05
T-47D	92.03
MDA-MB-468	99.98
Mean	100.53
Delta	12.77
Range	23.29

Mean Growth Percent - Growth Percent

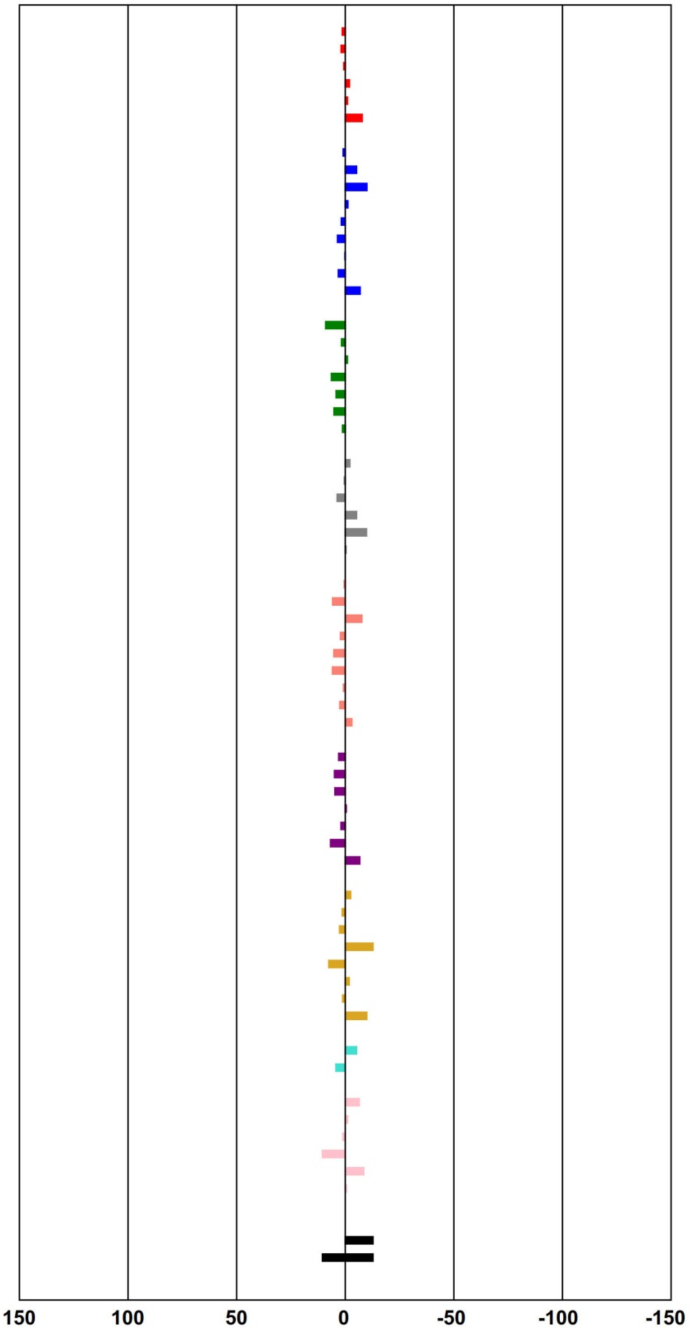

Figure S15. Anticancer screening data of compound **22** at single dose assay

Developmental Therapeutics Program<br>One Dose Mean Graph	NSC: D-818181 / 1	Conc: 1.00E-5 Molar	Test Date: Aug 19, 2019
	Experiment ID: 1908OS58		Report Date: Sep 18, 2019

Panel/Cell Line	Growth Percent
Leukemia	
CCRF-CEM	105.67
HL-60(TB)	99.88
K-562	94.97
MOLT-4	100.46
RPMI-8226	105.28
SR	83.72
Non-Small Cell Lung Cancer	
A549/ATCC	99.72
EKVX	100.38
HOP-62	97.50
HOP-92	96.83
NCI-H226	100.25
NCI-H23	105.14
NCI-H322M	102.17
NCI-H460	100.06
NCI-H522	97.34
Colon Cancer	
COLO 205	111.35
HCC-2998	103.45
HCT-116	101.23
HCT-15	99.52
HT29	115.05
KM12	101.77
SW-620	101.44
CNS Cancer	
SF-268	96.31
SF-295	101.19
SF-539	101.95
SNB-19	96.74
SNB-75	80.54
U251	101.17
Melanoma	
MALME-3M	105.50
M14	104.16
MDA-MB-435	99.13
SK-MEL-2	123.09
SK-MEL-28	108.58
SK-MEL-5	97.39
UACC-257	101.74
UACC-62	96.93
Ovarian Cancer	
IGROV1	106.25
OVCAR-3	108.12
OVCAR-4	104.52
OVCAR-5	106.67
OVCAR-8	104.88
NCI/ADR-RES	99.24
SK-OV-3	109.28
Renal Cancer	
786-0	98.55
A498	90.56
ACHN	103.67
CAKI-1	87.67
RXF 393	116.26
SN12C	97.18
TK-10	115.07
UO-31	97.79
Prostate Cancer	
PC-3	102.52
DU-145	105.06
Breast Cancer	
MCF7	98.07
MDA-MB-231/ATCC	100.09
HS 578T	105.59
BT-549	98.28
T-47D	103.41
MDA-MB-468	107.57
Mean	101.76
Delta	21.22
Range	42.55

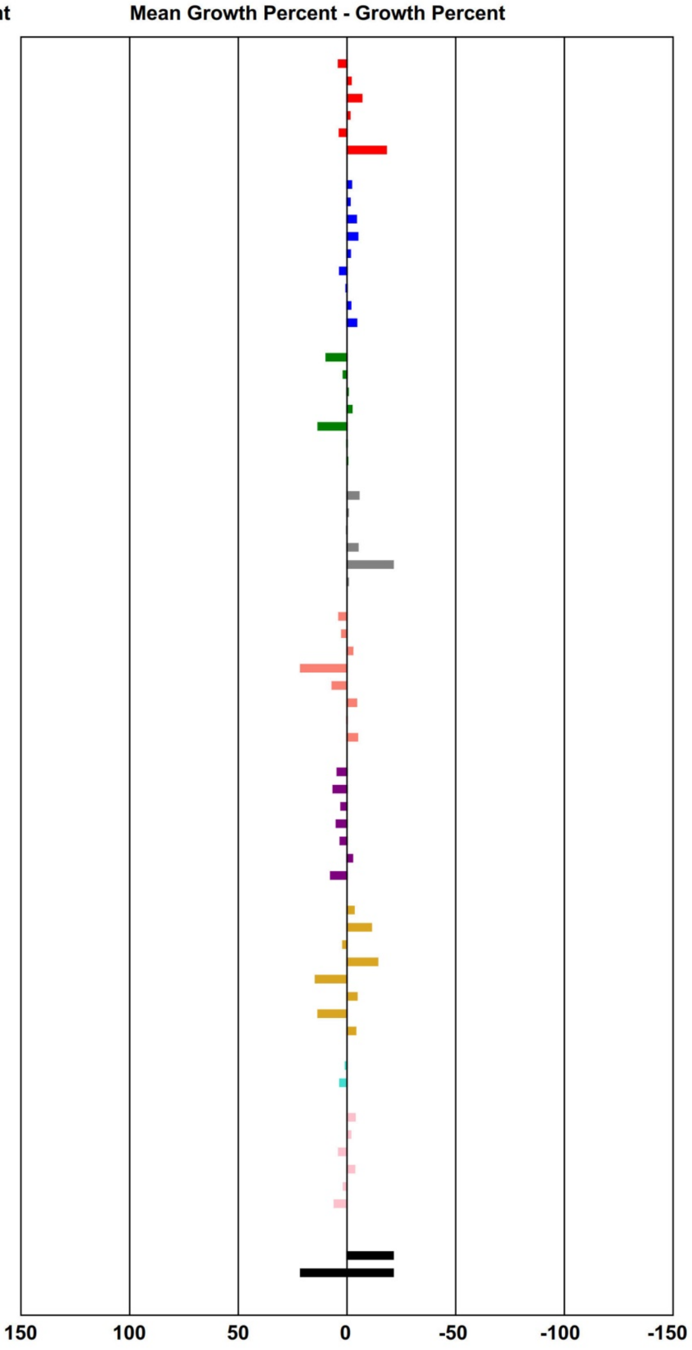

Figure S16. Anticancer screening data of compound **24** at single dose assay

Developmental Therapeutics Program<br>One Dose Mean Graph	NSC: D-818189 / 1	Conc: 1.00E-5 Molar	Test Date: Aug 19, 2019
	Experiment ID: 1908OS58		Report Date: Sep 18, 2019

Panel/Cell Line	Growth Percent
Leukemia	
CCRF-CEM	94.43
HL-60(TB)	89.61
K-562	20.11
MOLT-4	93.36
RPMI-8226	107.45
SR	21.69
Non-Small Cell Lung Cancer	
A549/ATCC	88.82
EKVX	102.40
HOP-62	92.90
HOP-92	92.39
NCI-H226	80.06
NCI-H23	82.49
NCI-H322M	100.22
NCI-H460	91.75
NCI-H522	99.64
Colon Cancer	
COLO 205	79.09
HCC-2998	99.96
HCT-116	77.72
HCT-15	82.56
HT29	38.95
KM12	98.20
SW-620	88.42
CNS Cancer	
SF-268	101.63
SF-295	91.17
SF-539	95.29
SNB-19	88.34
SNB-75	113.82
U251	91.55
Melanoma	
MALME-3M	102.85
M14	98.53
MDA-MB-435	94.85
SK-MEL-2	131.79
SK-MEL-28	110.73
SK-MEL-5	96.03
UACC-257	107.33
UACC-62	100.20
Ovarian Cancer	
IGROV1	112.34
OVCAR-3	106.03
OVCAR-4	101.84
OVCAR-5	111.54
OVCAR-8	83.78
NCI/ADR-RES	91.80
SK-OV-3	95.82
Renal Cancer	
786-0	107.21
A498	95.62
ACHN	104.87
CAKI-1	80.56
RXF 393	110.24
SN12C	91.06
TK-10	120.49
UO-31	92.07
Prostate Cancer	
PC-3	85.91
DU-145	101.21
Breast Cancer	
MCF7	89.37
MDA-MB-231/ATCC	104.41
HS 578T	103.57
BT-549	98.22
T-47D	90.85
MDA-MB-468	98.51
Mean	93.62
Delta	73.51
Range	111.68

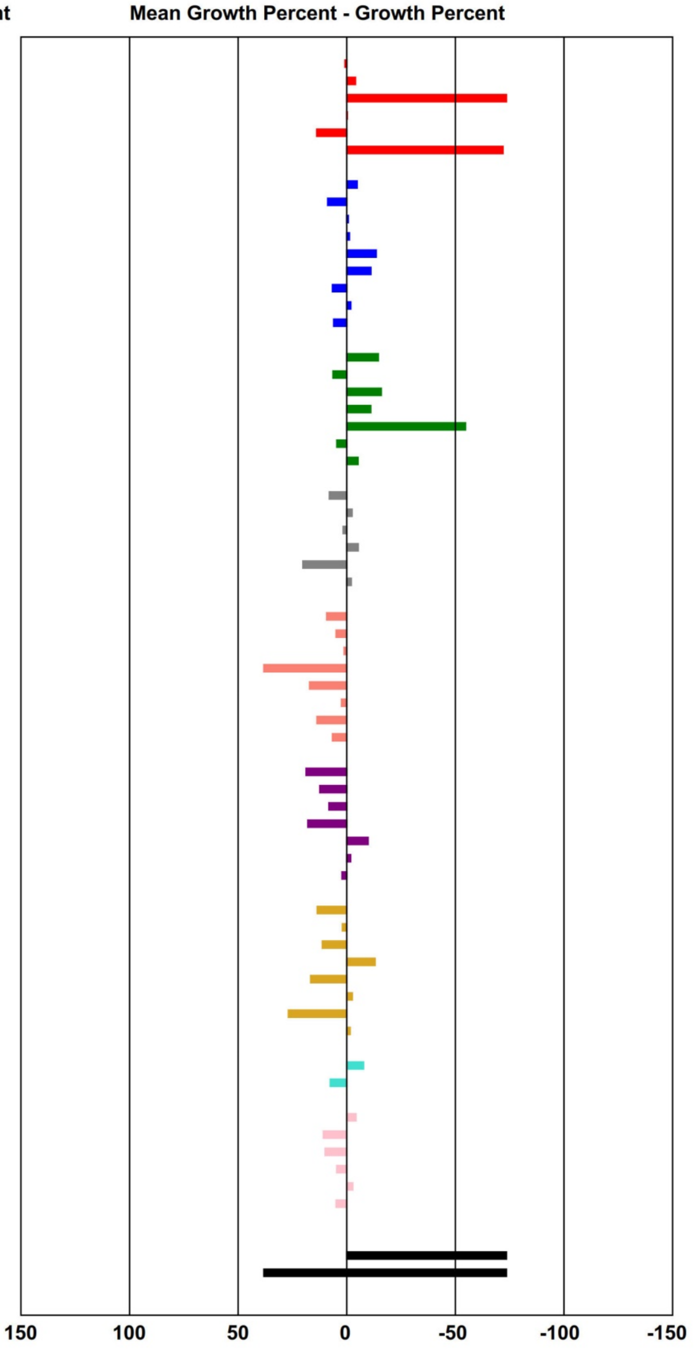

Figure S17. Anticancer screening data of compound **25** at single dose assay

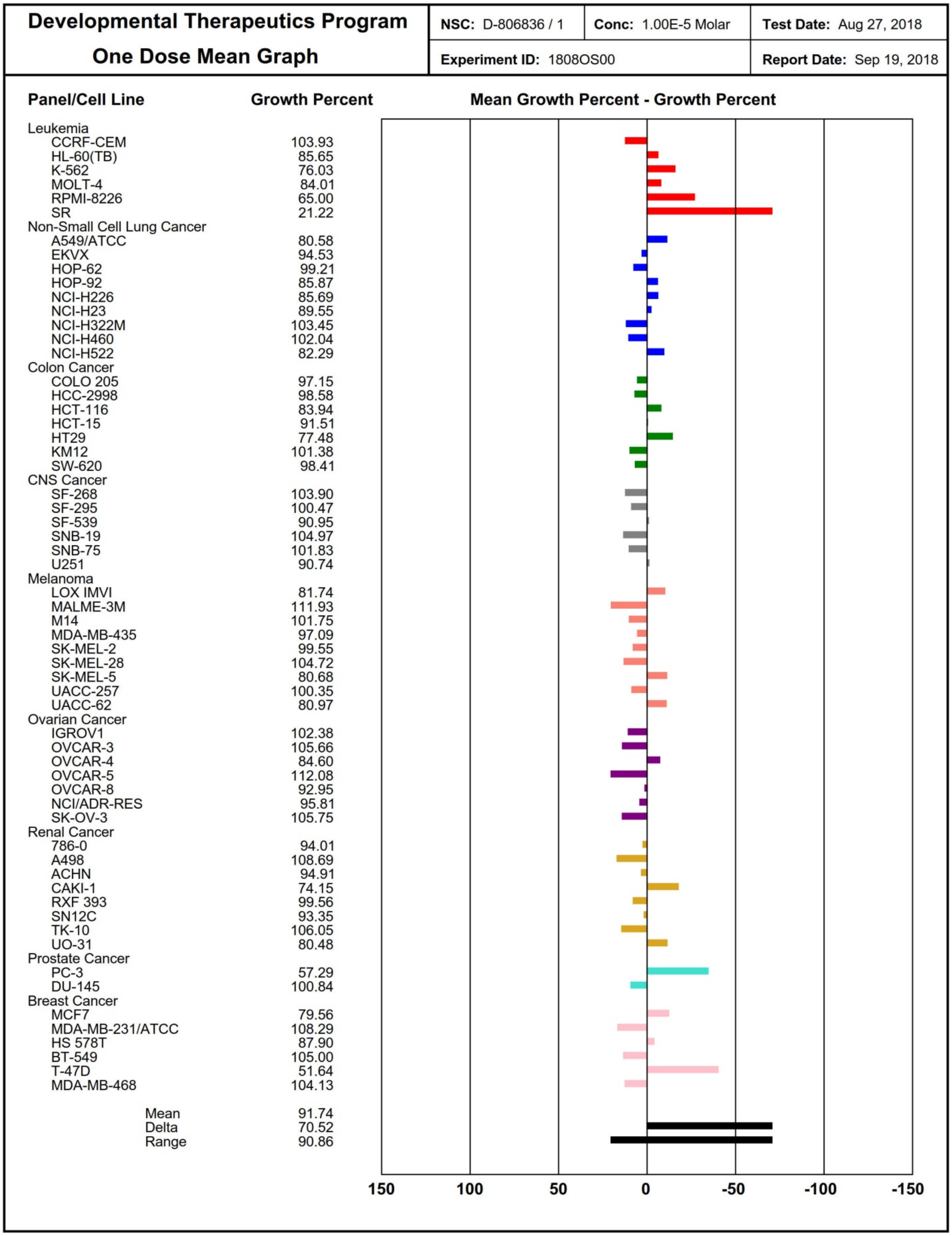

Figure S18. Anticancer screening data of compound **26** at single dose assay

Developmental Therapeutics Program One Dose Mean Graph	NSC: D-806849 / 1	Conc: 1.00E-5 Molar	Test Date: Aug 27, 2018
	Experiment ID: 1808OS00		Report Date: Sep 19, 2018

Panel/Cell Line	Growth Percent
Leukemia	
CCRF-CEM	84.74
HL-60(TB)	95.00
K-562	73.62
MOLT-4	70.32
RPMI-8226	69.64
SR	81.57
Non-Small Cell Lung Cancer	
A549/ATCC	76.47
EKVX	91.31
HOP-62	97.50
HOP-92	90.42
NCI-H226	86.34
NCI-H23	80.39
NCI-H322M	100.58
NCI-H460	88.90
NCI-H522	88.21
Colon Cancer	
COLO 205	109.60
HCC-2998	99.40
HCT-116	74.38
HCT-15	92.81
HT29	70.97
KM12	82.65
SW-620	87.25
CNS Cancer	
SF-268	95.77
SF-295	99.38
SF-539	85.59
SNB-19	97.07
SNB-75	117.69
U251	90.71
Melanoma	
LOX IMVI	90.20
MALME-3M	119.38
M14	87.94
MDA-MB-435	93.68
SK-MEL-2	103.40
SK-MEL-28	98.92
SK-MEL-5	87.11
UACC-257	100.05
UACC-62	87.38
Ovarian Cancer	
IGROV1	70.62
OVCAR-3	88.47
OVCAR-4	84.17
OVCAR-5	118.34
OVCAR-8	97.49
NCI/ADR-RES	103.76
SK-OV-3	97.66
Renal Cancer	
786-0	107.69
A498	114.07
ACHN	77.63
CAKI-1	66.39
RXF 393	99.85
SN12C	58.85
TK-10	110.88
UO-31	73.41
Prostate Cancer	
PC-3	67.59
DU-145	86.27
Breast Cancer	
MCF7	80.67
MDA-MB-231/ATCC	98.53
HS 578T	91.74
BT-549	125.91
T-47D	60.92
MDA-MB-468	103.16
Mean	90.51
Delta	31.66
Range	67.06

Mean Growth Percent - Growth Percent

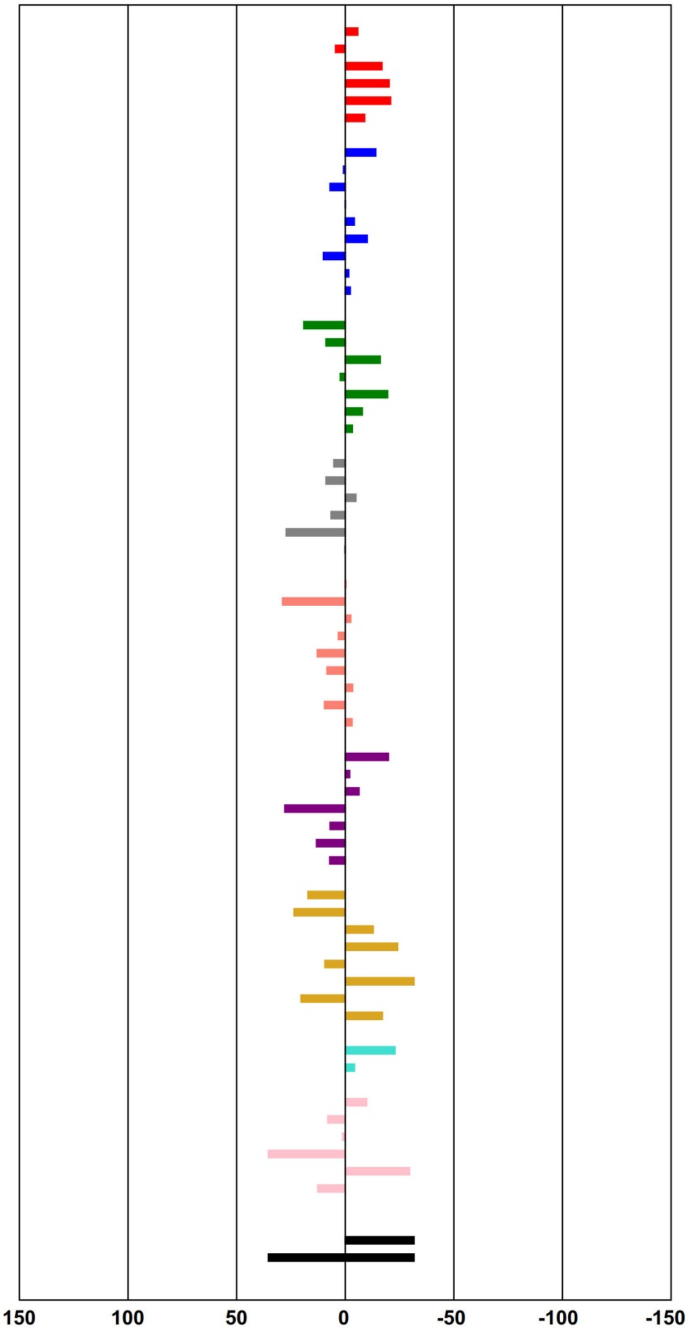

Figure S19. Anticancer screening data of compound **4** at single dose assay

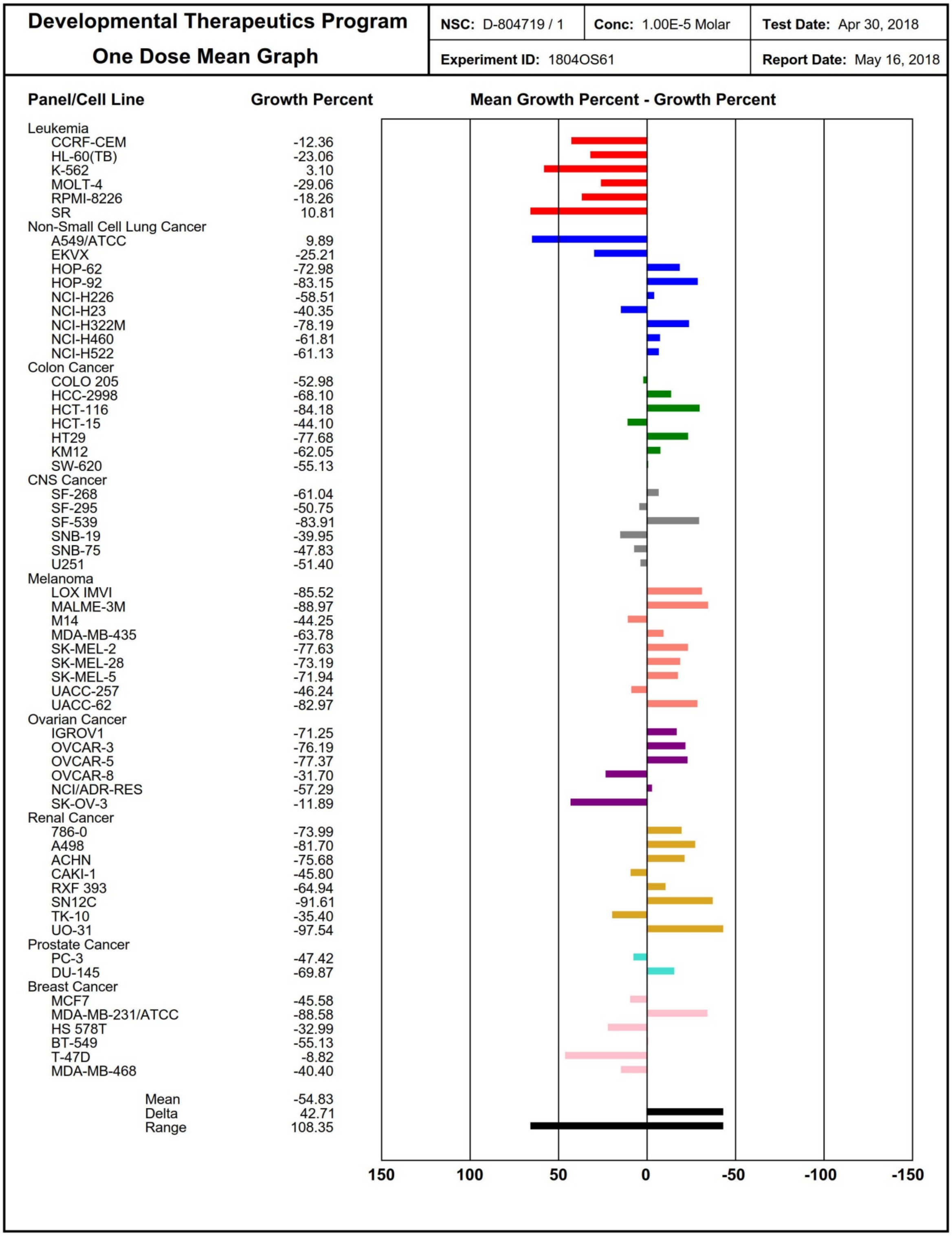

Figure S20. Anticancer screening data of compound **4** at a 5-dose assay

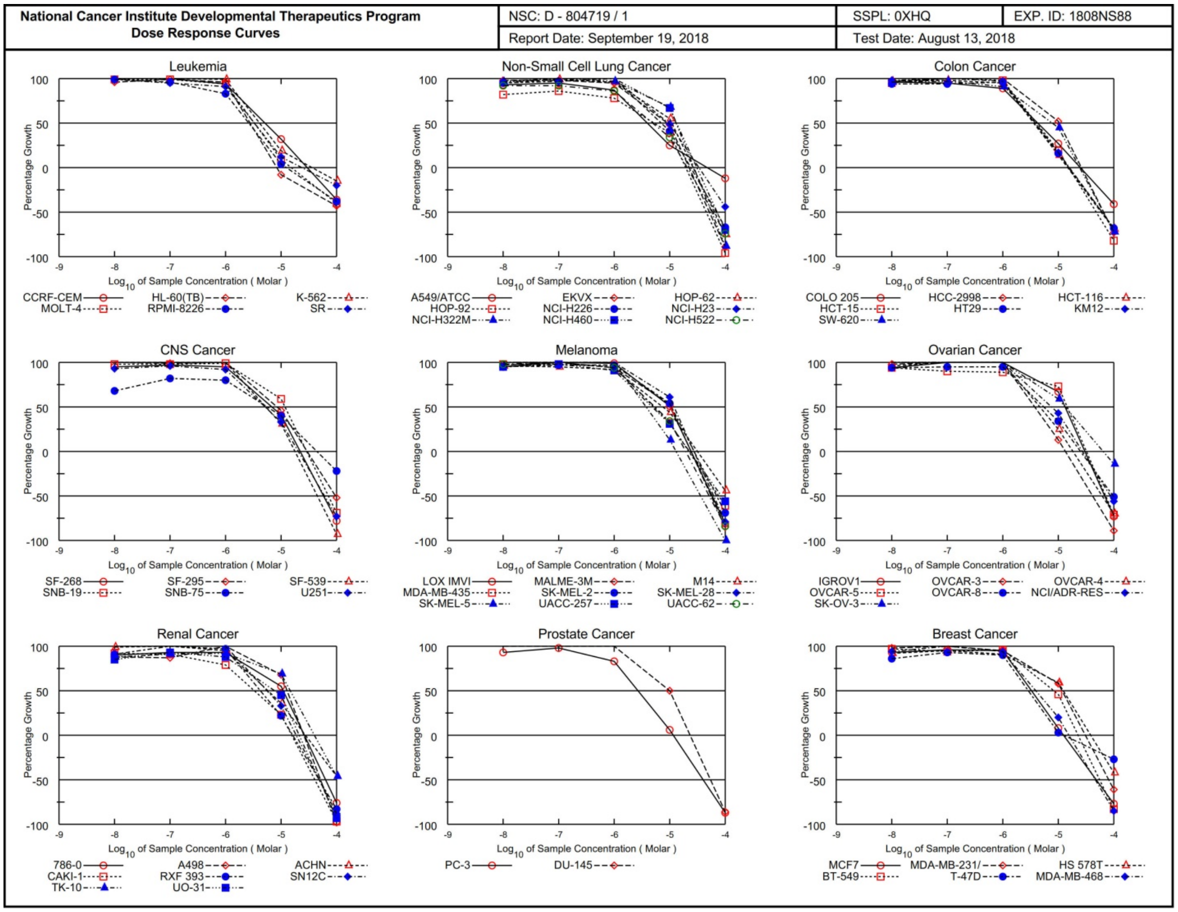

Figure S21. Anticancer screening data of compound **5** at single dose assay

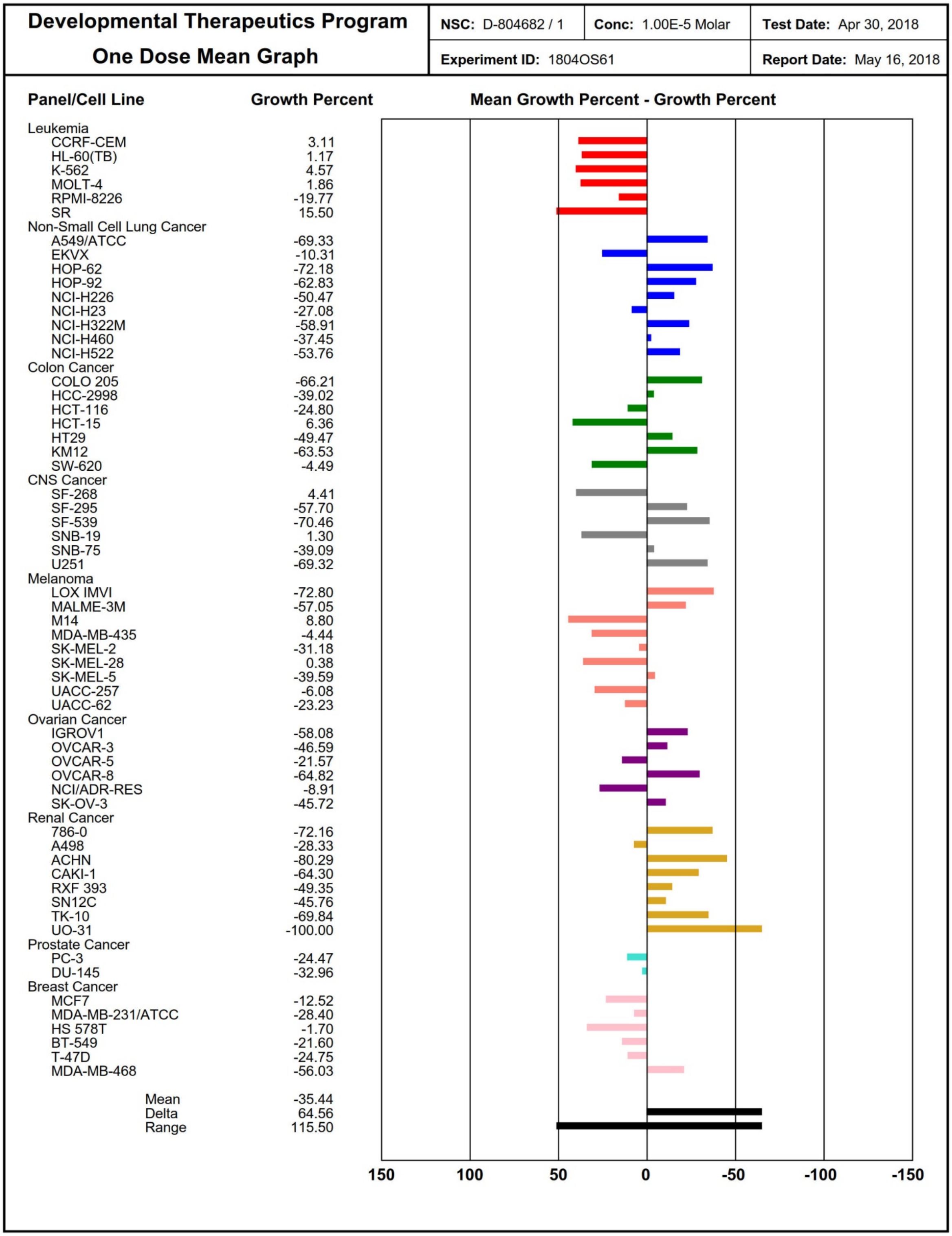

Figure S22. Anticancer screening data of compound **5** at a 5-dose assay

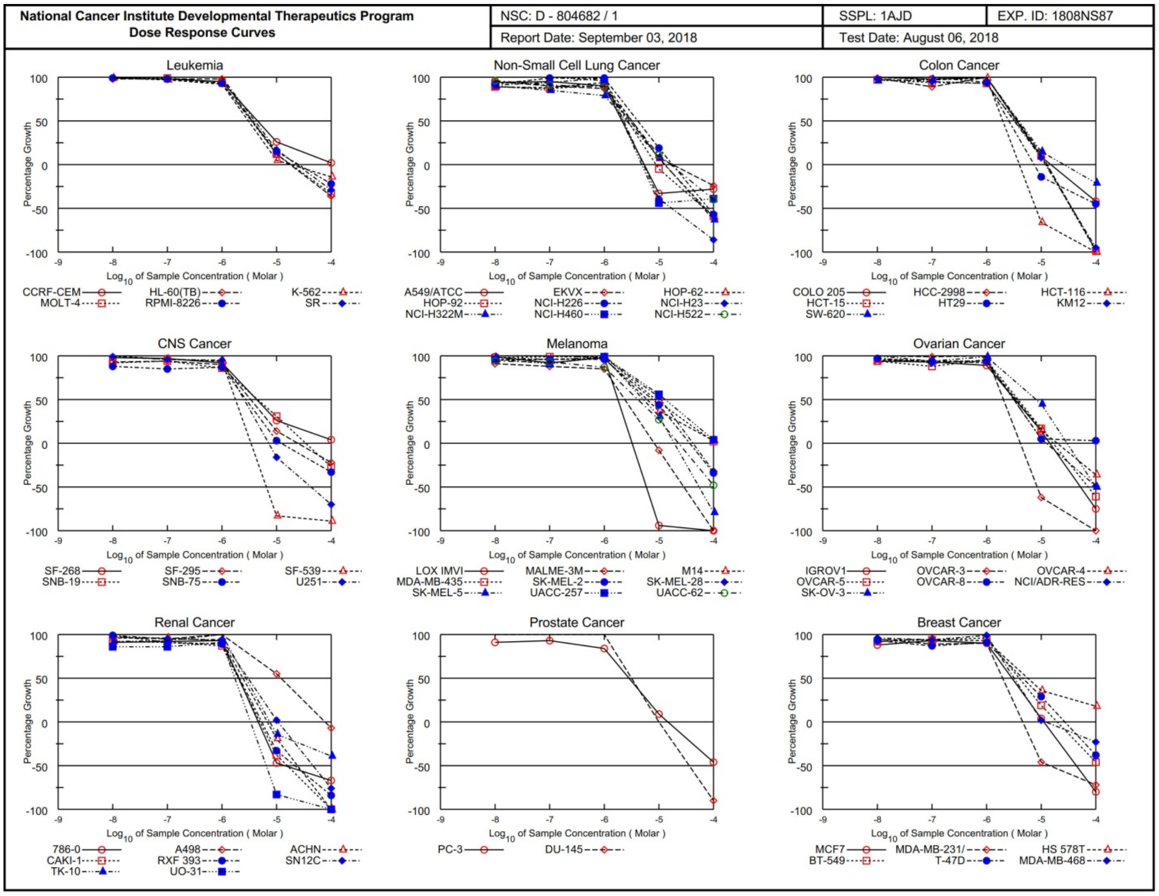

Figure S23. Anticancer screening data of compound **6** at single dose assay

Developmental Therapeutics Program One Dose Mean Graph	NSC: D-804742 / 1	Conc: 1.00E-5 Molar	Test Date: Apr 30, 2018
	Experiment ID: 1804OS61		Report Date: May 16, 2018

Panel/Cell Line	Growth Percent
Leukemia	
CCRF-CEM	5.18
HL-60(TB)	-23.98
K-562	-15.78
MOLT-4	1.18
RPMI-8226	-8.15
SR	-4.72
Non-Small Cell Lung Cancer	
A549/ATCC	-64.63
EKVX	-11.36
HOP-62	-69.12
HOP-92	-65.35
NCI-H226	-23.56
NCI-H23	-51.70
NCI-H322M	-83.84
NCI-H460	-100.00
NCI-H522	-57.83
Colon Cancer	
COLO 205	-19.47
HCC-2998	-90.36
HCT-116	-26.53
HCT-15	-24.81
HT29	-39.56
KM12	-52.23
SW-620	-86.66
CNS Cancer	
SF-268	-30.99
SF-295	-84.72
SF-539	-100.00
SNB-19	-74.52
SNB-75	-79.32
U251	-71.65
Melanoma	
LOX IMVI	-41.03
MALME-3M	-100.00
M14	-30.98
MDA-MB-435	-89.21
SK-MEL-2	-65.39
SK-MEL-28	-98.68
SK-MEL-5	-90.10
UACC-257	-39.58
UACC-62	-100.00
Ovarian Cancer	
IGROV1	-81.43
OVCAR-3	-66.02
OVCAR-5	-83.37
OVCAR-8	-12.90
NCI/ADR-RES	-13.05
SK-OV-3	-14.17
Renal Cancer	
786-0	-83.51
A498	-32.64
ACHN	-100.00
CAKI-1	-93.59
RXF 393	-82.88
SN12C	-53.68
TK-10	-67.30
UO-31	-100.00
Prostate Cancer	
PC-3	1.07
DU-145	-8.34
Breast Cancer	
MCF7	-60.54
MDA-MB-231/ATCC	-56.94
HS 578T	-23.26
BT-549	-51.94
T-47D	-32.56
MDA-MB-468	-48.60
Mean	-53.82
Delta	46.18
Range	105.18

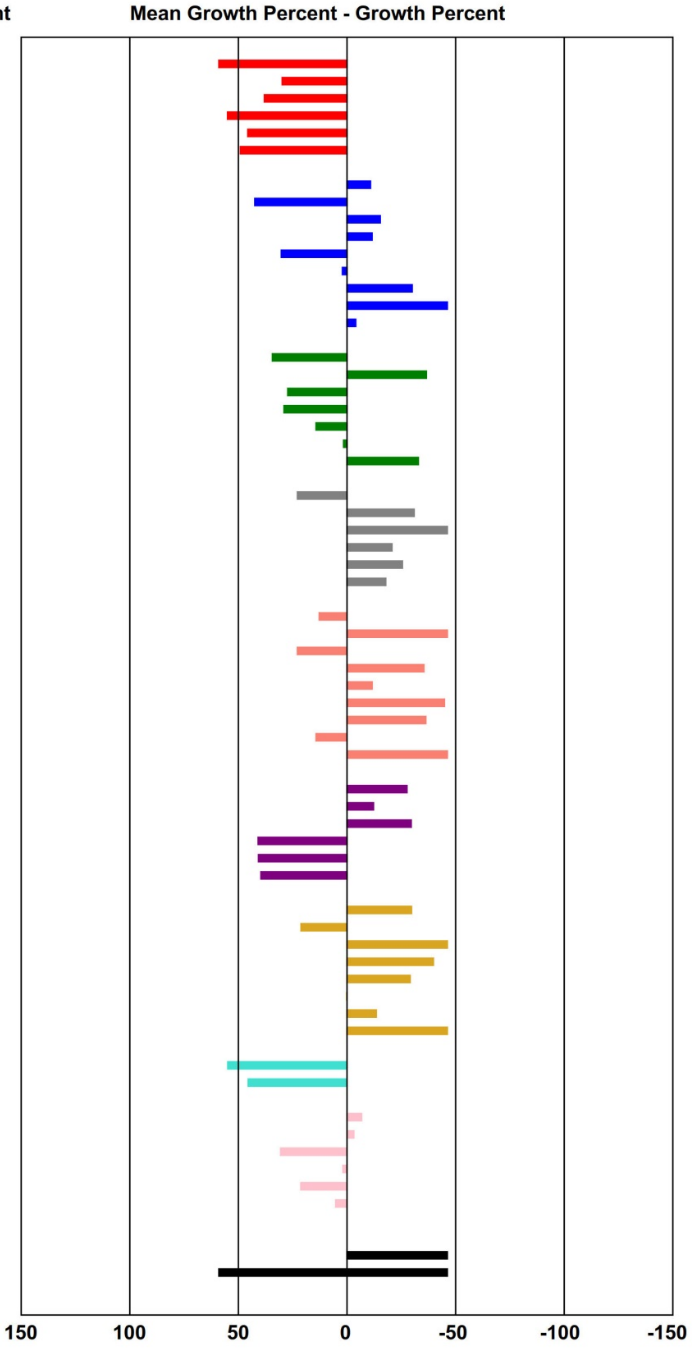

Figure S24. Anticancer screening data of compound **6** at a 5-dose assay

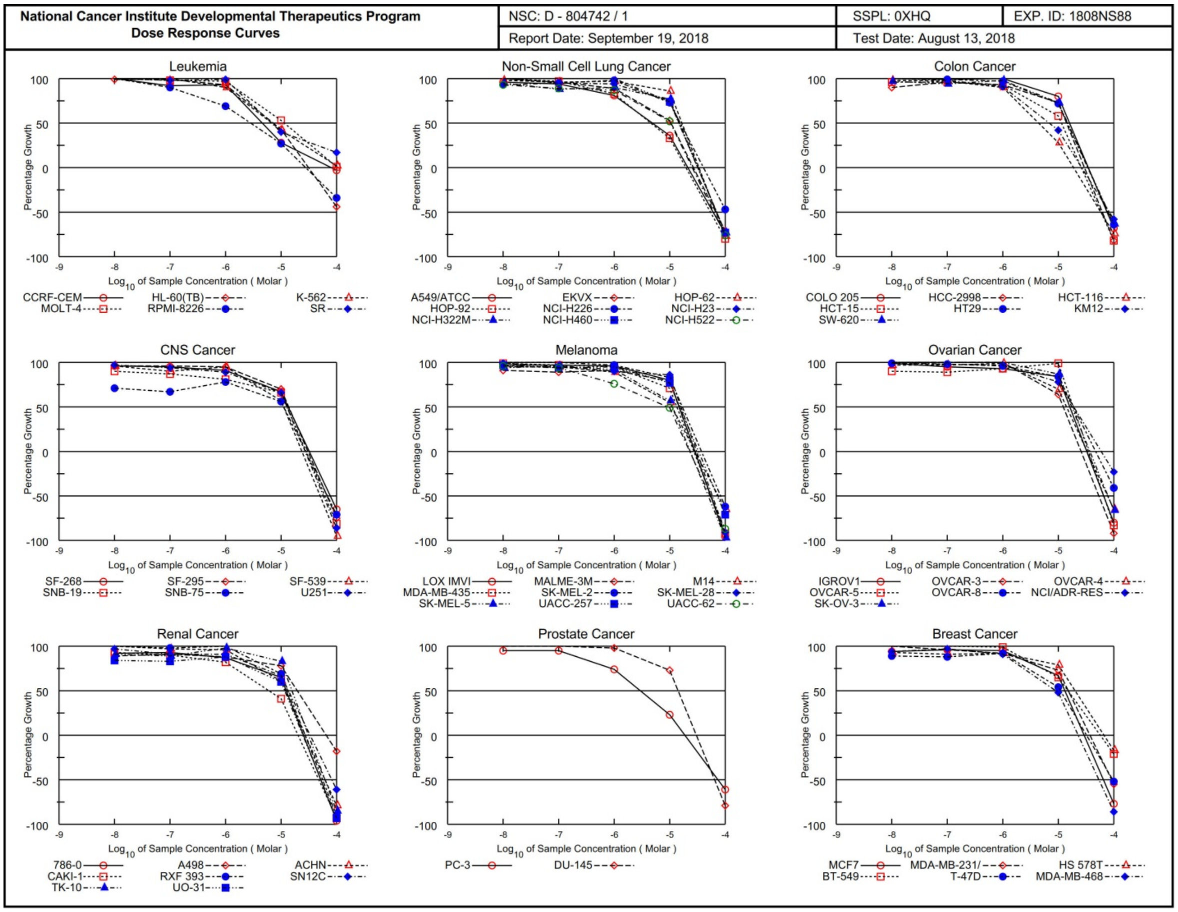

Figure S25. Anticancer screening data of compound **10** at single dose assay

Developmental Therapeutics Program One Dose Mean Graph	NSC: D-804723 / 1	Conc: 1.00E-5 Molar	Test Date: Apr 30, 2018
	Experiment ID: 1804OS61		Report Date: May 16, 2018

Panel/Cell Line	Growth Percent
Leukemia	
CCRF-CEM	1.39
HL-60(TB)	1.25
K-562	1.10
MOLT-4	2.76
RPMI-8226	-6.66
SR	23.66
Non-Small Cell Lung Cancer	
A549/ATCC	-4.70
EKVX	-15.68
HOP-62	-26.80
HOP-92	-55.49
NCI-H226	36.64
NCI-H23	-8.47
NCI-H322M	-68.83
NCI-H460	-43.19
NCI-H522	-16.98
Colon Cancer	
COLO 205	7.82
HCC-2998	-23.40
HCT-116	1.97
HCT-15	-22.06
HT29	3.90
KM12	-25.31
SW-620	-36.18
CNS Cancer	
SF-268	-16.88
SF-295	-26.39
SF-539	-36.20
SNB-19	-29.49
SNB-75	-51.56
U251	-13.77
Melanoma	
LOX IMVI	1.37
MALME-3M	-18.76
M14	5.07
MDA-MB-435	-32.41
SK-MEL-2	-24.21
SK-MEL-28	-31.42
SK-MEL-5	-23.93
UACC-257	-46.67
UACC-62	-22.62
Ovarian Cancer	
IGROV1	-6.94
OVCAR-3	-45.89
OVCAR-5	-47.68
OVCAR-8	-3.81
NCI/ADR-RES	-9.52
SK-OV-3	-27.22
Renal Cancer	
786-0	-22.61
A498	-44.82
ACHN	-49.24
CAKI-1	-54.52
RXF 393	-42.28
SN12C	-34.74
TK-10	-32.23
UO-31	-34.32
Prostate Cancer	
PC-3	-53.78
DU-145	-100.00
Breast Cancer	
MCF7	-8.46
MDA-MB-231/ATCC	-47.78
HS 578T	-4.98
BT-549	-2.14
T-47D	-12.76
MDA-MB-468	-19.87
Mean	-22.83
Delta	77.17
Range	136.64

Mean Growth Percent - Growth Percent

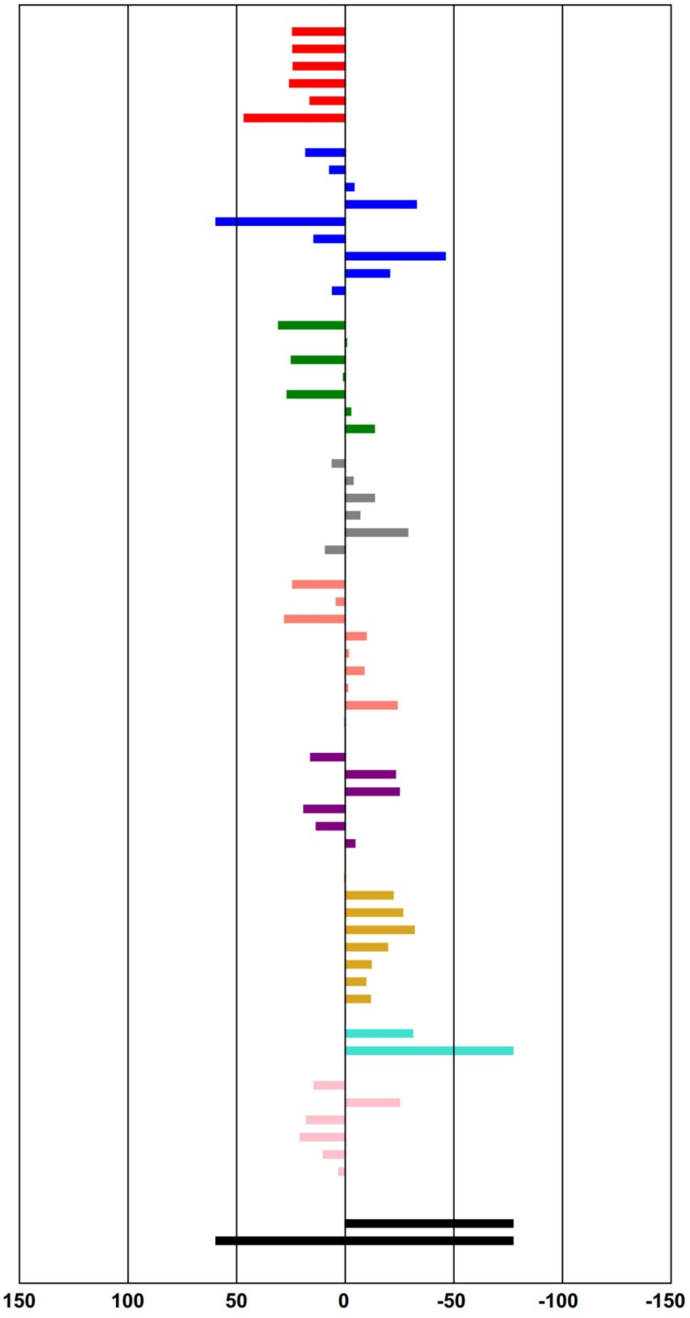

Figure S26. Anticancer screening data of compound **10** at a 5-dose assay

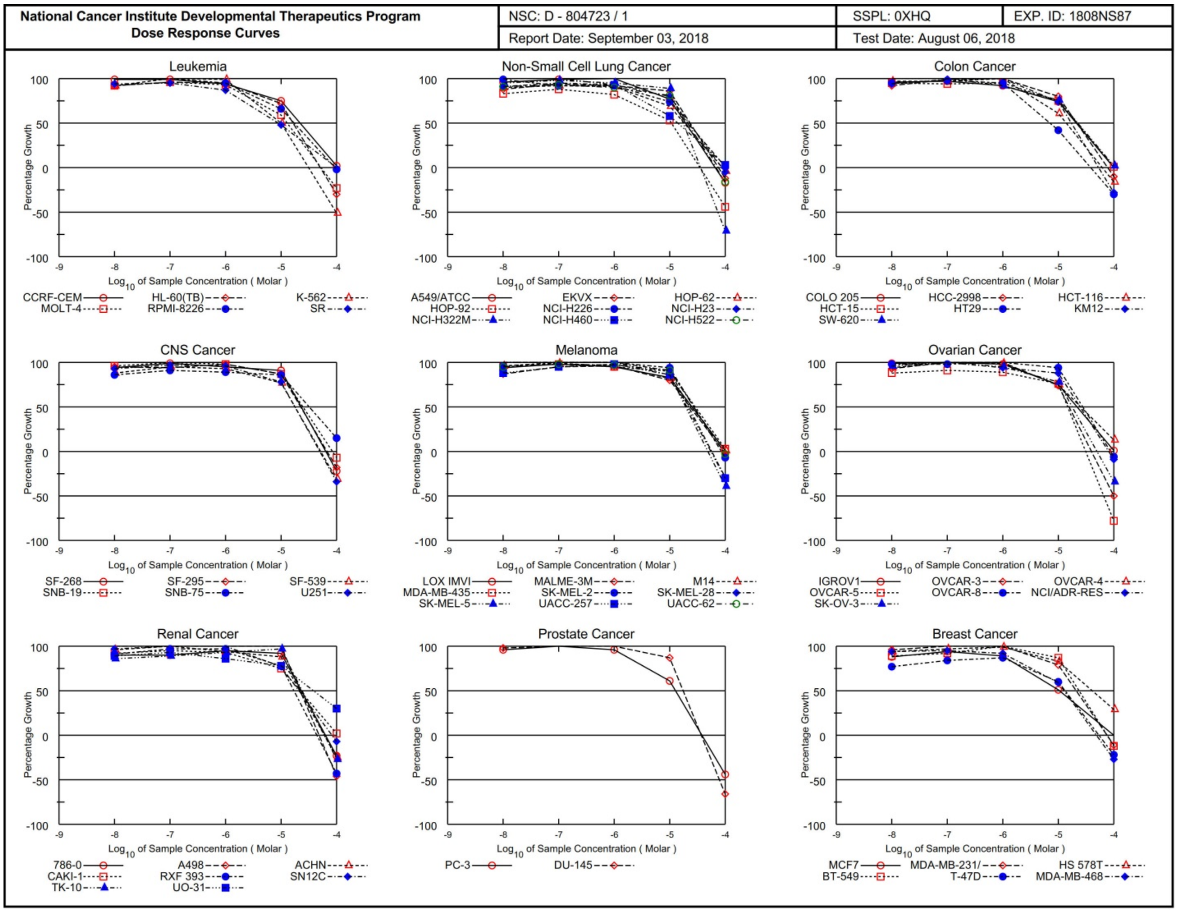

Figure S27. Anticancer screening data of compound **11** at single dose assay

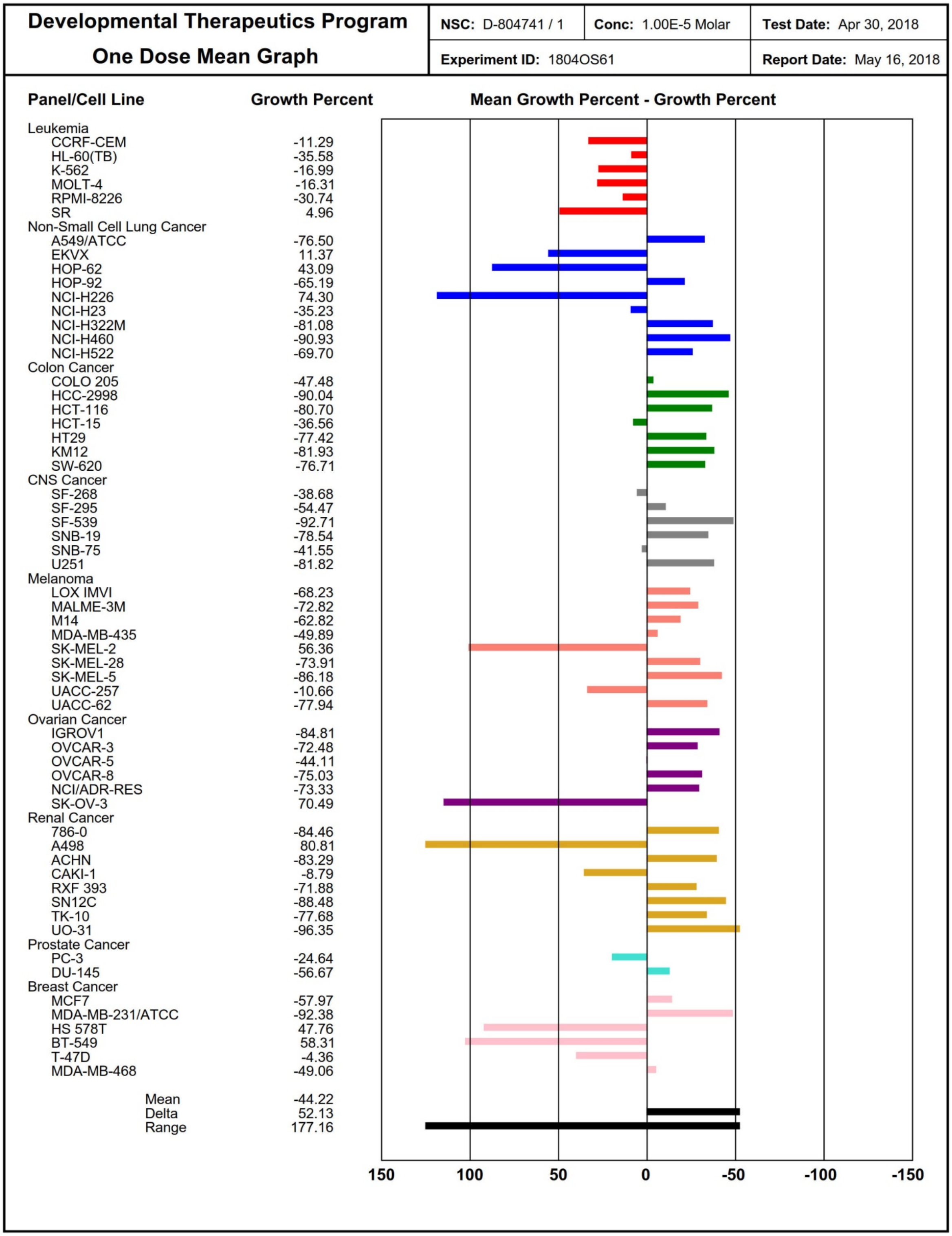

Figure S28. Anticancer screening data of compound **11** at a 5-dose assay

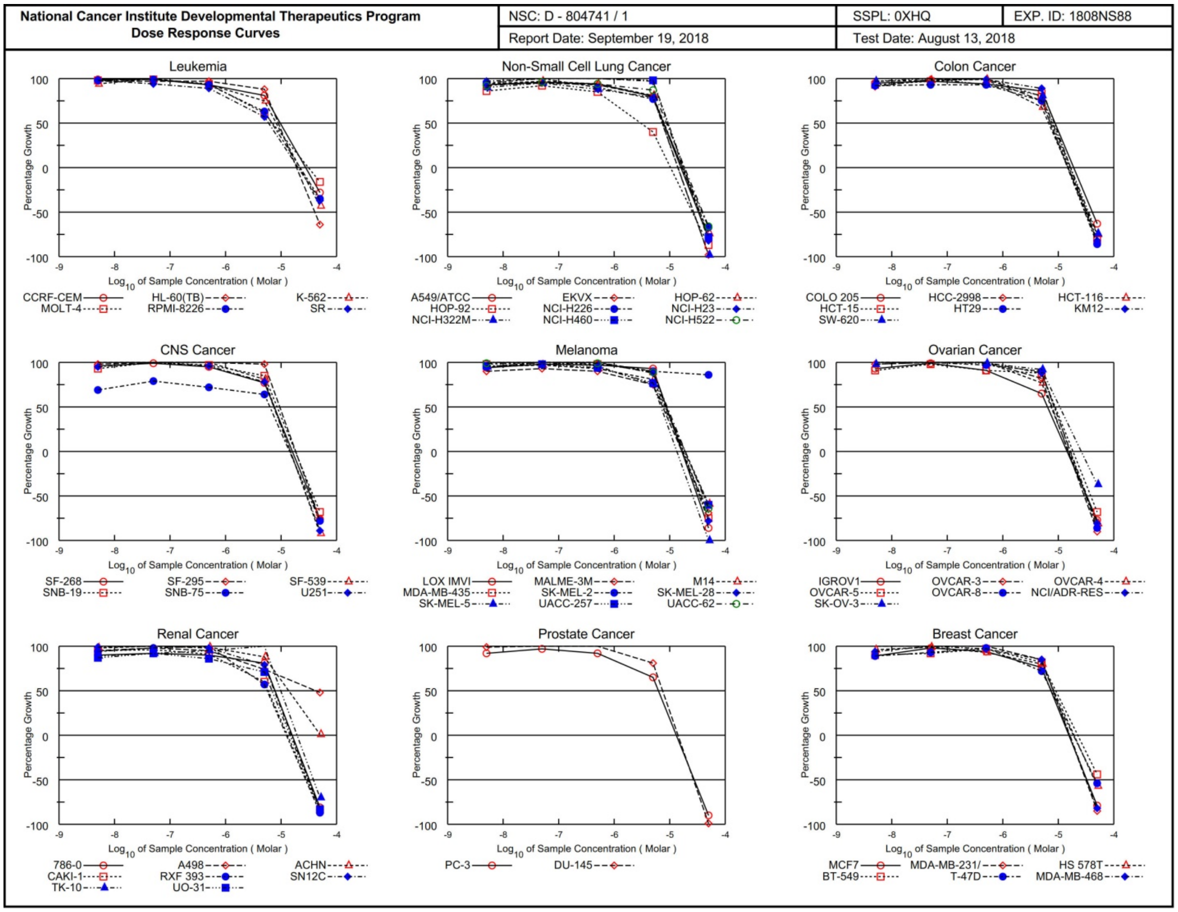

Figure S29. Anticancer screening data of compound **21** at single dose assay

Developmental Therapeutics Program	NSC: D-804737 / 1	Conc: 1.00E-5 Molar	Test Date: Apr 30, 2018
One Dose Mean Graph	Experiment ID: 1804OS61		Report Date: May 16, 2018

Panel/Cell Line	Growth Percent
Leukemia	
CCRF-CEM	7.42
HL-60(TB)	0.90
K-562	5.42
MOLT-4	-26.81
RPMI-8226	3.28
SR	1.86
Non-Small Cell Lung Cancer	
A549/ATCC	15.82
EKVX	53.13
HOP-62	32.54
HOP-92	-14.95
NCI-H226	2.42
NCI-H23	21.79
NCI-H322M	46.12
NCI-H460	4.23
NCI-H522	13.36
Colon Cancer	
COLO 205	17.60
HCC-2998	26.92
HCT-116	4.16
HCT-15	34.28
HT29	3.88
KM12	10.89
SW-620	21.96
CNS Cancer	
SF-268	29.14
SF-295	36.56
SF-539	-2.29
SNB-19	28.93
SNB-75	18.44
U251	-39.66
Melanoma	
LOX IMVI	14.56
MALME-3M	25.27
M14	26.57
MDA-MB-435	14.55
SK-MEL-2	36.23
SK-MEL-28	29.25
SK-MEL-5	-84.68
UACC-257	8.82
UACC-62	-25.76
Ovarian Cancer	
IGROV1	18.78
OVCAR-3	-34.55
OVCAR-5	50.59
OVCAR-8	13.17
NCI/ADR-RES	17.06
SK-OV-3	34.60
Renal Cancer	
786-0	-11.47
A498	-0.38
ACHN	13.94
CAKI-1	18.52
RXF 393	-43.14
SN12C	-71.91
TK-10	37.88
UO-31	19.03
Prostate Cancer	
PC-3	4.49
DU-145	27.53
Breast Cancer	
MCF7	12.44
MDA-MB-231/ATCC	5.09
HS 578T	24.26
BT-549	4.40
T-47D	15.11
MDA-MB-468	4.41
Mean	9.53
Delta	94.21
Range	137.81

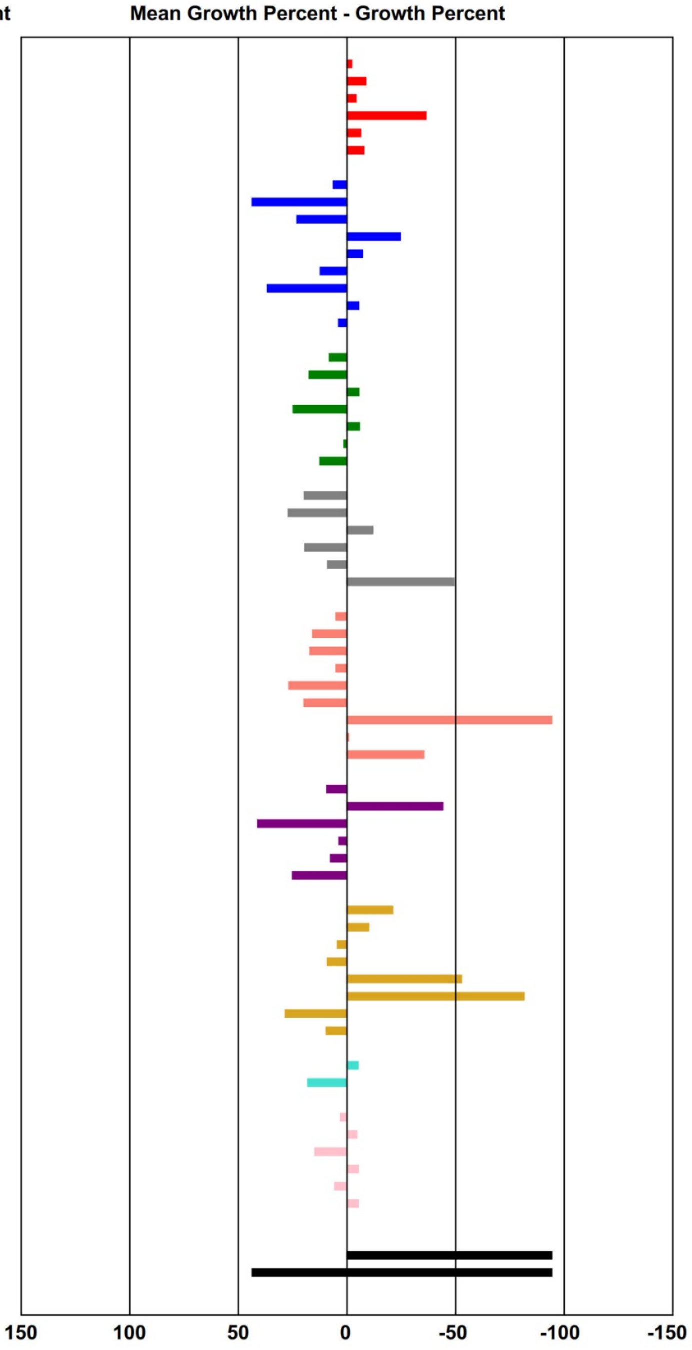

Figure S30. Anticancer screening data of compound **21** at a 5-dose assay

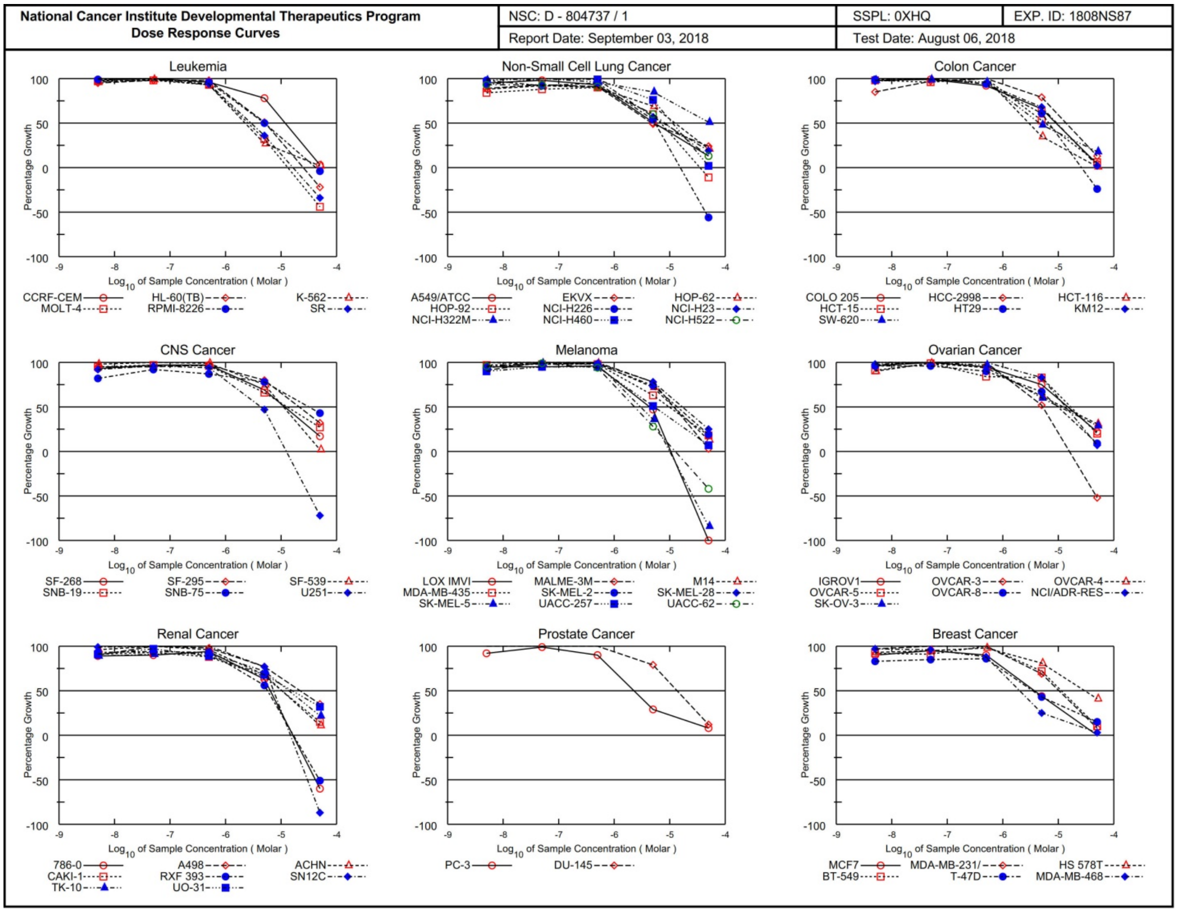

Table S1. Gene ontology (GO) term enrichment analysis for compound **4** across the NCI-60 panel cell lines based on CellMiner analysis

**GO Term**	***P* value**	**Adjusted *P* value**[1]	**Genes**
**Biological process**			
–	–	–	–
**Molecular function**			
–	–	–	–

[1] *P* value was calculated using the hypergeometric test and corrected for multiple hypothesis testing (*P* <0.05) using the Benjamini-Hochberg false discovery rate (FDR) adjustment.

Table S2. Gene ontology (GO) term enrichment analysis for compound **5** across the NCI-60 panel cell lines based on CellMiner analysis

GO Term	*P* value	Adjusted *P* value	Genes
**Biological process**			
Central nervous system development	4.3374E-5	3.5581E-2	RPH3A EMX1 CYP26C1 TLX3 GSX1 HP TBR1 LHX5 PTF1A
Hindbrain development	7.1163E-5	3.5581E-2	HP TBR1 LHX5 PTF1A
Brain development	1.5166E-4	4.2809E-2	RPH3A EMX1 GSX1 HP TBR1 LHX5 PTF1A
Multicellular organismal process	1.8287E-4	4.2809E-2	TDPX2 HP TNFAIP2 OR1I1 CACNA1F BRS3 RPH3A RS1 PLAU OBP2B MDK PRDX1 LTA4H DRD4 EMX1 PAQR7 GSX1 OR6J1 LIF TBR1 MMP9 EREG SLC6A5 F7 OR13C8 CYP26C1 TLX3 LHX5 SCN4A PTF1A
Neuron differentiation	2.6561E-4	4.2809E-2	EMX1 TLX3 GSX1 LIF TBR1 LHX5 CACNA1F PTF1A
Removal of superoxide radicals	4.0054E-4	4.2809E-2	TDPX2 PRDX1
Cellular response to superoxide	4.0054E-4	4.2809E-2	TDPX2 PRDX1
Cellular response to oxygen radical	4.0054E-4	4.2809E-2	TDPX2 PRDX1
System development	4.1848E-4	4.2809E-2	EMX1 GSX1 TDPX2 HP LIF TNFAIP2 TBR1 CACNA1F MMP9 EREG RPH3A F7 CYP26C1 TLX3 PLAU MDK PRDX1 LHX5 LTA4H PTF1A
Neuron fate commitment	4.4580E-4	4.2809E-2	TLX3 GSX1 PTF1A
Natural killer cell mediated immunity	5.1371E-4	4.2809E-2	TDPX2 PRDX1
Natural killer cell mediated cytotoxicity	5.1371E-4	4.2809E-2	TDPX2 PRDX1
**Molecular function**			
Thioredoxin peroxidase activity	9.7772E-5	2.4736E-2	TDPX2 PRDX1

[1] *P* value was calculated using the hypergeometric test and corrected for multiple hypothesis testing (*P* <0.05) using the Benjamini-Hochberg false discovery rate (FDR) adjustment.

Table S3. Gene ontology (GO) term enrichment analysis for compound **6** across the NCI-60 panel cell lines based on CellMiner analysis

**GO Term**	***P* value**	**Adjusted *P* value**	**Genes**
**Biological process**			
Immune system process	7.4114E-8	1.4037E-4	CX3CR1 CD86 FH CXCL9 SELPLG TNFRSF13B NCF4 WAS IKZF1 ITGAL IL2RG LY9 CORO1A CTSS CD79A IGKC FLVCR1 IL21R CCR7 S1PR4 CCR2 CCL25 CR2 LY86 THEMIS RHOH CYBB DEFA1 HSH2D POLR3A CD8B IFNG BANK1 KLHL6 POU2AF1 PECAM1 CD28 TLR9 HCLS1 TLR7 DTL MS4A1 PVALB
Positive regulation of immune system process	3.0981E-6	2.9339E-3	CD86 FH CR2 THEMIS MIA3 IKZF1 CORO1A FCER2 CD79A IFNG KLHL6 CD28 TLR9 CD38 CD37 TLR7 DTL MS4A1
Immune response	6.4668E-6	4.0827E-3	CX3CR1 CD86 FH CXCL9 NCF4 WAS ITGAL IL2RG LY9 CORO1A CTSS IGKC CCR7 S1PR4 CCR2 CCL25 CR2 LY86 CYBB DEFA1 POLR3A CD8B KLHL6 POU2AF1 CD28 TLR9 TLR7 DTL MS4A1
Leukocyte activation	3.8908E-5	1.8423E-2	CX3CR1 CD86 WAS THEMIS RHOH IKZF1 ITGAL CD79A HSH2D CD8B BANK1 IL21R CD28 TLR7 MS4A1
Lymphocyte activation	5.5271E-5	1.8927E-2	CD86 WAS THEMIS RHOH IKZF1 ITGAL CD79A HSH2D CD8B BANK1 IL21R CD28 MS4A1
Regulation of immune system process	5.9959E-5	1.8927E-2	CD86 FH CR2 SPI1 TNFRSF13B THEMIS MIA3 IKZF1 CORO1A FCER2 CD79A CD8B IFNG KLHL6 CD28 TLR9 CD38 CD37 TLR7 DTL MS4A1
Defense response	1.2927E-4	3.4978E-2	CX3CR1 CD86 FH CXCL9 WAS LSP1 ITGAL PLA2G7 CORO1A CYSLTR1 CCR7 CCR3 CCR2 CCL25 CR2 RNASE6 LY86 CYBB KIR3DL2 DEFA1 POLR3A IFNG TLR9 CARD18 TLR7 MS4A1
Regulation of immune response	1.6038E-4	3.7970E-2	CD86 FH CR2 THEMIS FCER2 CD79A CD8B IFNG KLHL6 CD28 CD37 TLR7 DTL MS4A1
**Molecular function**			
Cytokine binding	4.4139E-5	2.4850E-2	CX3CR1 IL10RA TNFRSF18 IL3RA IL21R TNFRSF17 CCR7 IL2RG CCR3 CCR2

[1] *P* value was calculated using the hypergeometric test and corrected for multiple hypothesis testing ($P < 0.05$) using the Benjamini-Hochberg false discovery rate (FDR) adjustment.

Table S4. Gene ontology (GO) term enrichment analysis for compound **10** across the NCI-60 panel cell lines based on CellMiner analysis

**GO Term**	***P* value**	**Adjusted *P* value**	**Genes**
**Biological process**			
Chromatin organization	2.4288E-6	2.4791E-3	FOXA1 KMT2D SMARCD2 KDM3B KMT2B HIST2H2AB YEATS4 HIST2H2AC MBTD1 HIRA VPS72 LEO1 HIST1H2AE SUDS3 ATXN7L3 HIST1H3D BPTF HIST1H2AM SETDB1 KMT5C USP21 SETD1B INO80 HIST2H2BE HIST2H2BD HIST3H2A TADA1 TSSK4 HIST2H3D HIST1H2BC
Positive regulation of steroid hormone receptor signaling pathway	3.1487E-6	2.4791E-3	FOXA1 KMT2D KMT2B RAC3
Positive regulation of estrogen receptor signaling pathway	3.1487E-6	2.4791E-3	FOXA1 KMT2D KMT2B RAC3
Regulation of estrogen receptor signaling pathway	4.1174E-5	2.2425E-2	FOXA1 KMT2D KMT2B RAC3
Protein-DNA complex assembly	4.7470E-5	2.2425E-2	HIST1H2AM HIST3H2A HIST2H2AB HIST1H2AE HIST2H3D HIST2H2BE HIST1H3D HIST2H2AC HIST2H2BD PIAS1 HIST1H2BC
Nucleosome assembly	9.5898E-5	3.7752E-2	HIST1H2AM HIST3H2A HIST2H2AB HIST1H2AE HIST2H3D HIST2H2BE HIST1H3D HIST2H2AC HIST2H2BD HIST1H2BC
Chromatin assembly	1.4443E-4	4.8735E-2	HIST1H2AM HIST3H2A HIST2H2AB HIST1H2AE HIST2H3D HIST2H2BE HIST1H3D HIST2H2AC HIST2H2BD HIST1H2BC
**Molecular function**			
Binding	1.9603E-5	1.0632E-2	TCERG1 ZNF296 EHF IFITM1 IL1RN NUP107 PGAP2 ACSM3 RPL34 PRF1 CALML4 AQP5 NADSYN1 RPL6 CRKL CCAR1 IKZF5 ZFYVE28 FAM110A PNP RUSC1 RASSF5 CHEK2 MYB OGFOD2 TRIM25 STRA13 KIF21B ZNF721 LGALS9 ZNF720 LGALS8 ATXN7L3 FNBP4 IL15RA AQR ENTPD2 NUP210 RSAD2 SYTL1 COG2 ACSL5 PHKA1 SIGIRR CLDN4 SPINT1 PRKAR1B PPA2 PPA1 ZC3H11A TRIM14 TARS2 DNA2 ATF5 ZNF830 HIST2H3D ZSCAN29 CDS1 CRABP2 GIPR SH2D2A PEX11B STXBP2 GATA2 SAMSN1 ACTR3B MTMR7 MBTD1 APOH INPP5D RDH16 CEP72 GSE1 RIMKLA ZBTB7A NTSR1 HIST1H3D ZRANB3 JUP SETDB1 LRBA SETD1B SIAH2 SH2D1B ABCA7 NBEAL2 PTK6 PYCR2 INO80 TRERF1 UBAC1 GADD45G CDAN1 RPL27A CCDC88C MYO5C MKS1 INTS5 REN DUS1L

			ZNF138 RPS24 ZNF497 RET DHRS13 SERPINA1 HIST2H2AB ICA1 NAT10 HBB SMG7 PCSK6 ZNF48 HIST2H2AC MMP20 DMBX1 COIL GYS2 TRMT112 ENSA RAC3 JAK3 ZNF485 TIGD3 SERPINB3 UPF2 SERPINB4 PARP4 SIRT7 HEXDC CBFA2T3 SRP9 HIST2H2BE F5 HIST2H2BD MED28 FAHD2B VAMP8 CNKSR1 PSMA3 PDIK1L RPUSD2 DMC1 TUT1 ALDOC GLCCI1 RNF166 DCP2 PDZRN4 ZNF592 RPL10 MRPS31 TUBD1 TANK AKAP1 FGD3 HIRA ADRBK1 LEO1 PLAGL2 RPS2 RPL18 REM2 SPDEF GCH1 F12 CARD9 OSM NR1H3 KLHL3 PUS7L GRHL1 C17ORF62 GRHL2 DEF6 BATF MAS1 TMCO6 KRT19 LRG1 NR6A1 HIST3H2A DHRS9 TTC6 IL17F KBTBD7 FOXA1 DGKE TOR2A ZNF692 STEAP4 PRUNE RORC YEATS4 TSEN2 NPEPPS ZFP36 CAPN8 PPIP5K1 EME2 POGZ ZNF207 HIST1H2AE TNFRSF8 ZNF687 HIST1H2AM ZBP1 TLE3 ZBTB39 CISH KMT5C GTF3A ZBTB32 LMTK3 ANXA10 LEMD3 VRK3 DGKZ PIAS1 PYGO2 POLG2 MADD LY6D CARD17 CMPK2 S100A4 ZNF678 MAPKAPK5 ELMO3 TSSK4 PKP3 SNRPF SRSF5 MZF1 SLC27A2 SNRPA ABCG1 ANAPC2 FKBP5 DAP3 HIST1H2BC KMT2D ZNF792 RBM8A KMT2B COX17 ALOX15 ISG20L2 DAPP1 KLHL12 GLB1L2 ARL17B SLC9A3R1 CACNA1I SOCS1 CALCR THPO BAG1 RHPN1 EIF4EBP2 E4F1 SUDS3 ZNF786 MARK2 BPTF NEK8 NOP14 KLHDC4 CPSF6 ZG16B KLHL26 PHF11 TNFRSF10A ARHGAP27 TDRD6 CORO2A WASH3P DNAJC17 ANKHD1 CLK2 CXCL11 PTPRC DLG3 HOOK2 ZFP62 SP1 ALB SP6 TADA1 MDM4 ZCCHC2 SCYL3 B4GALT3 MAST4 ZMYND19 ADAR PIK3CG OASL CALB2 RBM4 SDR16C5 MRPL1 AP1G2 VPS72 ENOSF1 SENP8 CA4 DGCR8 ZNF524 GET4 PLA2G12A SH2D3A DCXR DDX51 ARFGAP2 ETV7 SLC25A18 HSH2D ESRP2 AGAP5 IRF7 LUC7L3 SLC25A10 TRIM59 CDHR5 TOP1 LOC440434 FBP1 SMARCD2 CATSPER3 CATSPER2 TRIM52 KDM3B DDX23 ASB13 ZBTB42 NOD1 NOD2 JAKMIP1 TOB1 C1ORF94 MLLT6 PRRG4 ZNRF1 AP3M1 GNA14 CLEC3B ZNRF2 IFNL2 PARD6A IFNL1 RAB26 TDG RNF39 SNCG PTK2B PCBD2 CAMK2G RBM38 USP21 CDC42BPG RPL23A YY1AP1 USF1 BAIAP3 MAPK13 POLR3A SLC25A39 TEC RPS6KB1 DNAJA4 SYT10 POLE2 RBM41 GALM NAA16 S100P PTPN7 PIK3AP1 TAF4 TPT1
Nucleic acid binding	3.0997E-5	1.0632E-2	FOXA1 ZNF296 EHF ZNF692 RPL34 RORC PRF1 YEATS4 TSEN2 RPL6 CCAR1 IKZF5 ZFP36 EME2 MYB POGZ ZNF207 HIST1H2AE TRIM25 STRA13 ZNF721 ZNF687 ZNF720 HIST1H2AM ZBP1

			ZBTB39 AQR GTF3A ZBTB32 LEMD3 PIAS1 POLG2 ZC3H11A ZNF678 SNRPF SRSF5 DNA2 ATF5 HIST2H3D MZF1 SNRPA ZSCAN29 HIST1H2BC KMT2D ZNF792 RBM8A KMT2B ISG20L2 GATA2 E4F1 ZNF786 ZBTB7A HIST1H3D BPTF ZRANB3 NOP14 SETDB1 SETD1B INO80 TDRD6 TRERF1 DNAJC17 ANKHD1 ZFP62 SP1 RPL27A ALB SP6 ZNF138 ZCCHC2 ZNF497 HIST2H2AB ADAR ZNF48 HIST2H2AC OASL DMBX1 RBM4 MRPL1 VPS72 DGCR8 ZNF524 ZNF485 TIGD3 UPF2 PARP4 DDX51 CBFA2T3 SRP9 HIST2H2BE HIST2H2BD ETV7 ESRP2 RPUSD2 DMC1 IRF7 TUT1 LUC7L3 TOP1 DCP2 ZNF592 RPL10 DDX23 ZBTB42 JAKMIP1 AKAP1 HIRA TDG PLAGL2 RPS2 RPL18 SPDEF RBM38 NR1H3 RPL23A PUS7L GRHL1 GRHL2 USF1 BATF POLR3A NR6A1 HIST3H2A TEC POLE2 RBM41 TAF4
Inorganic diphosphatase activity	8.7375E-5	1.9980E-2	PPA2 PPA1 PRUNE

[1] *P* value was calculated using the hypergeometric test and corrected for multiple hypothesis testing (*P* <0.05) using the Benjamini-Hochberg false discovery rate (FDR) adjustment.

Table S5. Gene ontology (GO) term enrichment analysis for compound **11** across the NCI-60 panel cell lines based on CellMiner analysis

GO Term	*P* value	Adjusted *P* value	Genes
**Biological process**			
–	–	–	–
**Molecular function**			
Histone binding	8.2053E-5	5.5048E-3	CHD8 PKN1 PHF8
Methylated histone residue binding	8.2779E-5	5.5048E-3	CHD8 PHF8

[1] *P* value was calculated using the hypergeometric test and corrected for multiple hypothesis testing ($P < 0.05$) using the Benjamini-Hochberg false discovery rate (FDR) adjustment.

Table S6. Gene ontology (GO) term enrichment analysis for compound **21** across the NCI-60 panel cell lines based on CellMiner analysis

**Go Term**	***P* value**	**Adjusted *P* value**	**Genes**
**Biological process**			
Gene expression	2.4408E-30	4.4398E-27	CCNT1 RPL32 PDCD7 EIF4A3 RPL10L ARID4A TSEN2 RPL10A TSEN54 RPL6 CCAR1 RPS17 RPS13 PUS3 RPS9 AQR EIF1AX IMP3 METTL1 RPS5 PUS1 IMP4 POLRMT GTPBP3 MRPS18C TUFM LARS2 POLG2 CLNS1A TFAM PPIH TARS2 SNRPE SNRPF GEMIN8 DUS3L SRSF9 DDX5 MAX MRPL18 MRPL16 NOLC1 MRPL12 MRPL10 MRPL11 PDCD11 GAR1 EIF2B4 NOP14 CPSF6 PNPT1 INTS2 ECD PA2G4 MRPL21 RPS25 RPL27A INTS5 INTS4 MPHOSPH10 DUS1L HNRNPA1L2 RSL24D1 RPS24 CREBZF MRPS16 RPLP1 RPLP0 DDX41 MRPL32 MRPL4 SNAPC5 MRPL42 MRPL2 MRPL1 SART3 SNAPC4 MRPL9 PRMT5 TSFM MRPS25 MARS2 MRPL49 DDX54 DDX51 POLR1C PRIM1 DDX23 DDX21 CSTF2T MRPS30 EXOSC7 PDF TAF1C POLR2D C10ORF2 RPS2 RPS27A EIF4B SSB KRR1 RPL23A NPM3 PUS7L TRIT1 ADAT2 RSL1D1 EIF3M SPCS2 POLR3A POLR3B WT1 TRMT5 MARS EIF3J RPS6KB2 RPL13AP3 FAU RBMX METAP2
Translation	2.5008E-25	2.2745E-22	MRPS16 RPL32 RPLP1 RPLP0 RPL10L RPL10A MRPL32 RPL6 MRPL4 MRPL42 MRPL2 RPS17 MRPL1 MRPL9 RPS13 TSFM RPS9 EIF1AX IMP3 MRPS25 RPS5 MARS2 MRPL49 MRPS18C TUFM LARS2 POLG2 TARS2 MRPL18 MRPL16 MRPS30 MRPL12 MRPL10 MRPL11 PDF RPS2 RPS27A EIF4B EIF2B4 RPL23A MRPL21 RPS25 RSL1D1 EIF3M RPL27A MARS EIF3J RPS6KB2 RPL13AP3 FAU RSL24D1 RPS24
Cellular macromolecule metabolic process	1.3003E-18	7.8841E-16	CCNT1 RPL32 PDCD7 EIF4A3 RPL10L ARID4A YEATS4 TSEN2 RPL10A TSEN54 RPL6 CCAR1 NDST2 PPP1CC RPS17 HELB TRIM28 HERC1 KAT5 ALKBH1 RUVBL1 STRA13 FBXO6 PIM2 ATXN7L3 RPS13 MEN1 PUS3 RPS9 USP7 AQR ENDOG KMT5C EIF1AX IMP3 METTL1 RPS5 USP3 PUS1 COG2 IMP4 CHD1L POLRMT PHKA2 CLPX GTPBP3 MRPS18C TUFM LARS2 POLG2 CLNS1A TRIM13 TSSK6 TFAM PPIH TARS2 SNRPE SNRPF GEMIN8 FKBP4 DUS3L SRSF9 LIPT1 DDX5 MAX MRPL18 CTR9 MRPL16 NOLC1 MRPL12 MRPL10 MRPL11 PDCD11 BAG1 GAR1 MAP2K5 CTC1 EIF2B4 NOP14 CPSF6 PNPT1 SETD1B SIAH2 INTS2 ECD PA2G4 DNAJC14 MRPL21 DNAJC17 CLK2 PAN2 RPS25 RPL27A INTS5 INTS4 UBE2O TADA1

			MPHOSPH10 DUS1L HNRNPA1L2 RSL24D1 RPS24 RAD9A CREBZF USP36 MRPS16 UBXN1 MAST4 RPLP1 RPLP0 DDX41 SMG8 MRPL32 MRPL4 SNAPC5 MRPL42 MRPL2 MRPL1 SART3 SIN3A SNAPC4 MRPL9 ARIH1 USP30 RFC5 TOP3B CCT2 PRMT5 TSFM USP42 MRPS25 MARS2 MRPL49 DDX54 DDX51 ZDHHC17 RPUSD4 PDIK1L RPUSD2 TCP1 POLR1C TOP1 PFDN5 DCP2 CTDP1 PRIM1 DDX23 DDX21 CSTF2T MRPS30 USP19 CAMKK2 EXOSC7 PDF TAF1C RBBP5 TDG POLR2D LEO1 C10ORF2 CSK RPS2 RPS27A EIF4B GALNT6 SSB PPIL2 USP20 KRR1 USP21 MCRS1 RPL23A NPM3 PUS7L ACVR2B TRIT1 ADAT2 RSL1D1 EIF3M SPCS2 POLR3A NFIA POLR3B WT1 TRMT5 MARS EIF3J RPS6KB2 RPL13AP3 FAU RBMX METAP2
RNA metabolic process	9.0764E-18	4.1275E-15	CCNT1 PDCD7 EIF4A3 ARID4A DDX41 TSEN2 RPL10A SMG8 TSEN54 CCAR1 SNAPC5 RPS17 HELB MRPL1 SART3 ALKBH1 SNAPC4 PUS3 PRMT5 AQR IMP3 METTL1 PUS1 MARS2 DDX54 IMP4 DDX51 POLRMT GTPBP3 RPUSD4 LARS2 POLG2 CLNS1A RPUSD2 POLR1C TFAM PPIH TARS2 SNRPE SNRPF GEMIN8 DUS3L DCP2 SRSF9 DDX5 MAX PRIM1 DDX23 DDX21 CSTF2T NOLC1 MRPL12 EXOSC7 PDCD11 TAF1C POLR2D GAR1 C10ORF2 NOP14 SSB CPSF6 PNPT1 KRR1 INTS2 ECD PA2G4 NPM3 PUS7L TRIT1 ADAT2 PAN2 RSL1D1 POLR3A WT1 TRMT5 MARS INTS5 INTS4 MPHOSPH10 DUS1L HNRNPA1L2 RBMX RPS24
Cellular metabolic process	8.8340E-17	3.2138E-14	RPL32 EIF4A3 NADSYN1 TSEN54 RPL6 CCAR1 NDST2 RPS17 TRIM28 HERC1 KAT5 ALKBH1 STRA13 FBXO6 ATXN7L3 RPS13 AQR ENDOG IMP3 METTL1 OXSM RSAD1 COG2 IMP4 PHKA2 CLPX GTPBP3 MRPS18C LARS2 MTHFD2 PPA1 CLNS1A TRIM13 TFAM TARS2 UQCRC2 DUS3L LIPT1 MRPL18 CTR9 MRPL16 NOLC1 MRPL12 MRPL10 MRPL11 NEU3 ATP5B PDCD11 GAR1 CTC1 SETD1B SIAH2 INTS2 PA2G4 MRPL21 CS RPS25 RPL27A INTS5 INTS4 MPHOSPH10 NDUFAF1 DUS1L HNRNPA1L2 RPS24 MRPS16 UBXN1 RPLP1 RPLP0 COX7C SMG8 MRPL32 MRPL42 ARIH1 DLAT TOP3B CCT2 PRMT5 MRPS25 MRPL49 ZDHHC17 RPUSD4 PDIK1L RPUSD2 TCP1 ALDOC DCP2 CTDP1 PRIM1 ATP5A1 ODC1 GDPD1 MRPS30 CAMKK2 EXOSC7 PDF LEO1 C10ORF2 RPS2 RPS27A GCH1 MDH2 PTGES2 MCRS1 NPM3 PUS7L ADAT2 RSL1D1 SPCS2 NFIA WT1 TRMT5 MARS NDUFAB1 FAU CCNT1 PDCD7 RPL10L ARID4A YEATS4 TSEN2 COX6A1 RPL10A PPP1CC HELB RUVBL1 PIM2 MEN1 PUS3 RPS9 USP7 KMT5C EIF1AX RPS5 USP3

			PUS1 SDHD CHD1L ATP1B2 POLRMT TUFM OXA1L POLG2 TSSK6 PPIH SNRPE SNRPF GEMIN8 FKBP4 SLC27A5 ISCU SRSF9 DDX5 NDUFB10 MAX NDUFB11 IREB2 BAG1 NDUFV1 MAP2K5 EIF2B4 NOP14 PDHA1 CPSF6 PNPT1 EPHX2 ECD DNAJC14 DNAJC17 CLK2 PAN2 COQ3 UBE2O TADA1 RSL24D1 RAD9A CREBZF USP36 MAST4 DDX41 ATP5G3 ATP5G2 MRPL4 SNAPC5 MRPL2 ZNRD1 MRPL1 SART3 SIN3A ENOSF1 SNAPC4 MRPL9 USP30 RFC5 TSFM USP42 MARS2 DCXR DDX54 DDX51 POLR1C NDUFS3 TOP1 SLC25A12 PFDN5 DDX23 DDX21 CSTF2T AMBRA1 USP19 MAT2A TAF1C RBBP5 TDG POLR2D CSK PDSS1 EIF4B GALNT6 SSB PPIL2 NDUFA5 USP20 KRR1 USP21 RPL23A ACVR2B TRIT1 RPIA EIF3M SLC25A39 POLR3A POLR3B EIF3J RPS6KB2 RPL13AP3 GALM RBMX METAP2
Ncrna metabolic process	3.0790E-16	9.3344E-14	EIF4A3 DDX21 NOLC1 TSEN2 TSEN54 EXOSC7 RPS17 PDCD11 GAR1 PUS3 NOP14 SSB IMP3 KRR1 METTL1 PUS1 MARS2 INTS2 IMP4 DDX51 PA2G4 NPM3 PUS7L TRIT1 GTPBP3 ADAT2 LARS2 POLG2 TRMT5 MARS INTS5 INTS4 MPHOSPH10 TARS2 DUS1L DUS3L RPS24
RNA processing	3.2348E-15	8.4060E-13	PDCD7 EIF4A3 DDX41 TSEN2 RPL10A TSEN54 CCAR1 RPS17 MRPL1 SART3 PUS3 PRMT5 AQR IMP3 METTL1 PUS1 DDX54 IMP4 DDX51 GTPBP3 CLNS1A PPIH SNRPE SNRPF GEMIN8 DUS3L SRSF9 DDX5 DDX23 DDX21 CSTF2T NOLC1 EXOSC7 PDCD11 POLR2D GAR1 NOP14 SSB CPSF6 PNPT1 KRR1 INTS2 PA2G4 NPM3 PUS7L TRIT1 ADAT2 RSL1D1 WT1 TRMT5 INTS5 INTS4 MPHOSPH10 DUS1L HNRNPA1L2 RBMX RPS24
Nucleic acid metabolic process	4.0042E-15	9.1046E-13	CCNT1 PDCD7 EIF4A3 ARID4A TSEN2 RPL10A TSEN54 CCAR1 RPS17 HELB KAT5 ALKBH1 RUVBL1 STRA13 FBXO6 MEN1 PUS3 AQR ENDOG IMP3 METTL1 USP3 PUS1 IMP4 CHD1L POLRMT GTPBP3 LARS2 POLG2 CLNS1A TFAM PPIH TARS2 SNRPE SNRPF GEMIN8 DUS3L SRSF9 DDX5 MAX NOLC1 MRPL12 PDCD11 GAR1 CTC1 NOP14 CPSF6 PNPT1 INTS2 ECD PA2G4 PAN2 INTS5 INTS4 MPHOSPH10 DUS1L HNRNPA1L2 RPS24 RAD9A CREBZF DDX41 SMG8 SNAPC5 MRPL1 SART3 SIN3A SNAPC4 RFC5 TOP3B PRMT5 MARS2 DDX54 DDX51 RPUSD4 RPUSD2 POLR1C TOP1 DCP2 PRIM1 DDX23 DDX21 CSTF2T EXOSC7 TAF1C TDG POLR2D C10ORF2 SSB KRR1 NPM3 PUS7L TRIT1 ADAT2 RSL1D1 POLR3A NFIA POLR3B WT1 TRMT5 MARS RBMX
Ncrna processing	6.5958E-15	1.3331E-12	EIF4A3 DDX21 NOLC1 TSEN2 TSEN54 EXOSC7 RPS17 PDCD11 GAR1 PUS3 NOP14 SSB IMP3 KRR1 METTL1 PUS1 INTS2 IMP4 DDX51 PA2G4 NPM3

			PUS7L TRIT1 GTPBP3 ADAT2 TRMT5 INTS5 INTS4 MPHOSPH10 DUS1L DUS3L RPS24
Cellular macromolecule biosynthetic process	1.2302E-14	2.2377E-12	CREBZF MRPS16 CCNT1 RPL32 RPLP1 RPLP0 RPL10L ARID4A RPL10A MRPL32 RPL6 NDST2 MRPL4 SNAPC5 MRPL42 MRPL2 RPS17 HELB MRPL1 SIN3A SNAPC4 MRPL9 RPS13 RFC5 TOP3B TSFM RPS9 EIF1AX IMP3 MRPS25 RPS5 MARS2 MRPL49 COG2 POLRMT ZDHHC17 MRPS18C TUFM LARS2 POLG2 POLR1C TFAM TARS2 TOP1 MAX MRPL18 PRIM1 MRPL16 MRPS30 MRPL12 MRPL10 MRPL11 PDF TAF1C POLR2D C10ORF2 RPS2 RPS27A EIF4B GALNT6 EIF2B4 ECD RPL23A NPM3 MRPL21 RPS25 RSL1D1 EIF3M POLR3A NFIA POLR3B RPL27A MARS EIF3J RPS6KB2 RPL13AP3 FAU RSL24D1 RPS24 RAD9A
Macromolecule metabolic process	1.5195E-14	2.5127E-12	RPL32 EIF4A3 TSEN54 RPL6 CCAR1 NDST2 RPS17 TRIM28 HERC1 KAT5 ALKBH1 STRA13 FBXO6 ATXN7L3 RPS13 AQR ENDOG IMP3 METTL1 COG2 IMP4 PHKA2 CLPX GTPBP3 MRPS18C LARS2 CLNS1A TRIM13 TFAM TARS2 UQCRC2 DUS3L LIPT1 MRPL18 CTR9 MRPL16 NOLC1 MRPL12 MRPL10 MRPL11 PDCD11 GAR1 CTC1 CRADD SETD1B SIAH2 INTS2 PA2G4 MRPL21 RPS25 RPL27A INTS5 INTS4 MPHOSPH10 DUS1L HNRNPA1L2 RPS24 MRPS16 UBXN1 RPLP1 RPLP0 SMG8 MRPL32 MMP20 MRPL42 ARIH1 TOP3B CCT2 PRMT5 MRPS25 MRPL49 ZDHHC17 RPUSD4 PDIK1L RPUSD2 TCP1 DCP2 CTDP1 PRIM1 MRPS30 CAMKK2 EXOSC7 PDF LEO1 C10ORF2 RPS2 RPS27A MCRS1 NPM3 PUS7L ADAT2 RSL1D1 SPCS2 NFIA WT1 TRMT5 MARS FAU CCNT1 PDCD7 RPL10L ARID4A YEATS4 TSEN2 RPL10A PPP1CC HELB RUVBL1 PIM2 MEN1 PUS3 RPS9 USP7 KMT5C EIF1AX RPS5 USP3 PUS1 CHD1L POLRMT TUFM POLG2 TSSK6 PPIH SNRPE SNRPF GEMIN8 FKBP4 SRSF9 DDX5 MAX DPP3 BAG1 MAP2K5 EIF2B4 NOP14 CPSF6 PNPT1 ECD DNAJC14 DNAJC17 CLK2 PAN2 IL4 UBE2O TADA1 RSL24D1 RAD9A CREBZF USP36 MAST4 DDX41 MRPL4 SNAPC5 MRPL2 MRPL1 SART3 SIN3A SNAPC4 MRPL9 USP30 RFC5 TSFM USP42 MARS2 DDX54 DDX51 ADAMTS20 ESPL1 POLR1C TOP1 PFDN5 DDX23 DDX21 CSTF2T USP19 TAF1C RBBP5 TDG POLR2D CSK EIF4B GALNT6 SSB PPIL2 USP20 KRR1 USP21 RPL23A ACVR2B TRIT1 EIF3M POLR3A POLR3B EIF3J RPS6KB2 RPL13AP3 RBMX METAP2
Nucleobase, nucleoside, nucleotide and nucleic acid metabolic process	3.2589E-14	4.9399E-12	CCNT1 PDCD7 EIF4A3 ARID4A TSEN2 RPL10A NADSYN1 TSEN54 CCAR1 RPS17 HELB KAT5 ALKBH1 RUVBL1 STRA13 FBXO6 MEN1 PUS3 AQR ENDOG IMP3 METTL1 USP3 PUS1 IMP4 CHD1L

			ATP1B2 POLRMT GTPBP3 LARS2 POLG2 CLNS1A TFAM PPIH TARS2 SNRPE SNRPF GEMIN8 DUS3L SRSF9 DDX5 MAX NOLC1 MRPL12 ATP5B PDCD11 GAR1 CTC1 NOP14 CPSF6 PNPT1 INTS2 ECD PA2G4 PAN2 INTS5 INTS4 MPHOSPH10 DUS1L HNRNPA1L2 RPS24 RAD9A CREBZF DDX41 ATP5G3 ATP5G2 SMG8 SNAPC5 ZNRD1 MRPL1 SART3 SIN3A SNAPC4 RFC5 TOP3B PRMT5 MARS2 DCXR DDX54 DDX51 RPUSD4 RPUSD2 POLR1C TOP1 DCP2 PRIM1 ATP5A1 DDX23 DDX21 CSTF2T EXOSC7 MAT2A TAF1C TDG POLR2D C10ORF2 SSB GCH1 MDH2 KRR1 NPM3 PUS7L TRIT1 ADAT2 RPIA RSL1D1 POLR3A NFIA POLR3B WT1 TRMT5 MARS RBMX
Macromolecule biosynthetic process	3.7166E-14	5.2004E-12	CREBZF MRPS16 CCNT1 RPL32 RPLP1 RPLP0 RPL10L ARID4A RPL10A MRPL32 RPL6 NDST2 MRPL4 SNAPC5 MRPL42 MRPL2 RPS17 HELB MRPL1 SIN3A SNAPC4 MRPL9 RPS13 RFC5 TOP3B TSFM RPS9 EIF1AX IMP3 MRPS25 RPS5 MARS2 MRPL49 COG2 POLRMT ZDHHC17 MRPS18C TUFM LARS2 POLG2 POLR1C TFAM TARS2 TOP1 MAX MRPL18 PRIM1 MRPL16 MRPS30 MRPL12 MRPL10 MRPL11 PDF TAF1C POLR2D C10ORF2 RPS2 RPS27A EIF4B GALNT6 EIF2B4 ECD RPL23A NPM3 MRPL21 RPS25 RSL1D1 EIF3M POLR3A NFIA POLR3B RPL27A MARS EIF3J RPS6KB2 RPL13AP3 FAU RSL24D1 RPS24 RAD9A
Metabolic process	3.9599E-12	5.1451E-10	RPL32 EIF4A3 NADSYN1 TSEN54 RPL6 CCAR1 NDST2 RPS17 ALKBH4 TRIM28 HERC1 KAT5 ALKBH1 OGFOD2 STRA13 FBXO6 ATXN7L3 RPS13 AQR ENDOG IMP3 METTL1 OXSM RSAD1 COG2 IMP4 PHKA2 CLPX GTPBP3 MRPS18C LARS2 MTHFD2 PPA1 CLNS1A TRIM13 TFAM TARS2 UQCRC2 DUS3L GCDH LIPT1 MRPL18 CTR9 MRPL16 NOLC1 MRPL12 MRPL10 MRPL11 NEU3 ATP5B PDCD11 RDH16 GAR1 RDH13 CTC1 CRADD SETD1B SIAH2 INTS2 PA2G4 MRPL21 CS RPS25 RPL27A INTS5 INTS4 MPHOSPH10 NDUFAF1 DUS1L HNRNPA1L2 RPS24 DHRS13 MRPS16 UBXN1 RPLP1 RPLP0 NAT10 COX7C SMG8 MRPL32 MMP20 MRPL42 ARIH1 DLAT TOP3B CCT2 SREBF1 PRMT5 MRPS25 MRPL49 HEXDC ZDHHC17 RPUSD4 PDIK1L RPUSD2 TCP1 ALDOC DCP2 CTDP1 PRIM1 ATP5A1 ODC1 GDPD1 MRPS30 CAMKK2 EXOSC7 PDF LEO1 C10ORF2 RPS2 RPS27A GCH1 MDH2 PTGES2 MCRS1 NPM3 PUS7L ADAT2 RSL1D1 SPCS2 NFIA WT1 TRMT5 MARS NDUFAB1 FAU CCNT1 PDCD7 RPL10L ARID4A YEATS4 TSEN2 COX6A1 RPL10A PPP1CC HELB RUVBL1 PIM2 MEN1 PUS3 RPS9 USP7 KDM2B KMT5C EIF1AX RPS5 USP3 PUS1

			SDHD CHD1L ATP1B2 POLRMT TUFM OXA1L POLG2 TSSK6 PPIH SNRPE SNRPF GEMIN8 FKBP4 SLC27A5 ISCU SRSF9 DDX5 NDUFB10 MAX NDUFB11 IREB2 DPP3 BAG1 NDUFV1 MAP2K5 EIF2B4 NOP14 PDHA1 CPSF6 PNPT1 EPHX2 ECD DNAJC14 DNAJC17 UCKL1 CLK2 PAN2 IL4 COQ3 UBE2O TADA1 RSL24D1 RAD9A CREBZF USP36 MAST4 DDX41 ATP5G3 ATP5G2 MRPL4 SNAPC5 MRPL2 ZNRD1 MRPL1 SART3 SIN3A ENOSF1 SNAPC4 MRPL9 USP30 RFC5 TSFM USP42 MARS2 DCXR DDX54 DDX51 ADAMTS20 ESPL1 POLR1C NDUFS3 TOP1 SLC25A12 PFDN5 DDX23 DDX21 CSTF2T AMBRA1 USP19 MAT2A TAF1C RBBP5 TDG POLR2D CSK PDSS1 EIF4B GALNT6 SSB PPIL2 NDUFA5 USP20 KRR1 USP21 RPL23A ACVR2B TRIT1 RPIA EIF3M SLC25A39 POLR3A POLR3B EIF3J RPS6KB2 RPL13AP3 GALM RBMX METAP2
Ribonucleoprotein complex biogenesis	1.2550E-11	1.5039E-9	RPLP0 EIF4A3 DDX23 DDX21 NOLC1 MRPL10 EXOSC7 RPS17 PDCD11 GAR1 PRMT5 NOP14 IMP3 KRR1 IMP4 DDX51 PA2G4 NPM3 RPS25 GNL3L CLNS1A MPHOSPH10 SNRPE SNRPF GEMIN8 RSL24D1 RPS24 SRSF9
Cellular nitrogen compound metabolic process	1.3229E-11	1.5039E-9	CCNT1 PDCD7 EIF4A3 ARID4A TSEN2 RPL10A NADSYN1 TSEN54 CCAR1 RPS17 HELB KAT5 ALKBH1 RUVBL1 STRA13 FBXO6 MEN1 PUS3 AQR ENDOG IMP3 METTL1 USP3 PUS1 RSAD1 IMP4 CHD1L ATP1B2 POLRMT GTPBP3 LARS2 POLG2 CLNS1A TFAM PPIH TARS2 SNRPE SNRPF GEMIN8 DUS3L SRSF9 DDX5 MAX IREB2 NOLC1 MRPL12 ATP5B PDCD11 GAR1 CTC1 NOP14 CPSF6 PNPT1 INTS2 ECD PA2G4 PAN2 INTS5 INTS4 MPHOSPH10 DUS1L HNRNPA1L2 RPS24 RAD9A CREBZF DDX41 ATP5G3 ATP5G2 SMG8 SNAPC5 ZNRD1 MRPL1 SART3 SIN3A ENOSF1 SNAPC4 RFC5 TOP3B PRMT5 MARS2 DCXR DDX54 DDX51 RPUSD4 RPUSD2 POLR1C TOP1 DCP2 PRIM1 ATP5A1 ODC1 DDX23 DDX21 CSTF2T EXOSC7 MAT2A TAF1C TDG POLR2D C10ORF2 SSB GCH1 MDH2 KRR1 NPM3 PUS7L TRIT1 ADAT2 RPIA RSL1D1 SLC25A39 POLR3A NFIA POLR3B WT1 TRMT5 MARS RBMX
Cellular biosynthetic process	3.0138E-11	3.2247E-9	CCNT1 RPL32 RPL10L ARID4A RPL10A NADSYN1 RPL6 NDST2 RPS17 HELB RPS13 RPS9 EIF1AX IMP3 RPS5 OXSM RSAD1 COG2 ATP1B2 POLRMT MRPS18C TUFM LARS2 POLG2 MTHFD2 TFAM TARS2 SLC27A5 ISCU MAX MRPL18 IREB2 MRPL16 MRPL12 MRPL10 MRPL11 ATP5B EIF2B4 ECD MRPL21 RPS25 COQ3 RPL27A RSL24D1 RPS24 RAD9A CREBZF MRPS16 RPLP1 RPLP0 ATP5G3 ATP5G2 MRPL32 MRPL4 SNAPC5 MRPL42 MRPL2

			MRPL1 SIN3A SNAPC4 MRPL9 DLAT RFC5 TOP3B TSFM MRPS25 MARS2 MRPL49 ZDHHC17 POLR1C TOP1 PRIM1 ATP5A1 ODC1 MRPS30 PDF MAT2A TAF1C POLR2D C10ORF2 PDSS1 RPS2 RPS27A EIF4B GALNT6 GCH1 PTGES2 RPL23A NPM3 RSL1D1 EIF3M SLC25A39 POLR3A NFIA POLR3B MARS EIF3J NDUFAB1 RPS6KB2 RPL13AP3 FAU
Nitrogen compound metabolic process	1.0468E-10	1.0578E-8	CCNT1 PDCD7 EIF4A3 ARID4A TSEN2 RPL10A NADSYN1 TSEN54 CCAR1 RPS17 HELB KAT5 ALKBH1 RUVBL1 STRA13 FBXO6 MEN1 PUS3 AQR ENDOG IMP3 METTL1 USP3 PUS1 RSAD1 IMP4 CHD1L ATP1B2 POLRMT GTPBP3 LARS2 POLG2 MTHFD2 CLNS1A TFAM PPIH TARS2 SNRPE SNRPF GEMIN8 DUS3L ISCU SRSF9 DDX5 MAX IREB2 NOLC1 MRPL12 ATP5B PDCD11 GAR1 CTC1 NOP14 CPSF6 PNPT1 INTS2 ECD PA2G4 PAN2 INTS5 INTS4 MPHOSPH10 DUS1L HNRNPA1L2 RPS24 RAD9A CREBZF DDX41 ATP5G3 ATP5G2 SMG8 SNAPC5 ZNRD1 MRPL1 SART3 SIN3A ENOSF1 SNAPC4 RFC5 TOP3B PRMT5 MARS2 DCXR DDX54 DDX51 RPUSD4 RPUSD2 POLR1C TOP1 DCP2 PRIM1 ATP5A1 ODC1 DDX23 DDX21 CSTF2T EXOSC7 MAT2A TAF1C TDG POLR2D C10ORF2 SSB GCH1 MDH2 KRR1 NPM3 PUS7L TRIT1 ADAT2 RPIA RSL1D1 SLC25A39 POLR3A NFIA POLR3B WT1 TRMT5 MARS RBMX
Primary metabolic process	2.4765E-10	2.3710E-8	RPL32 EIF4A3 NADSYN1 TSEN54 RPL6 CCAR1 RPS17 TRIM28 HERC1 KAT5 ALKBH1 STRA13 FBXO6 ATXN7L3 RPS13 AQR ENDOG IMP3 METTL1 OXSM COG2 IMP4 PHKA2 CLPX GTPBP3 MRPS18C LARS2 CLNS1A TRIM13 TFAM TARS2 UQCRC2 DUS3L LIPT1 MRPL18 CTR9 MRPL16 NOLC1 MRPL12 MRPL10 MRPL11 NEU3 ATP5B PDCD11 RDH16 GAR1 CTC1 CRADD SETD1B SIAH2 INTS2 PA2G4 MRPL21 CS RPS25 RPL27A INTS5 INTS4 MPHOSPH10 DUS1L HNRNPA1L2 RPS24 MRPS16 UBXN1 RPLP1 RPLP0 SMG8 MRPL32 MMP20 MRPL42 ARIH1 DLAT TOP3B CCT2 SREBF1 PRMT5 MRPS25 MRPL49 HEXDC ZDHHC17 RPUSD4 PDIK1L RPUSD2 TCP1 ALDOC DCP2 CTDP1 PRIM1 ATP5A1 ODC1 GDPD1 MRPS30 CAMKK2 EXOSC7 PDF LEO1 C10ORF2 RPS2 RPS27A GCH1 MDH2 PTGES2 MCRS1 NPM3 PUS7L ADAT2 RSL1D1 SPCS2 NFIA WT1 TRMT5 MARS NDUFAB1 FAU CCNT1 PDCD7 RPL10L ARID4A YEATS4 TSEN2 RPL10A PPP1CC HELB RUVBL1 PIM2 MEN1 PUS3 RPS9 USP7 KMT5C EIF1AX RPS5 USP3 PUS1 CHD1L ATP1B2 POLRMT TUFM POLG2 TSSK6 PPIH SNRPE SNRPF GEMIN8 FKBP4 SLC27A5 SRSF9 DDX5 MAX DPP3 BAG1 MAP2K5 EIF2B4 NOP14 PDHA1 CPSF6 PNPT1 ECD

			DNAJC14 DNAJC17 CLK2 PAN2 IL4 COQ3 UBE2O TADA1 RSL24D1 RAD9A CREBZF USP36 MAST4 DDX41 ATP5G3 ATP5G2 MRPL4 SNAPC5 MRPL2 ZNRD1 MRPL1 SART3 SIN3A ENOSF1 SNAPC4 MRPL9 USP30 RFC5 TSFM USP42 MARS2 DCXR DDX54 DDX51 ADAMTS20 ESPL1 POLR1C TOP1 PFDN5 DDX23 DDX21 CSTF2T USP19 MAT2A TAF1C RBBP5 TDG POLR2D CSK PDSS1 EIF4B GALNT6 SSB PPIL2 USP20 KRR1 USP21 RPL23A ACVR2B TRIT1 RPIA EIF3M POLR3A POLR3B EIF3J RPS6KB2 RPL13AP3 GALM RBMX METAP2
Ribosome biogenesis	6.1257E-10	5.5713E-8	NOP14 IMP3 KRR1 RPLP0 EIF4A3 DDX21 NOLC1 IMP4 DDX51 PA2G4 NPM3 MRPL10 EXOSC7 RPS25 RPS17 GNL3L PDCD11 GAR1 MPHOSPH10 RSL24D1 RPS24
Translational elongation	9.1096E-10	7.8907E-8	TSFM RPS9 RPL32 RPLP1 RPS5 RPLP0 RPL23A RPL10A RPL6 TUFM RPS25 RPS17 RPL27A RPS2 FAU RPS27A RPS13 RPS24
Biosynthetic process	1.0958E-9	9.0604E-8	CCNT1 RPL32 RPL10L ARID4A RPL10A NADSYN1 RPL6 NDST2 RPS17 HELB RPS13 RPS9 EIF1AX IMP3 RPS5 OXSM RSAD1 COG2 ATP1B2 POLRMT MRPS18C TUFM LARS2 POLG2 MTHFD2 TFAM TARS2 SLC27A5 ISCU MAX MRPL18 IREB2 MRPL16 MRPL12 MRPL10 MRPL11 ATP5B EIF2B4 ECD MRPL21 RPS25 COQ3 RPL27A RSL24D1 RPS24 RAD9A CREBZF MRPS16 RPLP1 RPLP0 ATP5G3 ATP5G2 MRPL32 MRPL4 SNAPC5 MRPL42 MRPL2 MRPL1 SIN3A SNAPC4 MRPL9 DLAT RFC5 TOP3B TSFM MRPS25 MARS2 MRPL49 ZDHHC17 POLR1C TOP1 PRIM1 ATP5A1 ODC1 MRPS30 PDF MAT2A TAF1C POLR2D C10ORF2 PDSS1 RPS2 RPS27A EIF4B GALNT6 GCH1 PTGES2 RPL23A NPM3 RSL1D1 EIF3M SLC25A39 POLR3A NFIA POLR3B MARS EIF3J NDUFAB1 RPS6KB2 RPL13AP3 FAU
tRNA metabolic process	2.3311E-8	1.8436E-6	PUS3 SSB METTL1 PUS1 MARS2 TSEN2 PUS7L TSEN54 TRIT1 GTPBP3 ADAT2 LARS2 POLG2 TRMT5 MARS TARS2 DUS1L DUS3L
rRNA processing	4.5567E-8	3.4536E-6	NOP14 IMP3 KRR1 EIF4A3 DDX21 NOLC1 IMP4 DDX51 PA2G4 NPM3 EXOSC7 RPS17 PDCD11 GAR1 MPHOSPH10 RPS24
Cellular protein metabolic process	6.8048E-8	4.9512E-6	CCNT1 RPL32 RPL10L YEATS4 RPL10A RPL6 RPS17 TRIM28 HERC1 KAT5 RUVBL1 FBXO6 PIM2 ATXN7L3 RPS13 RPS9 USP7 KMT5C EIF1AX IMP3 RPS5 USP3 COG2 PHKA2 CLPX MRPS18C TUFM LARS2 POLG2 TRIM13 TSSK6 PPIH TARS2 FKBP4 LIPT1 MRPL18 CTR9 MRPL16 MRPL12 MRPL10 MRPL11 BAG1 MAP2K5 EIF2B4 SETD1B SIAH2 DNAJC14 MRPL21 DNAJC17 CLK2 PAN2 RPS25 RPL27A UBE2O TADA1 RSL24D1 RPS24 USP36 MRPS16 UBXN1 MAST4 RPLP1 RPLP0 MRPL32 MRPL4 MRPL42 MRPL2 MRPL1 MRPL9 ARIH1

			USP30 CCT2 PRMT5 TSFM USP42 MRPS25 MARS2 MRPL49 ZDHHC17 PDIK1L TCP1 PFDN5 CTDP1 MRPS30 USP19 CAMKK2 PDF RBBP5 LEO1 CSK RPS2 RPS27A EIF4B GALNT6 PPIL2 USP20 USP21 MCRS1 RPL23A ACVR2B RSL1D1 EIF3M SPCS2 MARS EIF3J RPS6KB2 RPL13AP3 FAU METAP2
Rrna metabolic process	8.1978E-8	5.7353E-6	NOP14 IMP3 KRR1 EIF4A3 DDX21 NOLC1 IMP4 DDX51 PA2G4 NPM3 EXOSC7 RPS17 PDCD11 GAR1 MPHOSPH10 RPS24
Chromatin organization	6.5285E-7	4.3983E-5	KMT2D SMARCD2 CTR9 ARID4A YEATS4 MBTD1 KAT5 VPS72 RBBP5 BAHD1 RUVBL1 LEO1 HIST1H2AE ARID2 ATXN7L3 HIST1H3D MEN1 BPTF PRMT5 KDM2B UTP3 KMT5C USP21 SETD1B USP3 INO80 CHD1L HIST2H2BE HIST3H2A TSSK6 TADA1 BCOR HIST1H2BC
Trna processing	7.4276E-7	4.8253E-5	PUS3 SSB METTL1 PUS1 TSEN2 PUS7L TSEN54 TRIT1 GTPBP3 ADAT2 TRMT5 DUS1L DUS3L
Chromatin modification	4.7524E-6	2.9809E-4	KMT2D SMARCD2 CTR9 YEATS4 MBTD1 KAT5 VPS72 RBBP5 BAHD1 RUVBL1 LEO1 ARID2 ATXN7L3 MEN1 BPTF PRMT5 KDM2B UTP3 KMT5C USP21 SETD1B USP3 INO80 CHD1L TADA1 BCOR
Transcription from mitochondrial promoter	1.3144E-5	7.9698E-4	C10ORF2 TFAM MRPL12 POLRMT
Chromosome organization	1.7736E-5	1.0407E-3	KMT2D SMARCD2 CTR9 ARID4A YEATS4 MBTD1 KAT5 VPS72 RBBP5 BAHD1 RUVBL1 LEO1 HIST1H2AE ARID2 ATXN7L3 HIST1H3D MEN1 BPTF CTC1 PRMT5 KDM2B UTP3 KMT5C USP21 SETD1B USP3 INO80 CHD1L HIST2H2BE HIST3H2A ESPL1 TSSK6 TADA1 BCOR HIST1H2BC
Protein metabolic process	1.8459E-5	1.0493E-3	CCNT1 RPL32 RPL10L YEATS4 RPL10A RPL6 RPS17 TRIM28 HERC1 KAT5 RUVBL1 FBXO6 PIM2 ATXN7L3 RPS13 RPS9 USP7 KMT5C EIF1AX IMP3 RPS5 USP3 COG2 PHKA2 CLPX MRPS18C TUFM LARS2 POLG2 TRIM13 TSSK6 PPIH TARS2 UQCRC2 FKBP4 LIPT1 MRPL18 CTR9 MRPL16 MRPL12 MRPL10 MRPL11 DPP3 BAG1 MAP2K5 EIF2B4 CRADD SETD1B SIAH2 ECD DNAJC14 MRPL21 DNAJC17 CLK2 PAN2 RPS25 RPL27A UBE2O TADA1 RSL24D1 RPS24 USP36 MRPS16 UBXN1 MAST4 RPLP1 RPLP0 MRPL32 MMP20 MRPL4 MRPL42 MRPL2 MRPL1 MRPL9 ARIH1 USP30 CCT2 PRMT5 TSFM USP42 MRPS25 MARS2 MRPL49 ZDHHC17 ADAMTS20 ESPL1 PDIK1L TCP1 PFDN5 CTDP1 MRPS30 USP19 CAMKK2 PDF RBBP5 LEO1 CSK RPS2 RPS27A EIF4B GALNT6 PPIL2 USP20 USP21 MCRS1 RPL23A ACVR2B RSL1D1 EIF3M SPCS2 MARS EIF3J RPS6KB2 RPL13AP3 FAU METAP2
Regulation of gene	2.2308E-5	1.2297E-3	ZNF692 CCNT1 CD80 TACO1 EIF4A3 ARID4A

expression			YEATS4 IKZF4 RPL6 IKZF5 CCAR1 TRIM28 KAT5 ALKBH1 MYC RUVBL1 MYB DPF2 STRA13 ZNF721 ARID2 ATXN7L3 RPS13 MEN1 EOMES ZBTB39 USP7 ANKRD49 KDM2B ZNF282 KMT5C IMP3 RPS5 USP3 PUS1 NGDN ILF2 MED6 PIAS1 BRMS1 ZNF92 SUB1 TFAM ZNF678 ATF5 ZFPM1 ZNF397 KMT2D DDX5 MAX IREB2 MRPL12 MBTD1 HESX1 PPARGC1B GCN1 BPTF ESRRA WNT10B EIF2B4 SETD1B ECD PA2G4 MED13L IL4 ZFP62 TADA1 MDM4 CREBZF ZNF770 RNF10 LDB1 ZC3H8 SNAPC5 BCL7A NKRF ZNF408 VPS72 SIN3A SNAPC4 ZNF766 NELFA ZNF641 ZNF385A SREBF1 ZNF121 PRMT5 TGIF2 DDX54 CBFA2T3 REXO4 MED28 PFDN5 ZNF595 SMARCD2 RRN3 ZNF592 ZBTB48 TXLNG MTIF2 ZNF8 ZBTB40 CAMKK2 BCLAF1 TAF1C RBBP5 BAHD1 LEO1 PLAGL2 ZNF589 NCAM2 EIF4B ZNF740 ZNF581 UTP3 PTGES2 USP21 CCDC59 IRX4 GATC ACVR2B ZNF33B NR6A1 NFIA WT1 RPS6KB2 BCOR
Regulation of macromolecule biosynthetic process	2.4649E-5	1.3187E-3	ZNF692 CCNT1 CD80 TACO1 EIF4A3 ARID4A YEATS4 IKZF4 RPL6 IKZF5 CCAR1 TRIM28 KAT5 MYC RUVBL1 MYB DPF2 STRA13 ZNF721 ARID2 ATXN7L3 MEN1 EOMES ZBTB39 USP7 ANKRD49 KDM2B ZNF282 KMT5C IMP3 RPS5 PUS1 NGDN ILF2 MED6 PIAS1 BRMS1 ZNF92 SUB1 TFAM ZNF678 ATF5 ZFPM1 ZNF397 KMT2D DDX5 MAX IREB2 MRPL12 MBTD1 HESX1 PPARGC1B GCN1 BPTF ESRRA WNT10B EIF2B4 SETD1B ECD PA2G4 MED13L IL4 ZFP62 TADA1 MDM4 RAD9A CREBZF ZNF770 RNF10 LDB1 ZC3H8 SNAPC5 BCL7A NKRF ZNF408 VPS72 SIN3A SNAPC4 ZNF766 NELFA ZNF641 ZNF385A SREBF1 ZNF121 PRMT5 TGIF2 DDX54 CBFA2T3 REXO4 MED28 PFDN5 ZNF595 SMARCD2 RRN3 ZNF592 ZBTB48 TXLNG MTIF2 ZNF8 ZBTB40 CAMKK2 BCLAF1 TAF1C RBBP5 BAHD1 LEO1 PLAGL2 ZNF589 NCAM2 EIF4B ZNF740 ZNF581 PTGES2 USP21 CCDC59 IRX4 GATC ACVR2B ZNF33B MAS1 NR6A1 NFIA WT1 RPS6KB2 BCOR
Cellular process	3.0587E-5	1.5896E-3	NUP107 RPL32 EIF4A3 WIPF3 NADSYN1 TSEN54 RPL6 CCAR1 NDST2 RPS17 TRIM28 HERC1 KAT5 ALKBH1 RASSF5 MYC DPF2 STRA13 FBXO6 ATXN7L3 RPS13 AQR ENDOG IMP3 METTL1 OXSM RSAD1 TOMM20L COG2 IMP4 PHKA2 CLPX GTPBP3 MRPS18C LARS2 MTHFD2 PPA1 CLNS1A TRIM13 TFAM TARS2 UQCRC2 ATF5 DUS3L LIPT1 POM121 EPB41 MRPL18 CTR9 PEX11B MRPL16 NOLC1 AAAS MRPL12 MRPL10 MRPL11 MBTD1 NEU3 ATP5B SNX1 POM121C PDCD11 POM121B GAR1 SEC31B HIST1H3D CTC1 WNT10B SETD1B SIAH2 INTS2 PA2G4 INO80

			MRPL21 CS RPS25 RPL27A NDUFAF4 INTS5 MKS1 SFXN4 INTS4 MPHOSPH10 NDUFAF1 DUS1L HNRNPA1L2 RPS24 ARF3 C11ORF31 MRPS16 UBXN1 FASTKD1 RPLP1 RPLP0 COX7C SMG8 MRPL32 MRPL42 SSSCA1 ARIH1 DLAT OFD1 TOP3B CCT2 SREBF1 PRMT5 MRPS25 MRPL49 CBFA2T3 ZDHHC17 HIST2H2BE RPUSD4 KIF9 PDIK1L RPUSD2 TCP1 ALDOC DCP2 CTDP1 PRIM1 ATP5A1 ODC1 GDPD1 TXLNG MRPS30 CAMKK2 AKAP1 EXOSC7 FGD3 GGA3 PDF LEO1 C10ORF2 RPS2 RPS27A PEX16 GCH1 UTP3 MDH2 PTGES2 MCRS1 NPM3 PUS7L ADAT2 LETM1 RSL1D1 MAS1 SPCS2 HIST3H2A NFIA XPOT WT1 TRMT5 MARS NDUFAB1 FAU MTCH2 CCNT1 PDCD7 CD80 RPL10L ARID4A YEATS4 TSEN2 COX6A1 RPL10A IPO4 CHCHD4 PPP1CC HELB RUVBL1 HIST1H2AE PIM2 KPNA5 ARID2 MEN1 EOMES PUS3 RPS9 USP7 KDM2B KMT5C EIF1AX VPS33A RPS5 USP3 PUS1 SDHD CHD1L ATP1B2 POLRMT DGKZ PIAS1 TUFM OXA1L POLG2 TSSK6 PPIH SNRPE SNRPF GEMIN8 FKBP4 SLC27A5 ISCU SRSF9 HIST1H2BC DAP3 KMT2D DDX5 NDUFB10 MAX NDUFB11 IREB2 NUP160 NOP9 BAG1 NDUFV1 MAP2K5 BPTF EIF2B4 NOP14 PDHA1 CPSF6 PNPT1 EPHX2 ECD TIMM44 DNAJC14 DNAJC17 CLK2 PAN2 IL4 COQ3 GNL3L UBE2O TADA1 MDM4 RSL24D1 RAD9A LMBR1L CREBZF RNF10 USP36 MAST4 LDB1 ZC3H8 TRIAP1 DDX41 ATP5G3 ATP5G2 RND1 MRPL4 SNAPC5 MRPL2 ZNRD1 MRPL1 SART3 VPS72 SIN3A ENOSF1 SNAPC4 MRPL9 USP30 RFC5 TSFM TGIF2 USP42 GLRX5 MARS2 DCXR DDX54 DDX51 ARAP1 ARFGAP2 ADAMTS20 SUPV3L1 ESPL1 SLC25A19 POLR1C NDUFS3 CDH12 TOP1 COL6A5 SLC25A12 PFDN5 SLC25A6 SMARCD2 DDX23 DDX21 CSTF2T AMBRA1 USP19 TBRG4 MAT2A TAF1C RBBP5 BAHD1 TDG POLR2D CSK TNNI3 PDSS1 NCAM2 EIF4B GALNT6 SSB PPIL2 NDUFA5 USP20 KRR1 USP21 RPL23A TSPEAR ACVR2B TRIT1 RPIA EIF3M SLC25A39 POLR3A POLR3B EIF3J RPS6KB2 RPL13AP3 PDCD2 GALM BCOR RBM45 SEC24C RBMX METAP2
Cellular respiration	4.1144E-5	2.0789E-3	CS OXA1L NDUFA5 NDUFB10 MDH2 NDUFAB1 NDUFS3 UQCRC2 NDUFAF1 SDHD SLC25A12 NDUFV1
Generation of precursor metabolites and energy	4.3169E-5	2.1223E-3	PDHA1 NDUFA5 NDUFB10 MDH2 NDUFB11 ATP5A1 SDHD ATP5G3 COX6A1 ATP5G2 PHKA2 COX7C CS ATP5B PPP1CC OXA1L NDUFAB1 NDUFS3 ALDOC UQCRC2 NDUFAF1 DLAT SLC25A12 NDUFV1
Pseudouridine	4.5011E-5	2.1546E-3	RPUSD4 PUS3 RPUSD2 PUS1 PUS7L

synthesis			
Regulation of cellular biosynthetic process	6.3008E-5	2.9387E-3	ZNF692 CCNT1 CD80 TACO1 EIF4A3 ARID4A YEATS4 IKZF4 RPL6 IKZF5 CCAR1 TRIM28 KAT5 MYC RUVBL1 MYB DPF2 STRA13 ZNF721 ARID2 ATXN7L3 MEN1 EOMES ZBTB39 USP7 ANKRD49 KDM2B ZNF282 KMT5C IMP3 RPS5 PUS1 NGDN ILF2 MED6 PIAS1 BRMS1 ZNF92 SUB1 TFAM ZNF678 ATF5 ZFPM1 ZNF397 KMT2D DDX5 MAX IREB2 MRPL12 NOP9 MBTD1 HESX1 PPARGC1B GCN1 BPTF ESRRA WNT10B EIF2B4 PDHA1 SETD1B ECD PA2G4 MED13L IL4 ZFP62 TADA1 MDM4 RAD9A CREBZF ZNF770 RNF10 LDB1 ZC3H8 SNAPC5 BCL7A NKRF ZNF408 VPS72 SIN3A SNAPC4 ZNF766 NELFA DLAT ZNF641 ZNF385A SREBF1 ZNF121 PRMT5 TGIF2 DDX54 CBFA2T3 REXO4 MED28 PFDN5 ZNF595 SMARCD2 RRN3 ZNF592 ZBTB48 TXLNG MTIF2 ZNF8 ZBTB40 CAMKK2 BCLAF1 TAF1C RBBP5 BAHD1 LEO1 PLAGL2 ZNF589 NCAM2 EIF4B ZNF740 ZNF581 PTGES2 USP21 CCDC59 IRX4 GATC ACVR2B ZNF33B MAS1 NR6A1 NFIA WT1 RPS6KB2 BCOR
Regulation of biosynthetic process	9.1099E-5	4.1427E-3	ZNF692 CCNT1 CD80 TACO1 EIF4A3 ARID4A YEATS4 IKZF4 RPL6 IKZF5 CCAR1 TRIM28 KAT5 MYC RUVBL1 MYB DPF2 STRA13 ZNF721 ARID2 ATXN7L3 MEN1 EOMES ZBTB39 USP7 ANKRD49 KDM2B ZNF282 KMT5C IMP3 RPS5 PUS1 NGDN ILF2 MED6 PIAS1 BRMS1 ZNF92 SUB1 TFAM ZNF678 ATF5 ZFPM1 ZNF397 KMT2D DDX5 MAX IREB2 MRPL12 NOP9 MBTD1 HESX1 PPARGC1B GCN1 BPTF ESRRA WNT10B EIF2B4 PDHA1 SETD1B ECD PA2G4 MED13L IL4 ZFP62 TADA1 MDM4 RAD9A CREBZF ZNF770 RNF10 LDB1 ZC3H8 SNAPC5 BCL7A NKRF ZNF408 VPS72 SIN3A SNAPC4 ZNF766 NELFA DLAT ZNF641 ZNF385A SREBF1 ZNF121 PRMT5 TGIF2 DDX54 CBFA2T3 REXO4 MED28 PFDN5 ZNF595 SMARCD2 RRN3 ZNF592 ZBTB48 TXLNG MTIF2 ZNF8 ZBTB40 CAMKK2 BCLAF1 TAF1C RBBP5 BAHD1 LEO1 PLAGL2 ZNF589 NCAM2 EIF4B ZNF740 ZNF581 PTGES2 USP21 CCDC59 IRX4 GATC ACVR2B ZNF33B MAS1 NR6A1 NFIA WT1 RPS6KB2 BCOR
Cellular component biogenesis	1.1355E-4	5.0376E-3	RPLP0 EIF4A3 SNAPC5 RPS17 HIST1H2AE OFD1 PRMT5 IMP3 IMP4 DDX51 HIST2H2BE PIAS1 OXA1L CLNS1A TCP1 TFAM PPIH SNRPE ALDOC SNRPF GEMIN8 FKBP4 SRSF9 HIST1H2BC MAX DDX23 DDX21 NOLC1 MRPL10 EXOSC7 FGD3 PDCD11 BAHD1 POLR2D GAR1 C10ORF2 PDSS1 HIST1H3D PEX16 NOP14 GCH1 KRR1 PA2G4 NPM3 RPS25 GNL3L HIST3H2A NDUFAF4 MKS1

			MPHOSPH10 MDM4 NDUFAF1 RSL24D1 RPS24
RNA modification	1.3946E-4	6.0399E-3	RPUSD4 PUS3 SSB METTL1 RPUSD2 PUS1 PUS7L GTPBP3
Energy derivation by oxidation of organic compounds	1.7041E-4	7.1840E-3	NDUFA5 NDUFB10 MDH2 SDHD PHKA2 CS PPP1CC OXA1L NDUFAB1 NDUFS3 UQCRC2 NDUFAF1 SLC25A12 NDUFV1
Trna modification	1.7378E-4	7.1840E-3	PUS3 SSB METTL1 PUS1 GTPBP3
Translational initiation	1.8349E-4	7.4173E-3	EIF2B4 EIF3M RPS17 EIF1AX RPS5 EIF3J
Regulation of translation	2.4333E-4	9.5081E-3	EIF2B4 IMP3 TACO1 RPS5 NGDN IREB2 EIF4A3 GATC MTIF2 PA2G4 WT1 RPS6KB2 EIF4B GCN1
Regulation of transcription	2.4567E-4	9.5081E-3	ZNF692 CCNT1 CD80 ARID4A YEATS4 IKZF4 RPL6 IKZF5 CCAR1 TRIM28 KAT5 MYC RUVBL1 MYB DPF2 STRA13 ZNF721 ARID2 ATXN7L3 MEN1 EOMES ZBTB39 USP7 ANKRD49 KDM2B ZNF282 KMT5C PUS1 ILF2 MED6 PIAS1 BRMS1 ZNF92 SUB1 TFAM ZNF678 ATF5 ZFPM1 ZNF397 KMT2D DDX5 MAX MRPL12 MBTD1 HESX1 PPARGC1B BPTF ESRRA WNT10B SETD1B ECD PA2G4 MED13L IL4 ZFP62 TADA1 MDM4 CREBZF ZNF770 RNF10 LDB1 ZC3H8 SNAPC5 BCL7A NKRF ZNF408 VPS72 SIN3A SNAPC4 ZNF766 NELFA ZNF641 ZNF385A SREBF1 ZNF121 PRMT5 TGIF2 DDX54 CBFA2T3 REXO4 MED28 PFDN5 ZNF595 SMARCD2 RRN3 ZNF592 ZBTB48 TXLNG ZNF8 ZBTB40 CAMKK2 BCLAF1 TAF1C RBBP5 BAHD1 LEO1 PLAGL2 ZNF589 NCAM2 ZNF740 ZNF581 PTGES2 USP21 CCDC59 IRX4 ACVR2B ZNF33B NR6A1 NFIA WT1 BCOR
RNA splicing	2.5670E-4	9.7279E-3	PRMT5 DDX5 AQR PDCD7 EIF4A3 DDX23 DDX41 TSEN2 TSEN54 CCAR1 WT1 CLNS1A POLR2D MPHOSPH10 PPIH SNRPE SNRPF GEMIN8 HNRNPA1L2 RBMX SRSF9
Oxidative phosphorylation	3.2322E-4	1.1999E-2	ATP5B NDUFA5 NDUFB10 ATP5A1 NDUFAB1 NDUFS3 UQCRC2 NDUFAF1 ATP5G3 ATP5G2 NDUFV1
Histone modification	5.1825E-4	1.8854E-2	PRMT5 KMT5C USP21 SETD1B USP3 CTR9 YEATS4 KAT5 RBBP5 RUVBL1 LEO1 TADA1 ATXN7L3
Mrna metabolic process	5.4614E-4	1.9479E-2	PRMT5 DDX5 AQR SSB CPSF6 PNPT1 EIF4A3 DDX23 CSTF2T DDX41 TSEN2 SMG8 TSEN54 PAN2 PDCD11 CLNS1A PPIH SNRPE SNRPF GEMIN8 HNRNPA1L2 RBMX DCP2 SRSF9
Covalent chromatin modification	6.3166E-4	2.2096E-2	PRMT5 KMT5C USP21 SETD1B USP3 CTR9 YEATS4 KAT5 RBBP5 RUVBL1 LEO1 TADA1 ATXN7L3
Cofactor metabolic process	6.4738E-4	2.2219E-2	GCH1 MDH2 OXSM DCXR IREB2 RSAD1 SDHD NADSYN1 CS RPIA COQ3 SLC25A39 MTHFD2 PDSS1 DLAT ISCU
Regulation of nucleobase, nucleoside, nucleotide and nucleic acid metabolic process	6.9588E-4	2.3441E-2	ZNF692 CCNT1 CD80 ARID4A YEATS4 IKZF4 RPL6 IKZF5 CCAR1 TRIM28 KAT5 MYC RUVBL1 MYB DPF2 STRA13 ZNF721 ARID2 ATXN7L3 RPS13 MEN1 EOMES ZBTB39 USP7 ANKRD49 KDM2B ZNF282 KMT5C PUS1 ILF2 MED6 PIAS1 MYL4

			OXA1L BRMS1 ZNF92 SUB1 TFAM ZNF678 ATF5 ZFPM1 ZNF397 KMT2D DDX5 MAX AGAP2 MRPL12 NOP9 MBTD1 HESX1 PPARGC1B BPTF ESRRA WNT10B SETD1B ECD PA2G4 MED13L IL4 ZFP62 TADA1 MDM4 RAD9A CREBZF ZNF770 RNF10 LDB1 ZC3H8 SNAPC5 BCL7A NKRF ZNF408 VPS72 SIN3A SNAPC4 ZNF766 NELFA ZNF641 ZNF385A SREBF1 ZNF121 PRMT5 TGIF2 DDX54 ARAP1 CBFA2T3 REXO4 MED28 ARFGAP2 PFDN5 ZNF595 SMARCD2 RRN3 ZNF592 ZBTB48 TXLNG ZNF8 ZBTB40 CAMKK2 FGD3 BCLAF1 TAF1C RBBP5 BAHD1 LEO1 PLAGL2 TNNI3 ZNF589 NCAM2 ZNF740 ZNF581 PTGES2 USP21 CCDC59 IRX4 ACVR2B ZNF33B MAS1 NR6A1 NFIA WT1 BCOR
Ribosomal small subunit biogenesis	7.1921E-4	2.3786E-2	RPS25 NOP14 RPS17 RPS24
Methionyl-trna aminoacylation	9.6395E-4	3.0070E-2	MARS MARS2
Regulation of translational fidelity	9.6395E-4	3.0070E-2	RPS5 GATC
Trna pseudouridine synthesis	9.6395E-4	3.0070E-2	PUS3 PUS1
Regulation of nitrogen compound metabolic process	9.7532E-4	3.0070E-2	ZNF692 CCNT1 CD80 ARID4A YEATS4 IKZF4 RPL6 IKZF5 CCAR1 TRIM28 KAT5 MYC RUVBL1 MYB DPF2 STRA13 ZNF721 ARID2 ATXN7L3 RPS13 MEN1 EOMES ZBTB39 USP7 ANKRD49 KDM2B ZNF282 KMT5C PUS1 ILF2 MED6 PIAS1 MYL4 OXA1L BRMS1 ZNF92 SUB1 TFAM ZNF678 ATF5 ZFPM1 ZNF397 KMT2D DDX5 MAX AGAP2 MRPL12 NOP9 MBTD1 HESX1 PPARGC1B BPTF ESRRA WNT10B SETD1B ECD PA2G4 MED13L IL4 ZFP62 TADA1 MDM4 RAD9A CREBZF ZNF770 RNF10 LDB1 ZC3H8 SNAPC5 BCL7A NKRF ZNF408 VPS72 SIN3A SNAPC4 ZNF766 NELFA ZNF641 ZNF385A SREBF1 ZNF121 PRMT5 TGIF2 DDX54 ARAP1 CBFA2T3 REXO4 MED28 ARFGAP2 PFDN5 ZNF595 SMARCD2 RRN3 ZNF592 ZBTB48 TXLNG ZNF8 ZBTB40 CAMKK2 FGD3 BCLAF1 TAF1C RBBP5 BAHD1 LEO1 PLAGL2 TNNI3 ZNF589 NCAM2 ZNF740 ZNF581 PTGES2 USP21 CCDC59 IRX4 ACVR2B ZNF33B MAS1 NR6A1 NFIA WT1 BCOR
Cofactor biosynthetic process	1.0027E-3	3.0400E-2	COQ3 SLC25A39 GCH1 MTHFD2 IREB2 RSAD1 PDSS1 DLAT NADSYN1 ISCU
Regulation of macromolecule metabolic process	1.1328E-3	3.3780E-2	ZNF692 CCNT1 CD80 TACO1 EIF4A3 ARID4A YEATS4 IKZF4 RPL6 IKZF5 CCAR1 TRIM28 KAT5 ALKBH1 MYC RUVBL1 MYB DPF2 STRA13 ZNF721 ARID2 ATXN7L3 RPS13 MEN1 EOMES ZBTB39 USP7 ANKRD49 KDM2B ZNF282 KMT5C IMP3 RPS5 USP3 PUS1 NGDN ILF2 MED6 PIAS1 BRMS1

			ZNF92 SUB1 TFAM ZNF678 ATF5 ZFPM1 ZNF397 KMT2D DDX5 MAX IREB2 MRPL12 MBTD1 HESX1 PPARGC1B GCN1 BPTF ESRRA WNT10B EIF2B4 SETD1B ECD PA2G4 MED13L IL4 ZFP62 UBE2O TADA1 MDM4 RAD9A CREBZF ZNF770 RNF10 UBXN1 LDB1 ZC3H8 SNAPC5 BCL7A NKRF ZNF408 VPS72 SIN3A SNAPC4 ZNF766 NELFA ZNF641 ZNF385A SREBF1 ZNF121 PRMT5 TGIF2 DDX54 CBFA2T3 REXO4 MED28 PFDN5 ZNF595 SMARCD2 RRN3 ZNF592 ZBTB48 TXLNG MTIF2 ZNF8 ZBTB40 CAMKK2 AKAP1 BCLAF1 TAF1C RBBP5 BAHD1 LEO1 PLAGL2 ZNF589 NCAM2 EIF4B ZNF740 ZNF581 FEM1A UTP3 PTGES2 USP21 CCDC59 IRX4 GATC ACVR2B ZNF33B MAS1 NR6A1 NFIA WT1 RPS6KB2 BCOR
Posttranscriptional regulation of gene expression	1.4476E-3	4.2472E-2	WNT10B EIF2B4 IMP3 TACO1 RPS5 USP3 NGDN IREB2 EIF4A3 GATC MTIF2 PA2G4 WT1 RPS6KB2 MDM4 EIF4B GCN1
Regulation of primary metabolic process	1.5748E-3	4.5469E-2	ZNF692 CCNT1 CD80 TACO1 EIF4A3 ARID4A YEATS4 IKZF4 RPL6 IKZF5 CCAR1 TRIM28 KAT5 MYC RUVBL1 MYB DPF2 STRA13 ZNF721 ARID2 ATXN7L3 RPS13 MEN1 EOMES ZBTB39 USP7 ANKRD49 KDM2B ZNF282 KMT5C IMP3 RPS5 PUS1 NGDN ILF2 MED6 PIAS1 MYL4 OXA1L BRMS1 ZNF92 SUB1 TFAM ZNF678 ATF5 ZFPM1 ZNF397 KMT2D DDX5 MAX IREB2 AGAP2 MRPL12 NOP9 MBTD1 HESX1 PPARGC1B GCN1 BPTF ESRRA WNT10B EIF2B4 SETD1B ECD PA2G4 MED13L IL4 ZFP62 UBE2O TADA1 MDM4 RAD9A CREBZF ZNF770 RNF10 UBXN1 LDB1 ZC3H8 SNAPC5 BCL7A NKRF ZNF408 VPS72 SIN3A SNAPC4 ZNF766 NELFA ZNF641 ZNF385A SREBF1 ZNF121 PRMT5 TGIF2 DDX54 ARAP1 CBFA2T3 REXO4 MED28 ARFGAP2 PFDN5 ZNF595 SMARCD2 RRN3 ZNF592 ZBTB48 TXLNG MTIF2 ZNF8 ZBTB40 CAMKK2 AKAP1 FGD3 BCLAF1 TAF1C RBBP5 BAHD1 LEO1 PLAGL2 TNNI3 ZNF589 NCAM2 EIF4B ZNF740 ZNF581 FEM1A PTGES2 USP21 CCDC59 IRX4 GATC ACVR2B ZNF33B MAS1 NR6A1 NFIA WT1 RPS6KB2 BCOR
**Molecular function**			
Structural constituent of ribosome	7.0104E-23	4.3254E-20	MRPS16 RPL32 MRPL18 RPLP1 RPLP0 RPL10L MRPL16 MRPS30 MRPL12 RPL10A MRPL10 MRPL32 RPL6 MRPL11 MRPL4 MRPL42 MRPL2 RPS17 MRPL1 MRPL9 RPS2 RPS27A RPS13 RPS9 MRPS25 RPS5 MRPL49 RPL23A MRPL21 MRPS18C RPS25 RSL1D1 RPL27A RPL13AP3 FAU RSL24D1 RPS24
RNA binding	1.8871E-18	5.8216E-16	CCNT1 RPLP1 RPLP0 EIF4A3 ZC3H8 DDX41 RPL10A RPL6 MRPL1 SART3 NKRF RPS13 PUS3 RPS9 AQR KDM2B EIF1AX IMP3 METTL1 DDX55

			RPS5 PUS1 DDX54 DDX51 ILF2 RPUSD4 SUPV3L1 RPUSD2 TUT1 SNRPE SNRPF DCP2 SRSF9 DDX28 DDX5 IREB2 MRPL16 DDX21 CSTF2T MRPL12 JAKMIP1 AKAP1 EXOSC7 EIF1AD DHX30 PDCD11 GAR1 RPS2 PPARGC1B EIF4B NOP14 SSB PNPT1 KRR1 SETD1B RPL23A PA2G4 LARP4 PUS7L MRPL21 DNAJC17 RPS25 RSL1D1 XPOT WT1 RPL27A MARS FAU RBM45 HNRNPA1L2 RBMX
Nucleic acid binding	2.4646E-16	5.0689E-14	ZNF692 CCNT1 EIF4A3 ARID4A YEATS4 TSEN2 RPL10A IKZF4 RPL6 IKZF5 CCAR1 MYC MYB HIST1H2AE STRA13 ZNF721 ARID2 RPS13 MEN1 EOMES PUS3 ZBTB39 RPS9 AQR ENDOG KDM2B ZNF282 EIF1AX IMP3 METTL1 RPS5 PUS1 CHD1L POLRMT LEMD3 ILF2 PIAS1 TUFM ZNF92 POLG2 SUB1 TFAM ZNF678 SNRPE SNRPF ATF5 DUS3L ZFPM1 SRSF9 HIST1H2BC ZNF397 KMT2D DDX5 MAX IREB2 MRPL16 MRPL12 EIF1AD SBNO1 DHX30 PDCD11 HESX1 DHX37 GAR1 PPARGC1B GCN1 HIST1H3D BPTF CTC1 ESRRA NOP14 PNPT1 SETD1B PA2G4 INO80 LARP4 MRPL21 DNAJC17 PAN2 RPS25 ZFP62 RPL27A HNRNPA1L2 CREBZF ZNF770 RNF10 LDB1 RPLP1 RPLP0 AKAP8L ZC3H8 DDX41 SNAPC5 ZNRD1 MRPL1 SART3 NKRF ZNF408 VPS72 SIN3A SNAPC4 ZNF766 ZNF641 ZNF385A RFC5 TOP3B SREBF1 ZNF121 ZC3H10 TSFM TGIF2 DDX55 DDX54 DDX51 CBFA2T3 HIST2H2BE REXO4 RPUSD4 SUPV3L1 RPUSD2 POLR1C TUT1 TOP1 DCP2 DDX28 ZNF595 ZNF592 ZBTB48 DDX23 DDX21 CSTF2T MTIF2 JAKMIP1 ZNF8 ZBTB40 AKAP1 EXOSC7 BCLAF1 PDF TAF1C RBBP5 BAHD1 TDG C10ORF2 PLAGL2 METTL5 ZNF589 RPS2 C3ORF33 NCAM2 EIF4B ZNF740 SSB ZNF581 KRR1 PTGES2 IRX4 RPL23A NPM3 PUS7L ZNF33B RSL1D1 POLR3A HIST3H2A NR6A1 NFIA POLR3B XPOT WT1 MARS PDCD2 BCOR FAU RBM45 RBMX
Structural molecule activity	2.1306E-7	3.2864E-5	MRPS16 RPL32 EPB41 MRPL18 RPLP1 RPLP0 RPL10L MRPL16 YEATS4 MRPS30 MRPL12 RPL10A MRPL10 MRPL32 RPL6 MRPL11 AKAP1 MRPL4 MRPL42 MRPL2 RPS17 MRPL1 MRPL9 RPS2 RPS27A RPS13 RPS9 MRPS25 RPS5 MRPL49 RPL23A TSPEAR MRPL21 MRPS18C MYL4 RPS25 RSL1D1 RPL27A RPL13AP3 FAU RSL24D1 RPS24 ISCU
Helicase activity	1.0223E-6	1.2616E-4	DDX28 DDX5 DDX55 EIF4A3 DDX23 DDX54 DDX21 DDX51 DDX41 INO80 CHD1L SUPV3L1 DHX30 HELB RUVBL1 DHX37 C10ORF2
Pseudouridine synthase activity	1.8959E-6	1.9496E-4	RPUSD4 PUS3 RPUSD2 PUS1 GAR1 PUS7L
Isomerase activity	9.9254E-6	8.0794E-4	TOP3B PUS3 PPIL2 PTGES2 PUS1 PUS7L RPIA RPUSD4 RPUSD2 ENOSF1 GAR1 PPIH GALM TOP1

			FKBP4
Purine NTP-dependent helicase activity	1.1785E-5	8.0794E-4	DDX28 DDX5 DDX55 EIF4A3 DDX23 DDX54 DDX21 DDX51 DDX41 CHD1L DHX30 HELB DHX37
ATP-dependent helicase activity	1.1785E-5	8.0794E-4	DDX28 DDX5 DDX55 EIF4A3 DDX23 DDX54 DDX21 DDX51 DDX41 CHD1L DHX30 HELB DHX37
RNA polymerase activity	2.2892E-5	1.2840E-3	ZNRD1 CTDP1 POLR3A POLR3B PRIM1 POLR1C POLR2D POLRMT
DNA-directed RNA polymerase activity	2.2892E-5	1.2840E-3	ZNRD1 CTDP1 POLR3A POLR3B PRIM1 POLR1C POLR2D POLRMT
Intramolecular transferase activity	1.0644E-4	5.2632E-3	RPUSD4 PUS3 RPUSD2 PUS1 GAR1 PUS7L
Ubiquitin thiolesterase activity	1.1089E-4	5.2632E-3	PAN2 OTUD5 USP36 USP7 USP42 USP20 USP21 USP3 USP30 USP19
Methyltransferase activity	6.0313E-4	2.6581E-2	KMT2D PRMT5 NSUN5 KMT5C METTL1 SETD1B METTL2A PRMT3 BCDIN3D COQ3 COMTD1 METTL13 TRMT5 METTL5
Transferase activity, transferring one-carbon groups	7.1975E-4	2.9606E-2	KMT2D PRMT5 NSUN5 KMT5C METTL1 SETD1B METTL2A PRMT3 BCDIN3D COQ3 COMTD1 METTL13 TRMT5 METTL5
Methionine-tRNA ligase activity	9.2735E-4	3.0718E-2	MARS MARS2
DNA binding	9.4209E-4	3.0718E-2	CREBZF ZNF770 RNF10 ZNF692 CCNT1 LDB1 AKAP8L ZC3H8 ARID4A YEATS4 IKZF4 RPL6 IKZF5 SNAPC5 NKRF ZNF408 VPS72 SIN3A MYC MYB SNAPC4 HIST1H2AE STRA13 ZNF766 ZNF721 ARID2 ZNF641 ZNF385A MEN1 EOMES RFC5 TOP3B ZBTB39 SREBF1 ZNF121 TGIF2 KDM2B ZNF282 PUS1 CHD1L POLRMT LEMD3 ILF2 CBFA2T3 HIST2H2BE REXO4 PIAS1 SUPV3L1 ZNF92 POLG2 SUB1 POLR1C TFAM ZNF678 TOP1 ATF5 ZFPM1 HIST1H2BC ZNF397 ZNF595 KMT2D ZNF592 MAX ZBTB48 ZNF8 ZBTB40 SBNO1 BCLAF1 PDF TAF1C RBBP5 BAHD1 TDG HESX1 C10ORF2 PLAGL2 ZNF589 NCAM2 HIST1H3D BPTF CTC1 ESRRA ZNF581 PTGES2 IRX4 PA2G4 INO80 ZNF33B POLR3A HIST3H2A NR6A1 NFIA POLR3B WT1 PDCD2 BCOR
Nucleotidyltransferase activity	9.5682E-4	3.0718E-2	ZNRD1 CTDP1 POLR3A PNPT1 POLG2 POLR3B PRIM1 POLR1C POLR2D TUT1 POLRMT
RNA helicase activity	9.9746E-4	3.0718E-2	DDX5 EIF4A3 DDX23 DDX54 DDX21
Catalytic activity	1.0297E-3	3.0718E-2	HDDC2 OTUD5 NARF EIF4A3 TSEN2 COX6A1 NADSYN1 NDST2 TATDN3 PPP1CC COMTD1 HELB ALKBH4 TRIM28 HERC1 KAT5 ALKBH1 RUVBL1 OGFOD2 FBXO6 PIM2 PUS3 USP7 ENDOG KDM2B KMT5C IMP3 METTL1 USP3 OXSM PUS1 RSAD1 IMP4 SDHD CHD1L ATP1B2 POLRMT PHKA2 ILF2 DGKZ CLPX PIAS1 GTPBP3 TUFM MYL4 LARS2 POLG2 MTHFD2 PPA1 TRIM13 TSSK6 PPIH TARS2 UQCRC2 FKBP4 DUS3L SLC27A5 KMT2D GCDH LIPT1 DDX5 NDUFB10

			METTL2A DPP3 SBNO1 NEU3 ATP5B DHX30 SPATA5L1 RDH16 DHX37 GAR1 RDH13 NDUFV1 MAP2K5 BRAP PDHA1 PNPT1 CRADD APOBEC3H SETD1B SIAH2 EPHX2 ECD BCDIN3D PRG4 INO80 UCKL1 CLK2 CS PAN2 COQ3 UBE2O DUS1L RAD9A ARF3 DHRS13 USP36 MAST4 FASTKD1 NAT10 DDX41 COX7C MMP20 RND1 ZNRD1 METTL13 ENOSF1 ARIH1 DLAT USP30 RFC5 TOP3B PRMT5 USP42 NSUN5 GLRX5 DDX55 MARS2 DCXR PRMT3 DDX54 DDX51 HEXDC ZDHHC17 REXO4 FAHD2B ADAMTS20 RPUSD4 SUPV3L1 KIF9 ESPL1 PDIK1L RPUSD2 POLR1C NDUFS3 TUT1 PGAM5 ALDOC TOP1 DCP2 DDX28 CTDP1 PRIM1 ATP5A1 ODC1 DDX23 GDPD1 DDX21 MTIF2 COX5A USP19 CAMKK2 TBRG4 EXOSC7 PDF MAT2A TDG POLR2D C10ORF2 METTL5 CSK PDSS1 C3ORF33 GALNT6 PPIL2 FEM1A NDUFA5 GCH1 USP20 MDH2 PTGES2 USP21 PUS7L ACVR2B TRIT1 ADAT2 RPIA SPCS2 POLR3A POLR3B TRMT5 MARS NDUFAB1 RPS6KB2 GALM METAP2
DNA helicase activity	1.0789E-3	3.0718E-2	SUPV3L1 HELB RUVBL1 C10ORF2 INO80 CHD1L
Thiolester hydrolase activity	1.0953E-3	3.0718E-2	PAN2 OTUD5 USP36 USP7 USP42 USP20 USP21 USP3 USP30 USP19
rRNA binding	1.1938E-3	3.2026E-2	RPS9 KDM2B IMP3 MRPL16 RPL23A
ATP-dependent RNA helicase activity	1.8567E-3	4.7732E-2	EIF4A3 DDX23 DDX54 DDX21

[1] *P* value was calculated using the hypergeometric test and corrected for multiple hypothesis testing (P <0.05) using the Benjamini-Hochberg false discovery rate (FDR) adjustment.

Figure S31. Network visualization of gene ontology (GO) enrichment analysis based on gene expression profiles that correlated with the cytotoxicity profile of **5** across the NCI-60 panel cell lines

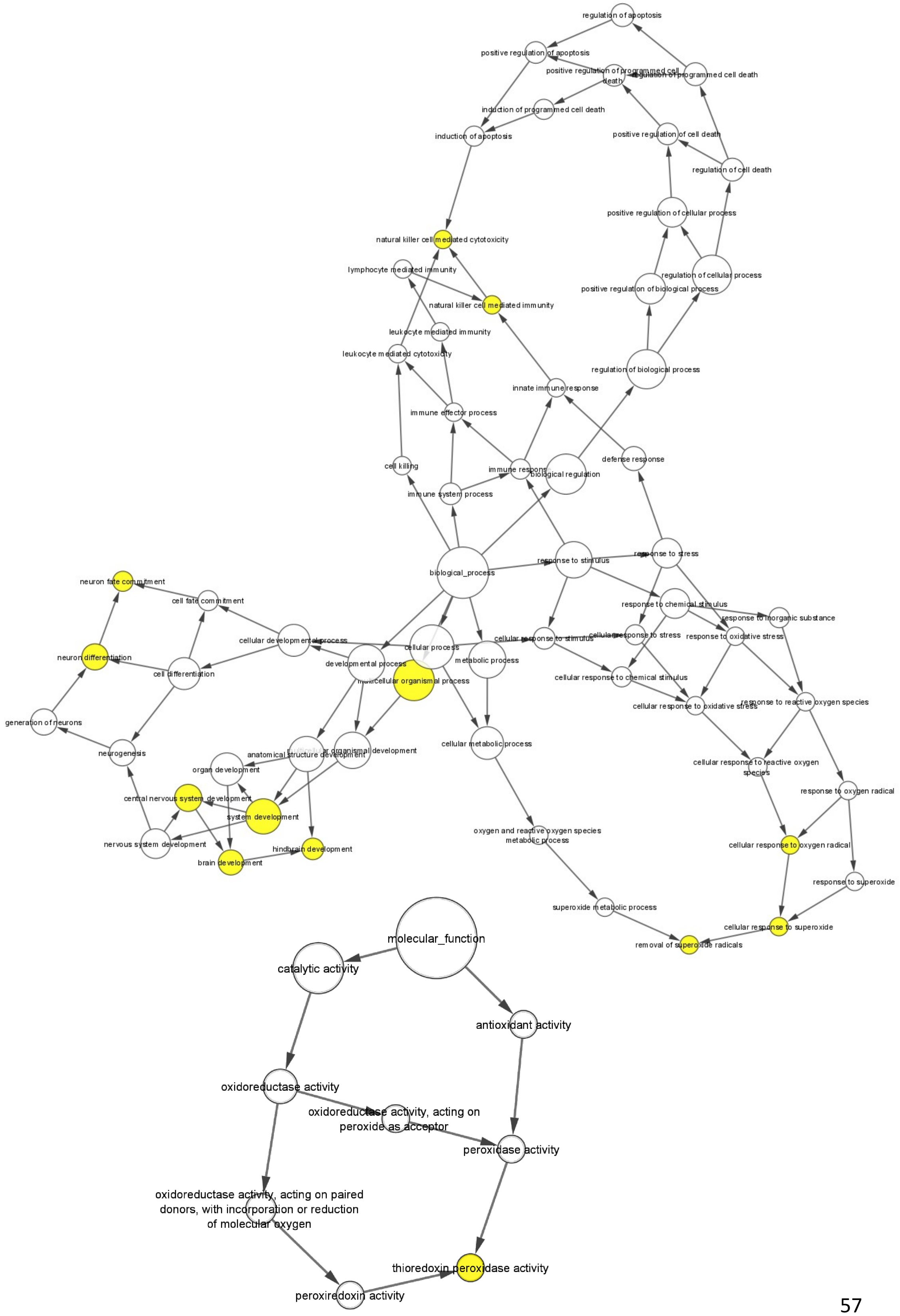

Figure S32. Network visualization of gene ontology (GO) enrichment analysis based on gene expression profiles that correlated with the cytotoxicity profile of **6** across the NCI-60 panel cell lines

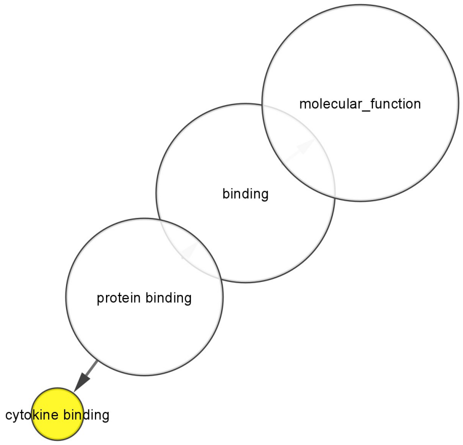

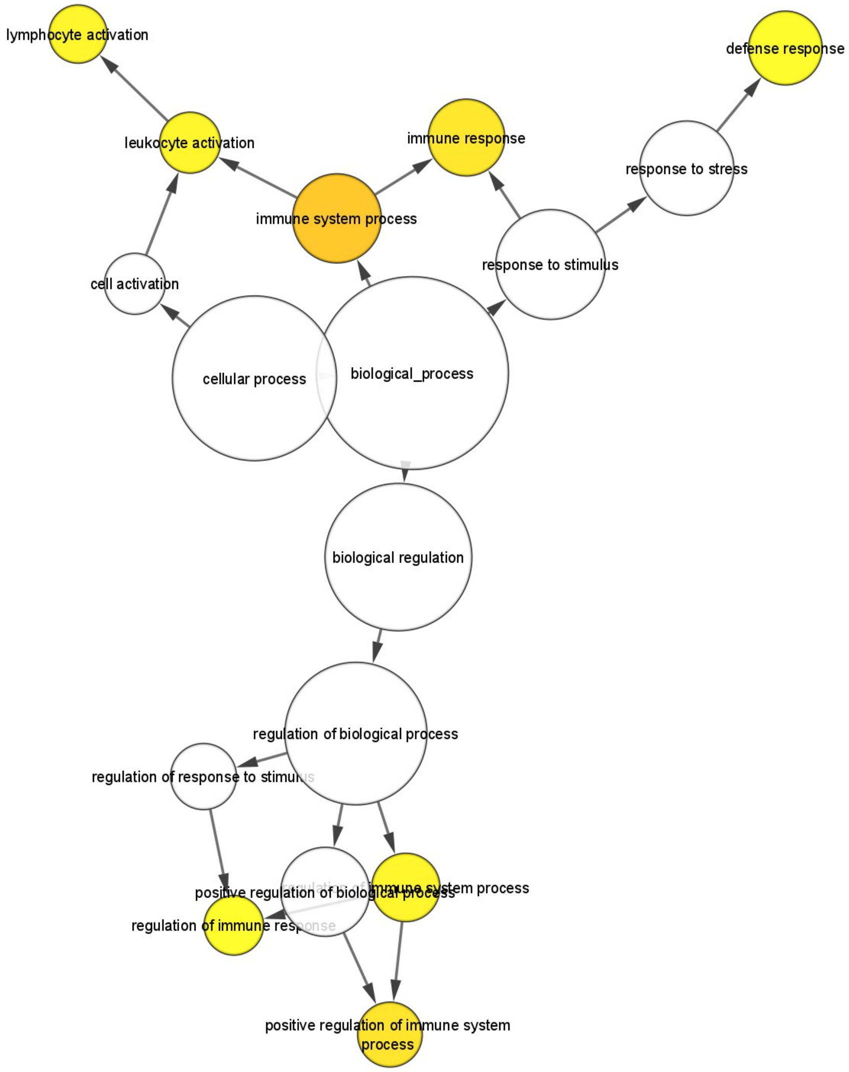

Figure S33. Network visualization of gene ontology (GO) enrichment analysis based on gene expression profiles that correlated with the cytotoxicity profile of **10** across the NCI-60 panel cell lines

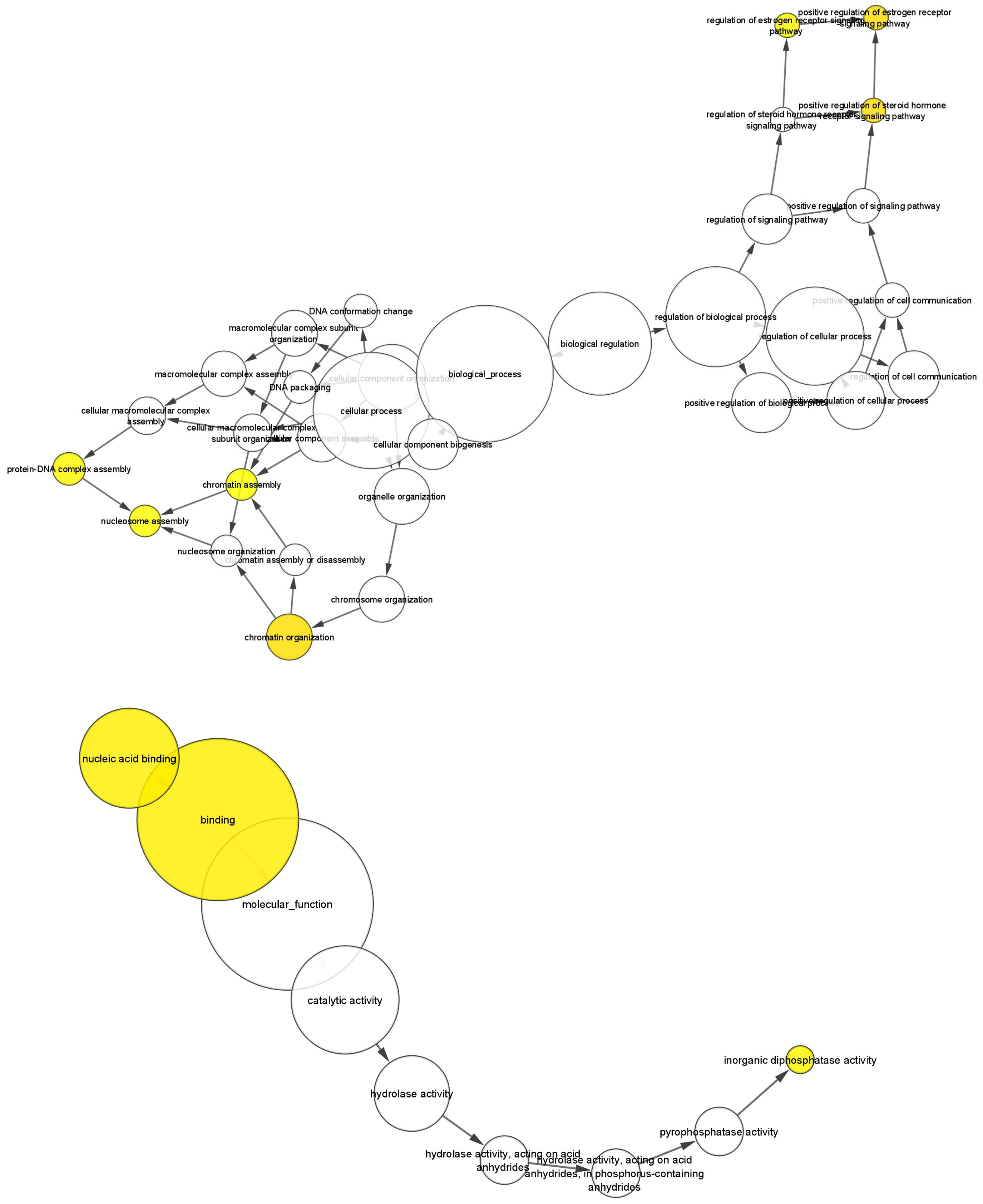

Figure S34. Network visualization of gene ontology (GO) enrichment analysis based on gene expression profiles that correlated with the cytotoxicity profile of **11** across the NCI-60 panel cell lines

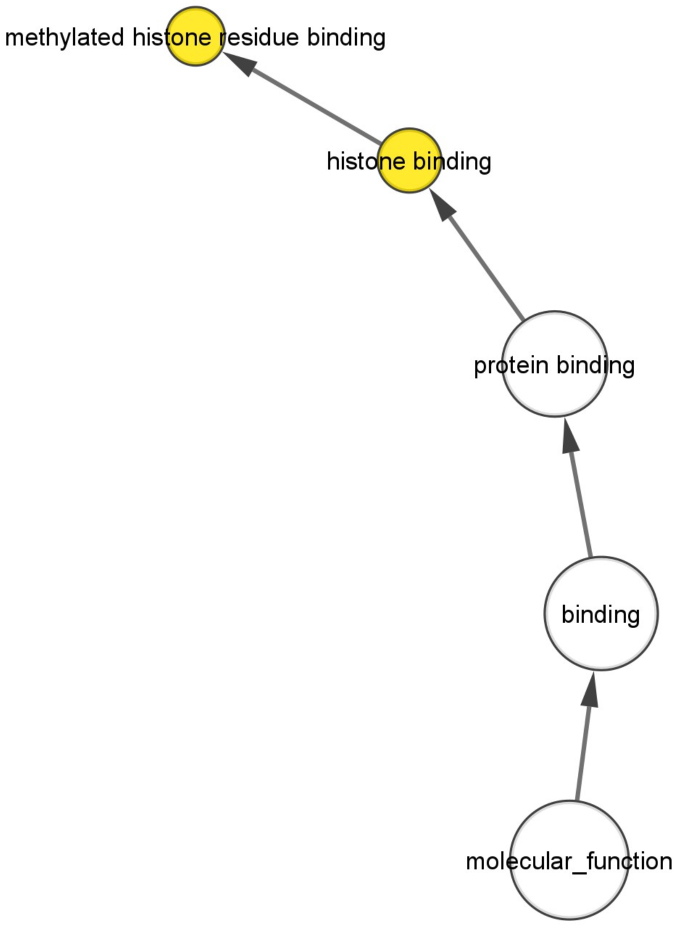

Figure S35. Network visualization of gene ontology (GO) enrichment analysis based on gene expression profiles that correlated with the cytotoxicity profile of **21** across the NCI-60 panel cell lines

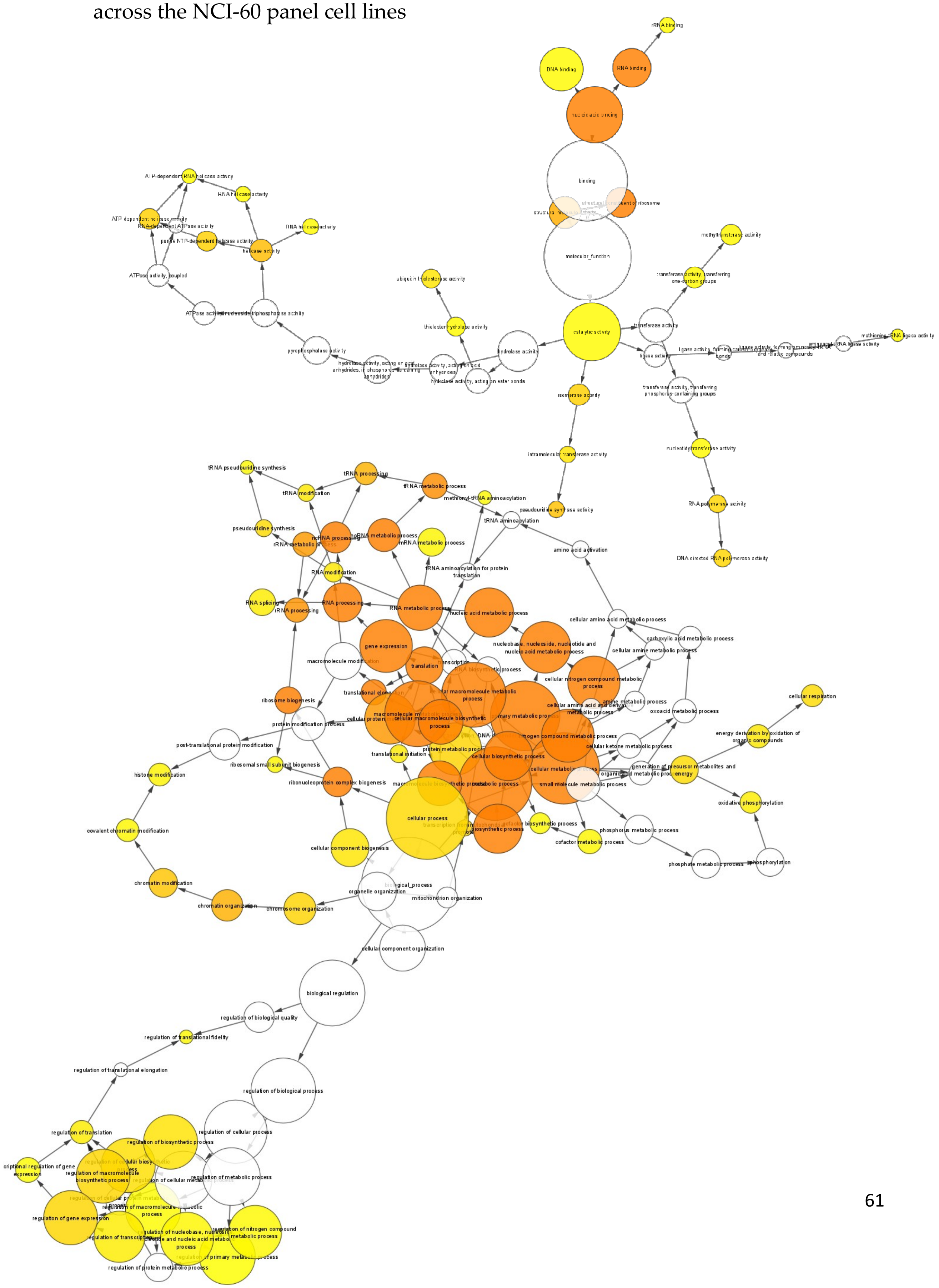

Supplement: Supplementary file 1 [file molecules-25-04833-s001.pdf]
